# Low-carbon ultra-high-performance concrete: raw materials, particle packing optimization, and data-driven performance assessment—a review

**DOI:** 10.1007/s44242-026-00116-x

**Published:** 2026-09-21

**Authors:** Ahmed Fageeri, Ousmane Hisseine

**Affiliations:** Department of Civil Engineering, McMaster University, 1280 Main St W, Hamilton, ON L8S 4L8 Canada

**Keywords:** CO_2_ emissions, Machine learning, Mixture design optimization, Particle packing design models, Supplementary cementitious materials (SCMs), Ultra-high-performance concrete (UHPC)

## Abstract

Ultra-high-performance concrete (UHPC) offers exceptional mechanical properties and durability; however, its cement-intensive formulations raise concerns regarding environmental sustainability. This study addresses this challenge by establishing quantitative relationships between UHPC mixture design, performance indicators, and embodied CO_2_ emissions by coupling a literature survey with data-driven analysis of over 200 mixtures. Unlike previous studies that examine the behavior of raw materials and the resulting performance in isolation, this study leverages data-driven approaches to link mixture design parameters to mechanical performance, durability indicators, and embodied CO_2_ emissions, thereby providing useful insights into low-carbon UHPC formulations. The influence of supplementary cementitious materials and mineral fillers is evaluated across rheological, mechanical, durability, and carbon footprint aspects. Machine learning (i.e., Random Forest) analysis demonstrates high accuracy in predicting compressive strength from mixture design parameters, while tensile properties and permeability remain influenced by microstructural factors not captured by mixture design parameters. Heat map correlation reveals strong interdependencies among mechanical and durability performance indicators, with compressive strength highly correlated (95%–98%) across curing ages and inversely related to permeability-related durability metrics. An exponential relationship between cement-to-binder and water-to-cement ratios is established (*R*^2^ of 95%), providing a strong predictive tool for mixture design. From a carbon footprint perspective, cement is the dominant contributor to CO_2_ emissions, as expected. Interestingly, cement content above 800 kg/m^3^ has negligible contributions to mechanical properties while significantly increasing the embodied CO_2_ emissions. By adopting a data-driven approach, this study provides novel insights into designing UHPC mixtures that foster higher mechanical and durability performance while reducing embodied carbon.

**Graphical Abstract:**

**Figure:**
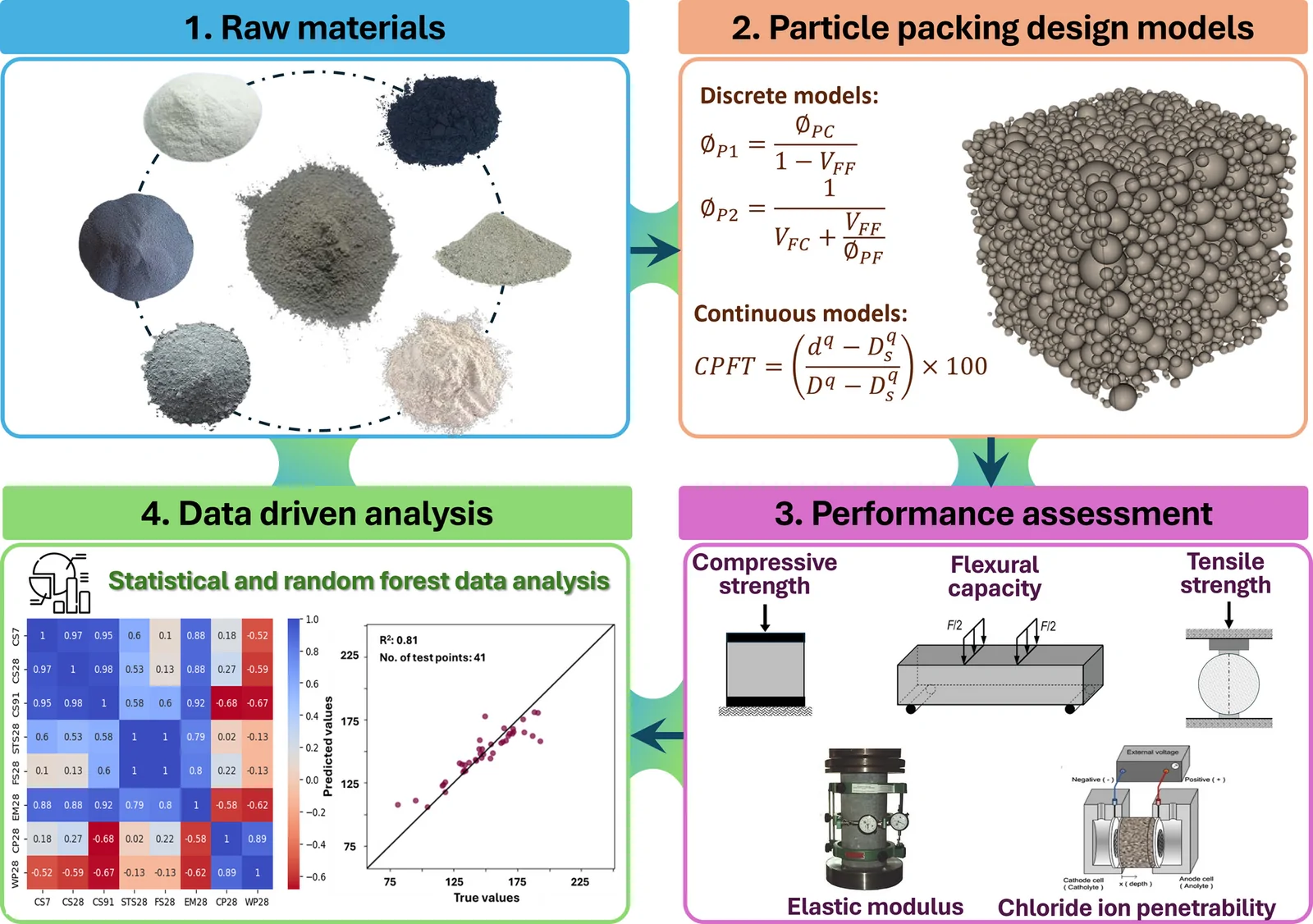

**Supplementary Information:**

The online version contains supplementary material available at https://doi.org/10.1007/s44242-026-00116-x.

## Introduction

**Fig. 1:**
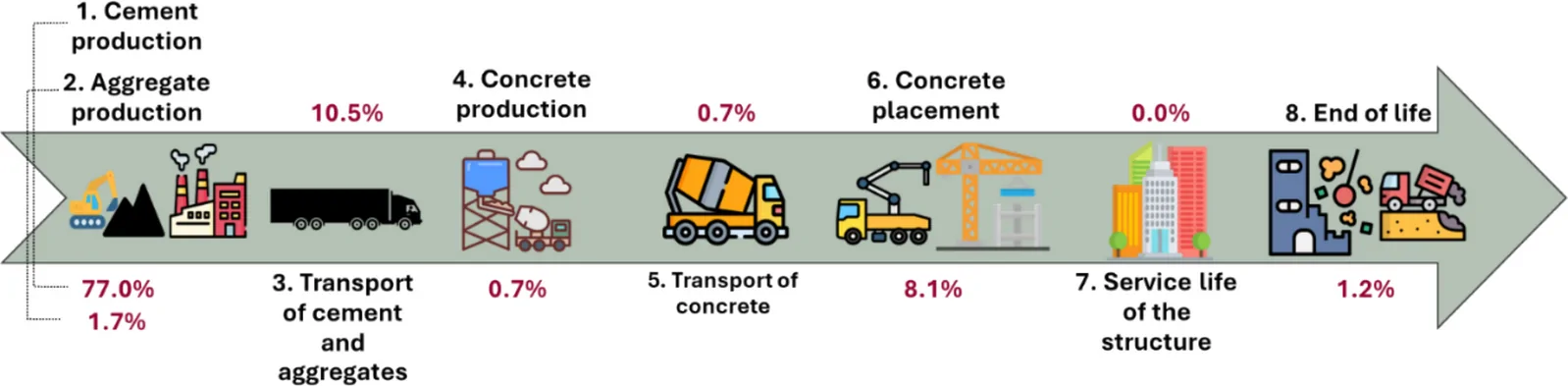
Life cycle CO_2_ emissions breakdown associated with concrete structures (Author-generated figure using data from [1]. Due to rounding, the sum of the individual percentages may differ slightly from 100%)

Whereas concrete represents the most widely used construction material globally [1], its eco-efficiency is currently challenged by the high CO_2_ emissions associated with cement production, which accounts for the majority of concrete’s embodied carbon (Fig. 1). At the same time, the increasing demand for durable and resilient infrastructure further challenges conventional concrete, whose long-term performance under aggressive environmental conditions often leads to premature deterioration and significant maintenance burdens [2].

In this context, while ultra-high-performance concrete’s (UHPC) superior mechanical properties and durability [3] are key for extending infrastructure service life and reducing maintenance, UHPC typically relies on cement-intensive formulations (in the range of 800–1 000 kg/m^3^) [4]. As a result, from a formulation standpoint, the environmental burden associated with UHPC can exceed that of conventional concrete, creating a challenging trade-off between performance and eco-efficiency [4, 5].

Achieving low-carbon UHPC (LC-UHPC) is inherently a multi-variable design problem that extends beyond simple cement replacement and is controlled by the types and replacement levels of supplementary cementitious materials (SCMs). The incorporation of SCMs—such as silica fume (SF), fly ash (FA), and metakaolin (MK)—provides an effective means to reduce clinker content through a combination of filler and pozzolanic effects, thus contributing to microstructural refinement and improved mechanical and durability performance [6]. In parallel, the use of inert mineral fillers alongside the implementation of particle packing design models (PPDMs) is key to improving packing density, thereby reducing voids and improving material efficiency [7]. PPDMs have been widely used to guide UHPC mixture design by optimizing the granular skeleton of the matrix [8]; although, the predictive accuracy of PPDMs may be influenced by their underlying assumptions, such as particle shape, interparticle interactions, and the influence of the liquid phase [9].

While the literature is replete with UHPC reviews on various individual aspects of UHPC, most of existing review studies predominantly focus on either raw materials, mechanical and durability properties, or mixture design approaches, and embodied carbon in isolation. For instance, recent review studies have focused on the influence of SCMs on the durability of UHPC [10], proposed technical–economic-environmental roadmaps without quantitative analysis linking them to UHPC’s key compositional and performance indicators [11], or applied a life-cycle assessment framework for UHPC decontextualized from UHPC’s controlling compositional and performance indicators [12]. Data-driven and machine learning approaches have been widely applied to predict the mechanical properties of conventional concrete [13, 14], and more recently to UHPC, where recent work has reviewed machine learning applications for predicting UHPC workability, mechanical, and thermal properties [15]. However, these efforts remain focused on algorithm development and prediction accuracy, with less attention paid to linking mixture design to UHPC’s key compositional and performance indicators. Therefore, limited attention has been paid to integrating raw material selection, particle packing optimization, mechanical and durability performance, and environmental impact within a single, data-driven framework.

This study presents an integrated, data-driven review of LC-UHPC by linking input from mixture design, particle packing models, mechanical and durability properties, and carbon footprint assessment. First, the roles of cement, SCMs, and mineral fillers are reviewed with emphasis placed on how their physicochemical characteristics influence the end product as well as on their sustainability benefits. Second, particle packing design models are examined, and their applicability to UHPC mixture design is evaluated. Third, the influence of raw material characteristics and mixture proportions on key properties (including flowability, mechanical performance, and durability) is synthesized and analyzed. Finally, a database comprising around 200 UHPC mixtures was developed, and correlation heatmap analysis and Random Forest modeling were conducted to capture novel insights into the relationships between mixture composition, mechanical and durability performance, and carbon footprint.

In an attempt to foster comprehensiveness, this review is based on peer-reviewed studies on UHPC materials, mixture design, and sustainability aspects. Emphasis was placed on publications reporting quantitative experimental data and embodied carbon assessments.

## Raw materials

UHPC derives its unique mechanical performance and durability from a carefully engineered combination of cementitious materials and mineral fillers. Unlike conventional concrete, UHPC mixture design is governed by the synergistic interaction between material reactivity, particle size distribution, and packing density. This section examines the key constituents of UHPC with emphasis on their physicochemical characteristics and implications for low-carbon mixture design.

### Cement

Representing the primary binding phase, cement governs early hydration kinetics and contributes significantly to strength development. As it is well established, cement consists mainly of tricalcium silicate (C_3_S, ~ 55%), dicalcium silicate (C_2_S, ~ 20%), tricalcium aluminate (C_3_A, ~ 10%), and tetra calcium alumino-ferrite (C_4_AF, ~ 8%) [16], with CaO and SiO_2_ being the dominant oxides. Compared to the rest of the granular materials in UHPC, cement has a relatively high density (~ 3.15 g/cm^3^). Its mean particle size can vary depending on the cement type and ranges between 10 and 20 µm [16–18].

Various cement types have been reported for use in UHPC mixtures depending on the targeted application requirements and desired rheological and early-age mechanical performance. High early strength (HE) featuring superior fineness and faster hydration is commonly used to enable rapid strength gain [19]. Alternatively, general-use (GU) cement may be used when early strength is less critical [20], while sulfate-resistant (HS) cement is selected for aggressive conditions due to its low C_3_A content, which also fosters controlled rheological properties [21].

Early UHPC formulations used to be cement-intensive, with mixture designs frequently containing 800–1 000 kg/m^3^ of cement [4]. While such high cement contents contribute to compressive strengths exceeding 150 MPa, they also result in significantly high levels of embodied CO_2_. This challenge has prompted the incorporation of SCMs to partially replace cement, while contributing to microstructural refinement and improved mechanical performance and durability.

### SCMs

**Fig. 2:**
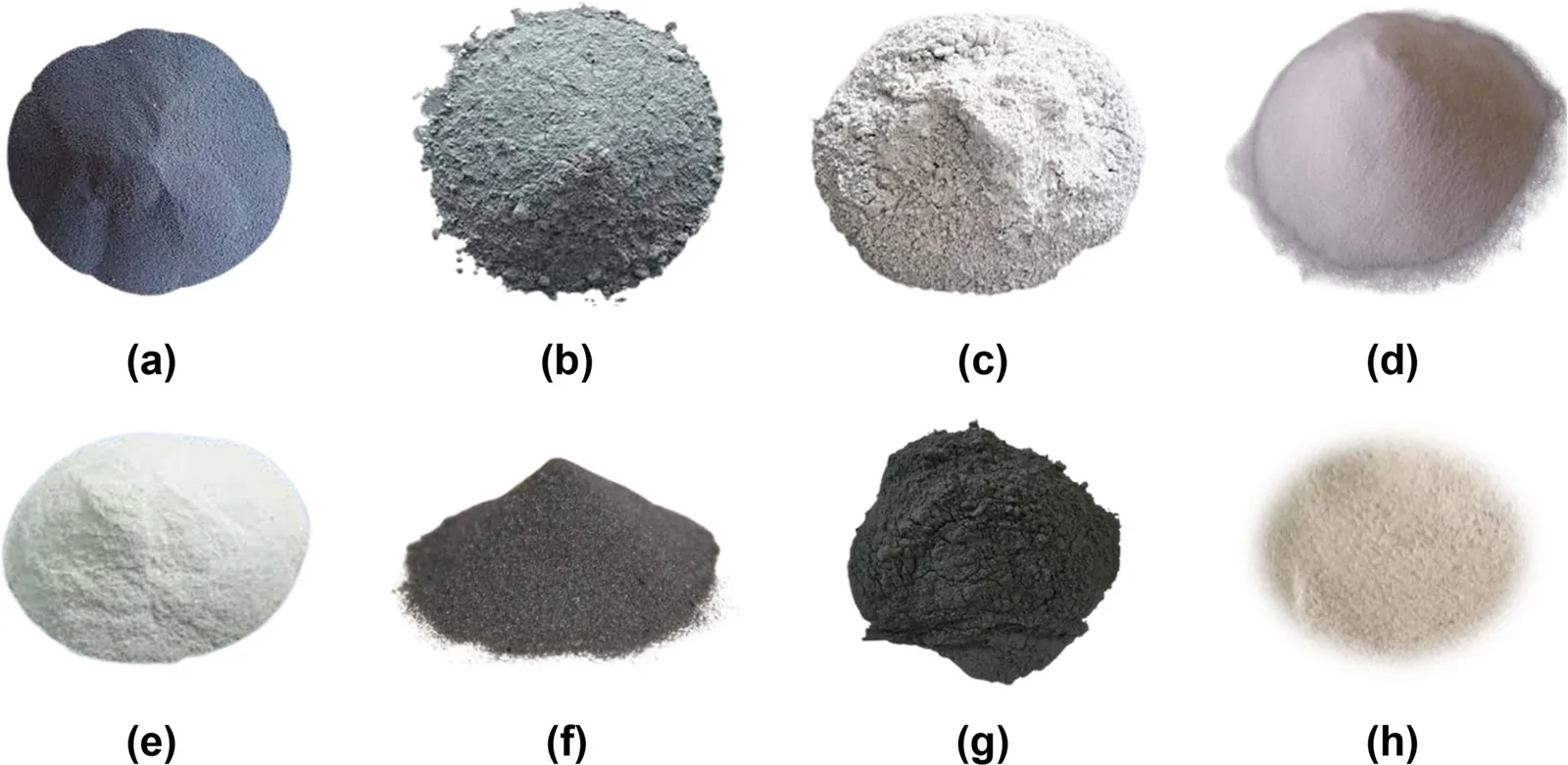
SCMs physical appearance. **a** SF. **b** FA. **c** GGBFS. **d** MK. **e** GGPs. **f** BC. **g** RHA. **h** RCWP

Through a combination of pozzolanic and hydraulic activity, SCMs contribute to the formation of additional calcium silicate hydrate (C–S–H), refine pore structure, and enhance the overall durability by impeding transport of aggressive ions such as chlorides and sulfates. At the same time, their partial replacement of cement reduces clinker content and lowers embodied CO_2_. As illustrated in Fig. 2, a wide range of SCMs has been explored in UHPC formulations. These materials can be broadly categorized into: (i) conventional SCMs, which are well-established in UHPC practice (e.g., SF, FA, ground granulated blast-furnace slag (GGBFS), and MK); and (ii) alternative emerging SCMs (e.g., ground glass pozzolans (GGPs), biochar (BC), rice husk ash (RHA), and recycled coral waste powder (RCWP)).

#### SF

SF is a by-product recovered during the production of alloys containing silicon or elemental silicon [22] and is produced in the high-temperature reduction of quartz in electric arc furnaces, where the primary products are silicon or ferrosilicon alloys [23]. SF is composed predominantly of SiO_2_ (85%–98%) and also contains minor amounts of Al_2_O_3_, Fe_2_O_3_, CaO, MgO, K_2_O, and SO_3_ [22–29]. It has a remarkably high specific surface area (202 000–239 560 cm^2^/g), a density of around 2.3 g/cm^3^, a mean particle size ranging from 0.22 to 0.35 μm, and a specific gravity of around 2.14 [24, 26, 29].

SF is the most widely used SCM in UHPC, with common dosages ranging from 5 to 25% [24–26, 29], due to its ultrafine particle size (submicron scale) and high amorphous SiO_2_ content (around 85%–95%, depending on the source) [30, 31]. Literally, almost all UHPC formulations contain SF for its extremely high specific surface area enabling it to fill nanoscale voids, thereby significantly enhancing packing density. In addition, its high pozzolanic reactivity promotes rapid consumption of calcium hydroxide, leading to the formation of secondary C–S–H and a highly densified microstructure [30]. This combined filler and pozzolanic effect makes SF effective in enhancing compressive strength, densifying interfacial transition zone, and improving the impermeability [32]. However, its high surface area also increases water demand and can adversely affect workability at low water-to-binder ratios, necessitating the use of an increased amount of high-range water-reducing admixtures, which may interfere with the early-age strength development. SF content in UHPC also correlates with autogenous shrinkage triggered by SF’s pore refining effect, which leads to higher surface tension, self-desiccation, and internal volumetric instability. Furthermore, the high cost and limited availability of SF may restrict its use at high replacement levels [30].

#### FA

FA is a byproduct of coal combustion and has two primary classifications, Class F and Class C, with Class F containing less calcium compared to Class C [33]. Depending on the source of coal and combustion conditions, the chemical composition can vary. Typically, Class C has a CaO content (mass fraction) of approximately 13.0%, along with notable amounts of Fe_2_O_3_ (~ 59.0%), Al_2_O_3_ (~ 18.0%), and SiO_2_ (~ 5.0%), while K_2_O, TiO_2_, Mn_2_O_3_, and SO_3_ are generally below 1.0% [33]. In contrast, Class F is predominantly composed of SiO_2_ (44.0%–67.0%), followed by Al_2_O_3_ (22.0%–30.0%), Fe_2_O_3_ (1.0%–14.0%), and CaO (0.4%–7.0%) [33–37]. FA has a density of approximately 2.4 g/cm^3^, a Blaine surface area of around 3 848 cm^2^/g, a specific gravity of 2.38, and a mean particle size (can vary depending on the source) of around 10 μm [36, 37].

The inclusion of FA in UHPC has been reported to impart beneficial effects on both fresh and hardened properties. FA is commonly used to replace cement at levels ranging from 10% to 60% [36, 37]. Its spherical particles improve workability and packing density, while its pozzolanic properties enhance long-term strength and durability through the formation of secondary C–S–H [6]. However, with the global shift toward cleaner energy sources and the gradual phaseout of coal-fired power plants, which is the primary source of FA, the availability of FA has sharply declined in many regions, particularly in North America [38].

#### GGBFS

GGBFS is a byproduct of the steel production industry. It is derived from the blast furnace process, where iron ore, coke, and limestone are melted, forming molten slag. This slag is then separated, rapidly cooled by granulation, and finely ground to produce GGBFS [34].

The physicochemical properties of GGBFS vary depending on the source and processing techniques. It is highly reactive due to its fineness, as it can go up to a specific surface area of 23 100 cm^2^/g [39], with a common range of 4 250–4 700 cm^2^/g [40]. It has a relatively high density of 2.89 g/cm^3^ and a mean particle size ranging from 11 to 32 µm [41–43]. GGBFS is primarily composed of CaO (38%–44%), SiO_2_ (34%–37%), Al_2_O_3_ (10%–13%), and MgO (8%–10%) [41, 42].

GGBFS enhances UHPC performance through its latent hydraulic reactivity, which promotes additional C–S–H formation and improves long-term strength, durability, and chloride resistance [44]. GGBFS can be used as a partial cement replacement at dosages ranging from 30 to 50% [39, 41–43], as it generally improves workability and reduces autogenous shrinkage. However, with high replacement levels or with ultrafine GGBFS, the improvement may diminish or even reduce flowability due to increased surface area and more irregular and rough-textured particles [44]. Its contribution to pore refinement and reduced heat of hydration further supports the production of dense and durable UHPC [44]. From a sustainability perspective, GGBFS's use as a byproduct of the steel industry provides a significant reduction in embodied CO_2_ [44].

#### MK and nano-metakaolin (NMK)

MK is produced by calcining kaolinitic clay, one of the most abundant clays on Earth, at temperatures between 700 ℃ and 850 ℃ [45–47]. A more refined form, NMK, is developed through high-energy milling of raw kaolin [48, 49]. Both forms of MK offer significant pozzolanic activity, making them valuable SCMs in UHPC production. Typically, MK is incorporated as a partial cement replacement at levels ranging from 5% to 20% [45, 46, 50, 51], whereas NMK is primarily used as a nano-additive at around 1% [48, 52].

The physicochemical properties of MK vary depending on the source and processing techniques, but it primarily consists of SiO_2_ (53.00%–74.30%) and Al_2_O_3_ (10.20–43.00%), with minor traces of Fe_2_O_3_ (0.38%–1.82%), CaO (0.40–2.40%), SO_3_ (~ 0.50%), MgO (0–0.33%), and K_2_O (0.10%–4.60%) [46–48, 50, 51, 53]. The composition of NMK is similar but with a significantly higher SiO_2_ content (~ 89.6%), along with Al_2_O_3_ (~ 0.9%), Fe_2_O_3_ (~ 2.0%), CaO (~ 0.4%), MgO (~ 2.0%), and K_2_O (~ 4.6%) [48, 52]. MK has a density of approximately 3.58 g/cm^3^, a mean particle size of around 11 μm, a specific gravity of 2.50–2.57, and a specific surface area of roughly 200 000 cm^2^/g [45, 46, 50, 51].

#### GGPs

GGPs are produced by grinding waste glass into fine powder, which can be processed to micro and nano levels to be used as a partial cement replacement, SF, or quartz sand [54, 55]. Improper disposal of waste glass remains an environmental concern, as glass does not naturally decompose. In this context, GGP offers a sustainable alternative for waste management while serving as a valuable SCM [56].

The physicochemical properties of GGP vary depending on the source and processing techniques, but it is generally composed of SiO_2_ (66.30%–89.40%), CaO (3.20%–12.20%), and Na_2_O (0.40%–14.90%), with traces of Al_2_O_3_ (0.20%–3.70%), Fe_2_O_3_ (0.04%–1.80%), SO_3_ (0.02%–0.30%), MgO (0.50%–2.90%), TiO_2_ (0–0.04%), and K_2_O (0.01%–0.70%) [54–63]. The specific gravity of GGP ranges from 2.52 to 2.63 [54, 56, 58, 60, 62]. Its specific surface area varies significantly based on grinding levels, reaching up to 130 500 cm^2^/g at the nanoscale and between 3 300 and 5 885 cm^2^/g at the micro-scale. The mean particle size of GGP ranges from 316 nm (0.316 µm), associated with nanoscale GGP used as a slurry, to 17 µm. This reflects the significant differences in processing intensity and application requirements [54–56, 58, 60].

Due to its amorphous structure and high silica content [64], GGP exhibits high pozzolanic activity, making it a viable partial cement replacement. Depending on the particle size, GGP can be used to replace cement, SF, or quartz sand at levels ranging from 10% to 50% [54–56, 58, 60]. Its incorporation in UHPC improves packing density, reduces porosity, and contributes to long-term strength and durability [65]. However, the relatively high Na_2_O content in some glass sources has raised concerns about potential alkali-silica reaction (ASR) [63]. However, recent studies have shown that when waste glass is finely ground (typically < 300 μm), it does not promote ASR and can even mitigate expansion by reacting with calcium hydroxide to form additional C–S–H gel, thereby lowering pore solution alkalinity and enhancing durability [66].

#### BC

BC is a highly carbonaceous by-product produced by the pyrolysis of biomass, which includes renewable plant-based materials such as wood and crop residues, under oxygen-limited conditions at 300–550 ℃. The decomposition of cellulose, hemicellulose, and lignin during this process yields a stable, porous material with a high carbon content [67–70]. In addition to its carbon sequestration potential, which can offset up to 870 kg of CO_2_-equivalent per ton of biomass [67], BC provides valuable benefits for cementitious composites, including improved water retention and internal curing [67, 69].

The composition (mass fraction) of BC varies based on feedstock and production conditions, typically containing 50.0%–93.0% carbon [71], along with oxygen (16.0%–26.0%), hydrogen (~ 2.5%), nitrogen (~ 1.2%), and trace amounts (< 0.5%) of silica, calcium, aluminum, magnesium, and potassium [67, 72, 73]. Its particle size can range from 10 to 270 µm, with finer particles resulting from intensive grinding and milling [72, 74]. The porous nature of BC, which is formed during the decomposition of organic substances, contributes to its ability to enhance the hydration process. Its density varies significantly, ranging from 0.001 61 to 1.560 00 g/cm^3^, depending on its processing and feedstock characteristics [67, 69].

In UHPC, BC is typically used as a cement replacement at relatively low dosages (0.25%–2.00%), primarily due to its high absorption capacity and influence on rheological behavior [67, 69, 72, 74]. In addition to enhancing internal curing, BC contributes to improved UHPC performance by promoting hydration and refining the microstructure, which reduces porosity and mitigates autogenous shrinkage [69]. Its high carbon content and porous structure also improve UHPC's workability and reduce the mixture’s carbon footprint [69].

#### RHA

Rice husk is the protective outer layer of the rice kernel and is an abundant agricultural byproduct, with approximately 200 kg of rice husk generated per ton of rice. Upon combustion, risk husk yields nearly 18%–20% RHA [75, 76]. With global rice production reaching 723 million tons in 2011, over 145 million tons of husks were generated, most of which were discarded, exacerbating solid waste stock [75]. Due to its unique composition, RHA has great potential to serve as an SCM and partially replace cement in UHPC.

Depending on the production conditions, RHA can have a specific gravity of around 2.16–2.28, a mean particle size of approximately 7.5 µm, and a specific surface area of around 15 991 cm^2^/g [77, 78]. Chemically, it is primarily composed of SiO_2_ (85%–95%), K_2_O (2%–6%) and traces of Al_2_O_3_, Fe_2_O_3_, and CaO [75–82]. Its high silica content enables it to promote the hydration process through high pozzolanic reactions, which improve the performance of the cementitious system.

In UHPC, RHA enhances mechanical strength and durability by promoting additional C–S–H formation and refining the microstructure [83]. Its fine particles improve packing density and contribute to pore refinement, while partial cement replacement reduces the mixture’s carbon footprint [83]. RHA has been used as a partial cement replacement at levels of 10%–50%, while its complete replacement of SF has also been reported in some UHPC formulations [77, 78].

#### RCWP

RCWP is a byproduct of marine construction activities and is abundant in tropical coastal regions. Composed primarily of aragonite, calcite, dolomite, and high-Mg calcite, coral has a naturally porous and absorbent structure [84, 85]. Large quantities of coral waste are often landfilled, posing great ecological risks, but its reuse as an SCM offers an environmentally responsible alternative, reducing cement demand and mitigating impacts from marine construction activities.

Chemically, RCWP is close to cement, with Ca (47.70%–52.70%) as the dominant component, along with minor amounts of SiO_2_ (0.40%–1.70%), Al_2_O_3_ (0.10%–0.40%), Fe_2_O_3_ (0.04%–0.40%), SO_3_ (0.60%–0.70%), Na_2_O (0.60%–2.00%), MgO (0.20%–2.50%), and K_2_O (0–0.10%) [84–87]. Approximately, it has a mean particle size of around 9.0 μm and a specific gravity of around 2.48.

In UHPC, RCWP has been used as a partial replacement of cement, SF, and quartz sand, with reported replacement levels ranging from 5% to 60% for cement, 15%–45% for SF, and 10%–30% for quartz sand [87]. Due to its composition and fine particle size, RCWP improves UHPC's performance by serving as a filler and also participating in the secondary hydration process.

#### Role in UHPC design

The aforementioned SCMs contribute differently to UHPC performance depending on their reactivity, particle morphology, and influence on particle packing and pore refinement. Ultrafine materials such as SF primarily densify the matrix, enhance the packing density, and improve early-age strength through both filler and pozzolanic effects, in addition to serving as nucleation sites. Moderately reactive SCMs such as FA and GGBFS contribute to long-term hydration, enhanced durability, and improved workability. Meanwhile, materials such as GGP, RHA, and BC have demonstrated great potential for producing LC-UHPC due to their favorable pozzolanic activity, microstructural refinement, and reduced carbon footprint.

From a mixture design perspective, the effectiveness of SCMs extends beyond their chemical reactivity. Their particle size distribution and morphology directly influence packing density, which impacts the porosity and mechanical performance. Consequently, the selection and proportioning of SCMs must be considered within a particle packing framework, where multi-scale particle distributions are optimized to achieve maximum density and efficiency. From the carbon footprint side, SCMs incorporation is the main approach to reduce the clinker content and the elevated CO_2_ emissions in UHPC. However, achieving LC-UHPC requires balancing cement reduction with performance requirements. Table 1 summarizes the key physical properties of the SCMs discussed, including specific gravity, particle size, and surface area, providing a comparative basis for their selection and optimization in UHPC mixture design. These properties are particularly critical for evaluating their contribution to particle packing density and overall material efficiency.

**Table 1:** Summary of SCMs’ physical properties

Material	Specific gravity	Mean particle size (µm)	Surface area (cm^2^/g)	Material replaced	Typical replacement	Refs.
**SF**	2.14–2.30	0.22–0.35	202 000–239 560	C	5% to 25%	[24–26, 29]
**FA**	2.38	9.80	3 848	C	10% to 70%	[36, 37]
**GGBFS**	2.89	11.00–32.00	23 100	C	30% to 50%	[39, 41–43]
**MK**	2.50–2.57	11.00	200 000	C	5% to 20%	[45, 46, 50, 51]
**NMK**	-	-	-	-	Additive—1%	[48, 52]
**GGP**	2.52–2.63	0.32–17.00	3 300–130 500	C, SF, and QS	10% to 50%	[54–56, 58, 60]
**BC**	0.001 61–1.560 00	10.00–270.00	-	C	0.25% to 2.00%	[67, 69, 72, 74]
**RHA**	2.16–2.28	7.50	15 991	C and SF	C: 10%–50%, SF: up to 100%	[77, 78]
**RCWP**	2.48	9.00	-	C, SF, and QS	C: 5% to 60%, QS: 10% to 30%, SF: 15% to 45%	[87]

*C* cement, *SF* silica fume, *QS* quartz sand

However, reported optimal replacement levels for the same SCM vary across studies, reflecting differences in cement types, water-to-binder ratios, and curing regimes, which complicates direct comparison between sources. Furthermore, emerging SCMs such as BC, RHA, and RCWP are characterized far less consistently than conventional SCMs, with variable reporting of particle size distribution and chemical composition across studies. Most existing research also evaluates SCMs individually, and the synergistic effects of combining fast- and slow-reacting SCMs within a single mixture remain comparatively underexplored, representing a gap that limits the optimization of multi-SCM UHPC systems.

### Mineral fillers

Unlike SCMs, which contribute chemically through pozzolanic reactions, mineral fillers are physically active components through filler and nucleation effects. Their incorporation enables the reduction of voids across multiple length scales, thereby improving mechanical performance and durability.

**Fig. 3:**
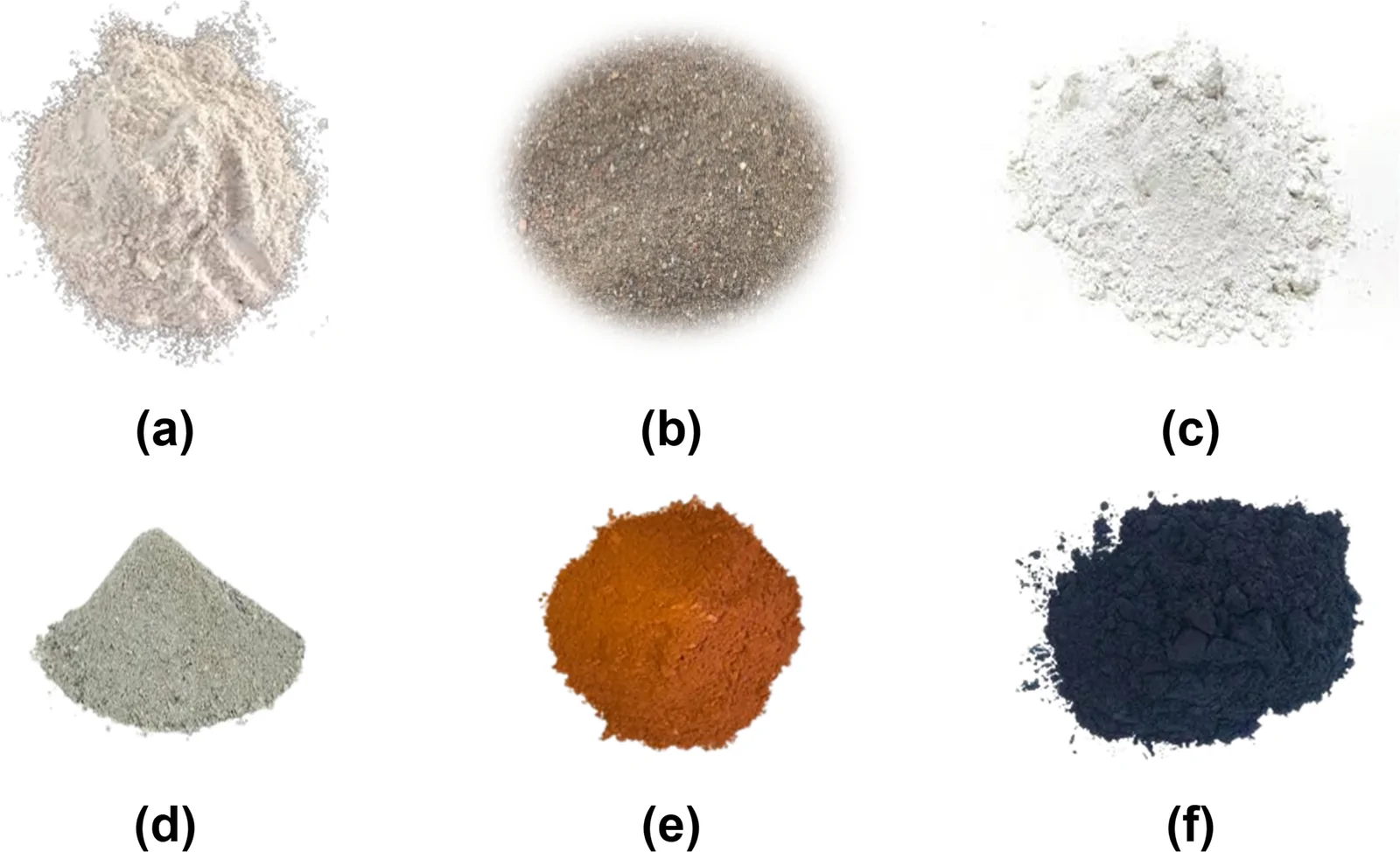
Physical appearance of mineral fillers. **a** LP. **b** RCFs. **c** CWP. **d** GRP. **e** RM. **f** IOTs

As illustrated in Fig. 3, a wide range of mineral fillers has been investigated for UHPC applications, including limestone powder (LP), recycled concrete fines (RCFs), ceramic waste powder (CWP), granite powder (GRP), red mud (RM), and iron ore tailings (IOTs). These materials vary significantly in terms of particle size, morphology, and chemical composition, which in turn control their effectiveness in improving the packing density and hydration reactions. Mineral fillers in UHPC can be broadly classified into: (i) inert or weakly reactive fillers, which primarily improve packing density (e.g., LP, GRP); and (ii) partially reactive fillers, which combine filler effects with limited pozzolanic or hydraulic activity (e.g., RCFs, CWP, RM).

#### LP

LP is produced by crushing and grinding natural limestone, which mainly consists of skeletal fragments of organisms and mineral phases. Limestone forms in diverse environments, including marine, lacustrine, and evaporitic settings, or through chemical precipitation of calcite or aragonite. Its structure varies from crystalline and clastic to granular, depending on its formation process [88]. LP is one of the most widely used fillers in UHPC due to its availability, low cost, and favorable particle size. It is commonly used as a partial replacement of cement in the range of 10%, and it can reach an extreme level of 80% (accompanied by a drastic drop in compressive strength) [41, 88, 89]. Its relatively fine particles enable efficient filling of interstitial voids between cement and SCM particles, thereby enhancing packing density and reducing porosity. Although largely inert, limestone can participate in limited chemical interactions.

The physicochemical properties of LP vary depending on the source and grinding levels. In general, its physical properties include a density of 2.68–2.72 g/cm^3^ [41, 89], and a mean particle size between 0.70 and 20.00 µm [88]. Chemically, LP is dominated by CaO, exceeding 80.0% of its composition, along with traces of SiO_2_ (2.0%–4.4%) and Fe_2_O_3_ (0.8%–1.6%). In cases with higher impurity levels, the mass fraction of CaO may decrease to about 50.0% [41, 90].

#### RCFs

RCFs are obtained from the crushing and screening of concrete debris generated during construction, maintenance, or demolition of concrete structures [91, 92]. The construction sector is among the world’s largest waste producers; in Canada alone, over 4 million tons of construction materials were landfilled in 2020, accounting for approximately 1.8 million tons of embodied carbon [93]. Advances in crushing and sorting technologies have enhanced the recycling potential of this waste, enabling the production of fine recycled aggregates and promoting more sustainable construction materials [94].

RCFs primarily consist of SiO_2_ (~ 41.1%) and CaO (~ 33.4%), with smaller amounts of Al_2_O_3_ (~ 11.8%) and Fe_2_O_3_ (~ 4.0%) [95]. Particle sizes of RCFs typically range from 0.063 to 2.360 mm, depending on the process and degree of grinding [91, 95, 96]. RCFs exhibit considerable variation in density, with a bulk density around 1.37 g/cm^3^ and apparent density ranging from 2.28 to 2.33 g/cm^3^ [91, 97]. This variation is due to differences in sources and compositions of the demolished concrete.

The high CaO and SiO_2_ contents govern RCF’s reactivity and make it suitable for incorporation in UHPC. Depending on the application and particle size of RCF, it can be used as a partial replacement for quartz sand, natural sand, mineral powders, and basalt aggregates. Reported replacement levels include approximately 11%–33% for mineral powder, 20%–100% for natural sand, 10%–30% for quartz sand, and 50%–100% for basalt aggregate [91, 95–97]. RCFs enhance particle packing and contributes to microstructural densification [98]. Its residual cementitious phases can participate in secondary hydration, improving strength and durability while reducing clinker demand. Thus, integrating RCFs into UHPC not only diverts construction waste away from landfills but also supports a lower-carbon and more circular construction industry.

#### CWP

CWP is obtained by crushing and grinding waste from the manufacturing and disposal of ceramic products. The high-temperature processing of ceramics (200–2 000 ℃) imparts pozzolanic reactivity, contributing to long-term strength and durability [99, 100]. While primarily used as a filler in concrete, CWP’s pozzolanic activity increases with finer particle sizes and higher specific surface area, enhancing hydration at later stages [101].

CWP is mainly composed of SiO_2_ (61.70%–78.30%) and Al_2_O_3_ (15.90%–22.30%), with minor amounts of CaO (0.90%–6.67%), Fe_2_O_3_ (1.10%–4.32%), MgO (0.70%–3.50%), K_2_O (1.60%–2.10%), and Na_2_O (1.00%–3.12%) [60, 100, 102–105]. Its specific gravity ranges from 2.40 to 2.61, water absorption rate of 2% [106], and its specific density ranges from 2.43 to 2.56 g/cm^3^ [102, 104]. Its specific surface area is between 4 580 and 5 790 cm^2^/g [60, 102], and the mean particle size is approximately 3.5 µm [102], but it can vary depending on the grinding and screening level.

In UHPC, CWP can partially replace cement or quartz sand, typically at levels of 10%–55% and 50%–100%, respectively [60, 102, 104, 106]. It can enhance particle packing and densify the microstructure [107]. Its semi-pozzolanic properties contribute to additional C–S–H formation, improving long-term strength and durability [107]. By utilizing CWP as a supplementary material, UHPC mixtures benefit from reduced clinker content and a lower carbon footprint, without compromising mechanical performance.

#### GRP

GRP is an industrial byproduct from cutting granite blocks or grinding raw granite materials [108]. While primarily used as a filler in concrete, GRP exhibits pozzolanic activity, which increases with finer particle sizes due to the larger surface area available for reaction with calcium hydroxide. This enhances long-term concrete performance by promoting additional hydration products.

GRP consists mainly of SiO_2_ (70.0%–83.0%), Al_2_O_3_ (2.0%–15.0%), and Fe_2_O_3_ (1.0%–14.8%). It has small amounts of CaO (0.4%–2.6%), MgO (0.2%–0.9%), SO_3_ (0.1%–0.3%), K_2_O (4.6%–5.8%), and Na_2_O (2.9%–3.9%). It has an approximate specific gravity of around 2.68 and a surface area of around 5 735 cm^2^/g (depending on the level of grinding) [60, 108–111].

In UHPC, GRP is used as a partial replacement for cement and quartz sand at replacement levels of approximately 20%–26% and 35%–100%, respectively. Due to its unique composition, GRP primarily contributes through filler and packing effects, improving matrix compactness and reducing void content, which supports enhanced mechanical performance and durability. It can also improve secondary hydration due to its rich composition of SiO_2_ [60, 89, 108].

#### RM

RM is a highly alkaline byproduct generated during alumina production, with 1 to 1.5 tons of RM produced per ton of alumina [112–114]. Its large volume and chemical reactivity have drawn attention for sustainable construction applications, particularly as a partial cement replacement due to its pozzolanic activity [90].

The chemical composition of RM varies with source but is dominated by Fe_2_O_3_ (30.0%–50.0%), Al_2_O_3_ (18.0%–22.0%), and SiO_2_ (6.0%–20.0%). It also contains CaO (4.5%–13.0%), Na_2_O (6.0%–13.6%), TiO_2_ (3.9%–5.2%), and trace amounts of MgO, K_2_O, and SO_3_ (< 1%) [90, 112, 114]. In some cases, the mass fraction of Al_2_O_3_ may exceed that of Fe_2_O_3_, reaching up to 41.0% [113, 115]. RM has a specific density of approximately 2.97 g/cm^3^, a surface area of around 1 268 000 cm^2^/g (depending on the source) [113], and a mean particle size of around 5.47 µm [114], with its fine particles and high surface area enhancing its reactivity.

In UHPC, RM is commonly incorporated as a partial cement replacement at levels ranging from 2.5% to 60% [113, 114]. Its fine particle size and high surface area enhance particle packing and microstructural densification [112]. Its pozzolanic activity contributes to additional C–S–H formation, improving long-term strength and durability [112]. Moreover, the high iron content of RM makes it an attractive option for special types of concrete, especially developing radiation-shielding UHPC.

#### IOTs

IOTs are mineral waste byproducts generated during the beneficiation process of iron ore. These tailings are typically stored in stacks, with limited utilization options [116]. In China alone, nearly 600 million tons are produced annually, representing over 50% of the global generation [117].

IOT properties might change depending on the source, but it is primarily composed of SiO_2_ (52.00%–75.00%), Fe_2_O_3_ (6.00%–9.00%), Al_2_O_3_ (3.50%–17.00%), CaO (4.00%–12.00%), SO_3_ (0.05%–10.60%), and MgO (3.00%–6.00%) [117–122]. It has a surface area ranging from 439 to 1 185 cm^2^/g, a mean particle size of 166.2 µm [117, 120], and a density between 2.62 and 2.70 g/cm^3^ [117, 121, 122].

In UHPC, IOT can partially replace cement and fine aggregates. Reported replacement levels range from 10% to 30% for cement and 20%–100% for quartz sand [117, 120–122]. While IOT exhibits low early-age pozzolanic reactivity, its fine particles act as nucleation sites and contribute a filling effect that densifies the microstructure, which supports long-term strength development and improved durability [116].

#### Role in UHPC design

Mineral fillers are essential for achieving the dense particle packing that drives the great strength of UHPC. By filling voids across multiple size scales, they reduce porosity, enhance strength, and improve durability, while for some fillers, also enabling reductions in binder content.

From a particle packing perspective, mineral fillers complement SCMs by occupying intermediate and fine particle size ranges, thereby facilitating continuous particle size distributions. This multi-scale packing approach is crucial for minimizing voids and optimizing material usage. From a sustainability standpoint, mineral fillers contribute to LC-UHPC by possibly reducing cement and valorizing industrial waste. However, their effectiveness depends on achieving an optimal balance between packing efficiency and reactivity. Table 2 summarizes the key physical properties of the mineral fillers discussed, providing a comparative basis for their selection and integration into UHPC mixture design.

**Table 2:** Summary of mineral fillers’ physical properties

Material	Specific gravity	Mean particle size (µm)	Surface area (cm^2^/g)	Material replaced	Typical replacement	Refs.
**LP**	2.68–2.72	0.70–20.00	-	C	10% to 80%	[41, 88, 89]
**RCF**	1.37–2.33	63.00–2 360.00	-	MP, NS, QS, and BA	MP: 11% to 33%, NS: 20% to 100%, QS: 10% to 30%, BA: 50% to 100%	[91, 95–97]
**CWP**	2.40–2.61	3.50	4 580–5 790	C and QS	C: 10% to 55%, QS: 50% to 100%	[60, 102, 104, 106]
**GRP**	2.68	-	5 735	C and QS	C: 20% to 26%, QS: 35% to 100%	[60, 89, 108]
**RM**	2.97	5.47	1 268 000	C	2.5% to 60%	[113, 114]
**IOT**	2.62–2.70	166.20	439–1 185	C and QS	C: 10% to 30%, QS: 20% to 100%	[117, 120–122]

*C* cement, *SF* silica fume, *QS* quartz sand, *MP* mineral powder, *NS* natural sand, *BA* basalt aggregate

Some fillers, such as GRP and CWP, exhibit semi-pozzolanic behavior, which obscures the conventional distinction between inert fillers and reactive SCMs and results in inconsistent classification across the literature. A further limitation is that many waste-derived fillers, including RCF, RM, and IOT, show considerable compositional variability depending on their source and processing history, which limits the generalizability of reported optimal replacement levels across studies and hinders their widespread use in standardized mixture design guidelines.

## PPDMs

**Fig. 4:**
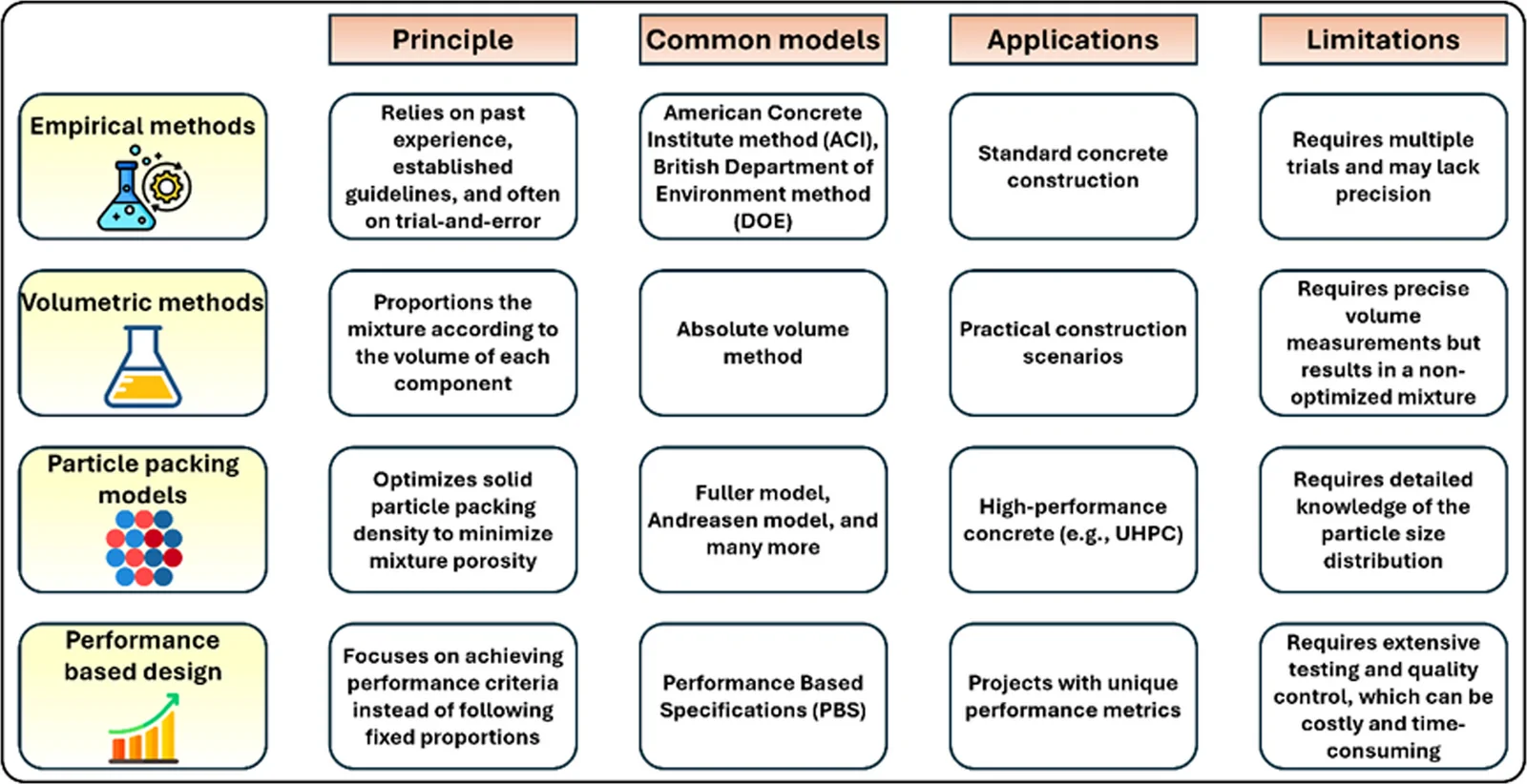
Concrete mixture design approaches

Traditional concrete mixture design approaches (Fig. 4) are largely empirical and were developed for conventional concrete systems with relatively coarse particle distributions. In contrast, UHPC relies on densely packed, multi-scale granular systems, where performance is governed by particle size distribution (PSD), packing density, and interparticle interactions. As such, PPDMs have become essential tools for UHPC mixture optimization, enabling the reduction of voids, enhancement of mechanical performance, and improvement of durability [123].

Packing density (${\varnothing}_{\mathrm{P}}$), defined as the ratio of solid volume (${\mathrm{V}}_{\mathrm{s}\mathrm{o}\mathrm{l}\mathrm{i}\mathrm{d}\mathrm{s}}$) to total volume (${\mathrm{V}}_{\mathrm{t}\mathrm{o}\mathrm{t}\mathrm{a}\mathrm{l}}$), represents a fundamental parameter governing UHPC performance (Eq. (1)) [124]. Optimized packing density reduces porosity, lowers water demand, and enables more efficient use of cementitious materials, an aspect that is directly relevant to the development of LC-UHPC.

1 $${\varnothing}_{\tt{P}}=\frac{{\mathrm{V}}_{\mathrm{s}\mathrm{o}\mathrm{l}\mathrm{i}\mathrm{d}\mathrm{s}}}{{\mathrm{V}}_{\mathrm{t}\mathrm{o}\mathrm{t}\mathrm{a}\mathrm{l}}}$$

PPDMs are classified into discrete and continuous models. Discrete models describe particle interactions between individual size classes, whereas continuous models represent the whole spectrum of particle sizes using mathematical functions. While both approaches provide insight into packing behavior, their applicability to UHPC differs significantly.

### Discrete models

Discrete packing models, including those proposed by Furnas, Toufar, and De Larrard, are based on the interaction between particles of different sizes. These models provide an understanding of the packing phenomena, particularly the role of fine particles in filling voids between coarser particles.

#### Furnas model

**Fig. 5:**
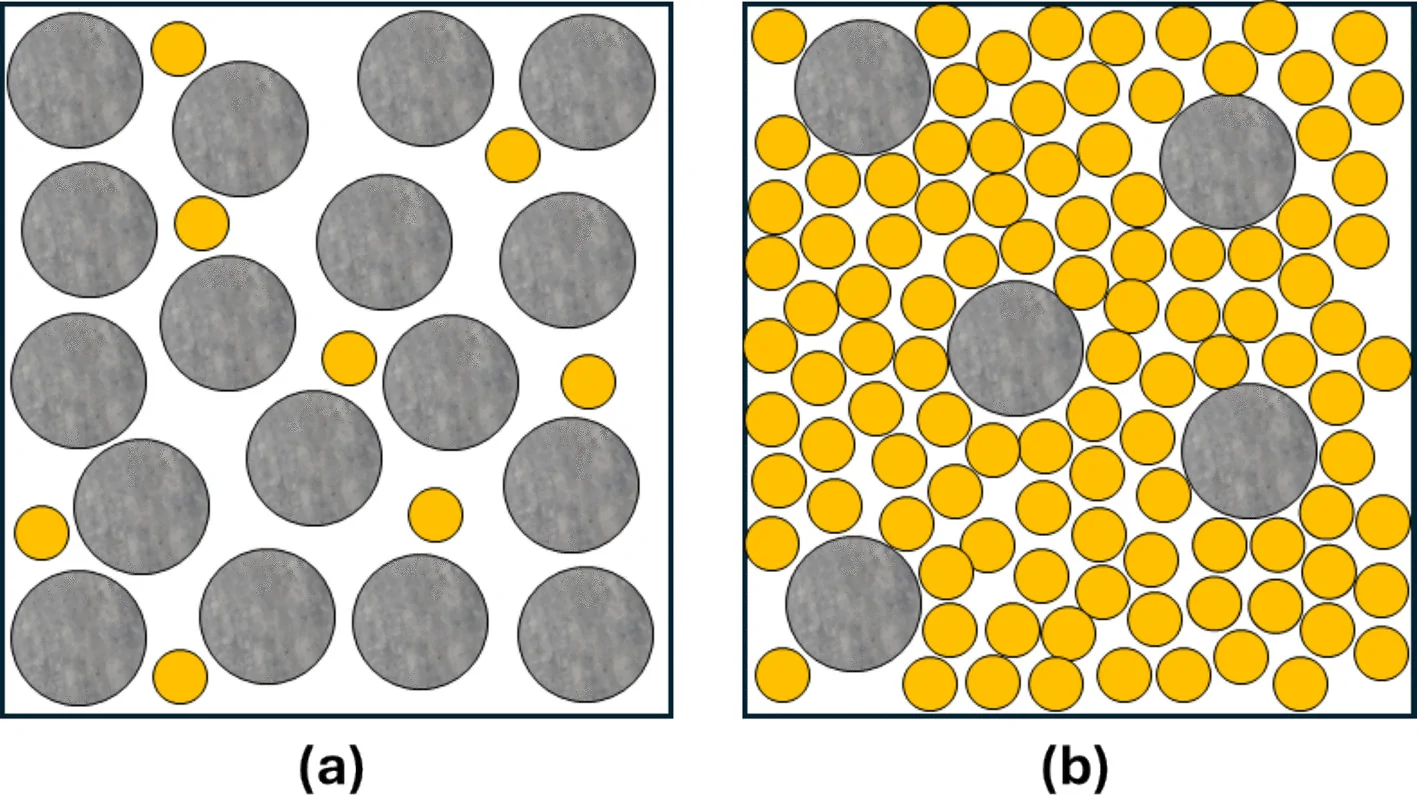
Binary systems. **a** Coarse particles are dominant. **b** Fine particles are dominant

The Furnas model is one of the earliest binary packing models, describing systems composed of coarse and fine particles [125, 126]. It is based on the principle that fine particles can fill the voids between coarse particles, thereby increasing the overall packing density. Two main scenarios are considered in this model (Fig. 5): (I) the volume fraction of the fine particles (${V}_{\mathrm{F}\mathrm{F}}$) is much lower than the volume fraction of the coarse particles (${V}_{\mathrm{F}\mathrm{C}}$), and (II) the dominant volume fraction is the fine particle fraction (${V}_{\mathrm{F}\mathrm{F}}$) [124, 127].

In scenario I, the partial volume of the coarse particles (${V}_{\mathrm{C}}$) corresponds to the residual packing density of the coarse particles (${\varnothing}_{\mathrm{P}\mathrm{C}}$) because the coarse particles rearrange to achieve their maximum packing state, creating voids that can accommodate the fine particles without displacing the coarse particles. Therefore, the packing density of the system (${\varnothing}_{\mathrm{P}1}$) is the sum of the maximum packing density of the coarse particles (${\varnothing}_{\mathrm{P}\mathrm{C}}$) and the partial volume of the fine particles (${V}_{\mathrm{F}}$), as shown in Eq. (2) [124, 127].

2 $${\varnothing}_{\mathrm{P}1}={V}_{\mathrm{C}}+{V}_{\mathrm{F}}={\varnothing}_{\mathrm{P}\mathrm{C}}+{V}_{\mathrm{F}}=\frac{{\varnothing}_{\mathrm{P}\mathrm{C}}}{1-{V}_{\mathrm{F}\mathrm{F}}}=\frac{{\varnothing}_{\mathrm{P}\mathrm{C}}}{{V}_{\mathrm{F}\mathrm{C}}}$$

In scenario II, coarser particles are added to a system where fine particles are dominant. This means that the partial volume of the coarse particles (${V}_{\mathrm{C}}$) will equal their impact on the system’s packing density (${\varnothing}_{\mathrm{P}2}$). The fine particles will fill the remaining volume with their maximum packing density (${\varnothing}_{\mathrm{P}\mathrm{F}}$), as illustrated in Eq. (3) [124, 127].

3 $${\varnothing}_{\mathrm{P}2}={V}_{\mathrm{C}}+{V}_{\mathrm{F}}={V}_{\mathrm{C}}+{\varnothing}_{\mathrm{P}\mathrm{F}}(1-{V}_{\mathrm{C}})=\frac{1}{{V}_{\mathrm{F}\mathrm{C}}+\frac{{V}_{\mathrm{F}\mathrm{F}}}{{\varnothing}_{\mathrm{P}\mathrm{F}}}}$$

**Fig. 6:**
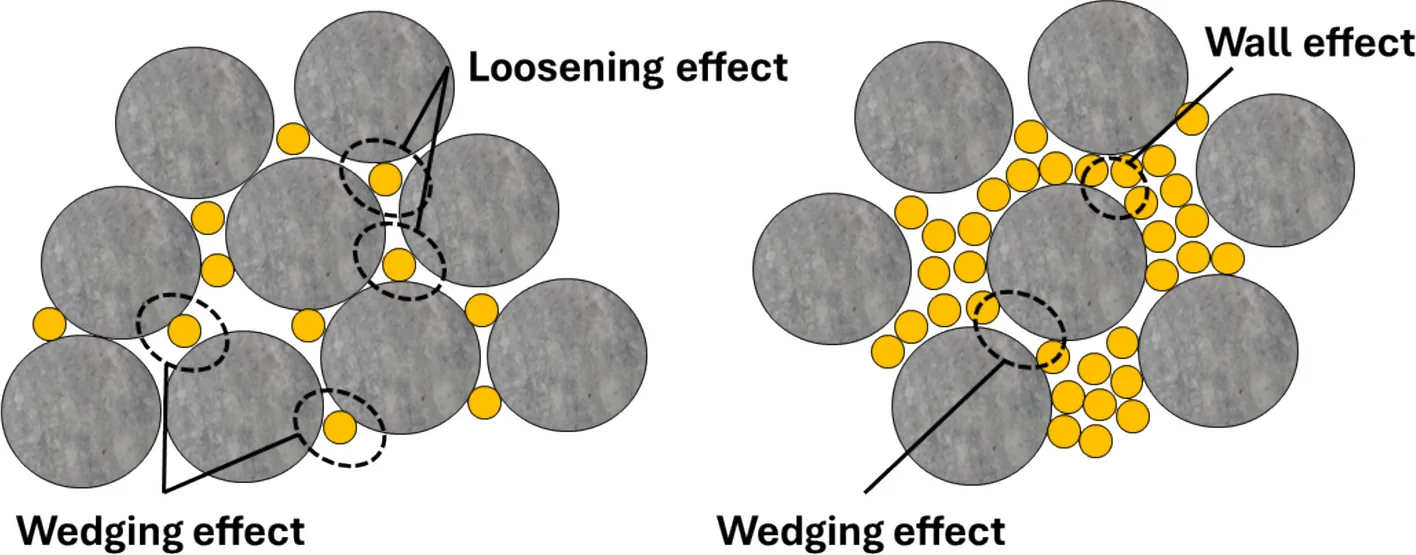
Mechanisms influencing particle arrangement: wall effect, wedging effect, and loosening effect

While the model provides a useful conceptual framework, it requires a significant size difference between particle classes to achieve optimal packing. When this condition is not satisfied, several phenomena, namely the loosening effect, wall effect, and wedging effect (Fig. 6) [124], can reduce packing efficiency. These effects are particularly relevant in UHPC, where particle sizes often overlap across multiple scales.

The loosening effect occurs when fine particles are larger than the voids between coarse particles, thus disturbing and displacing the coarse particles, increasing the system’s porosity. The wall effect prevails when fine particles are trapped between the coarse particles, leading to increased porosity. The wedging effect occurs when fine particles are wedged between coarse particles, increasing the matrix porosity.

Furnas further extended the model to multi-modal cases (Eq. (4)). In this context, monodisperse materials with a discrete distribution represent only a fraction of the multi-modal system, which forms a geometric progression similar to a continuous distribution encompassing all particle sizes [124].

4 $$\mathrm{C}\mathrm{P}\mathrm{F}\mathrm{T}=\left(\frac{{D}_{\mathrm{P}}^{\mathrm{log}r}-{D}_{\mathrm{S}}^{\mathrm{log}r}}{{D}_{\mathrm{L}}^{\mathrm{log}r}-{D}_{\mathrm{S}}^{\mathrm{log}r}}\right)\times 100\mathrm{\%}$$

where $\mathrm{C}\mathrm{P}\mathrm{F}\mathrm{T}$ is the cumulative percentage finer than the particle diameter (${D}_{\mathrm{P}}$); ${D}_{\mathrm{S}}$ is the smallest particle diameter in the system; ${D}_{\mathrm{L}}$ is the largest particle diameter in the system; and r is the ratio between the retained volume of particle diameter (${D}_{\mathrm{P}}$) and the next smaller sieve (${D}_{\mathrm{P}-1}$).

Although the Furnas model can be extended to multi-component systems, its discrete nature limits its applicability to complex UHPC mixtures with continuous PSDs.

#### Toufar model

The Toufar model [128] extends the Furnas approach by accounting for the loosening and wall effects when the fine (${D}_{\mathrm{F}}$) and coarse (${D}_{\mathrm{C}}$) particles are close in size. The model calculates the packing density for mixtures with diameter ratios of $0.22 <{D}_{\mathrm{F}}/{D}_{\mathrm{C}}<1$ [124]. For diameter ratios larger than 0.22, the smaller particles (${D}_{\mathrm{F}}$) are too large to fit within the interstices between the large particles (${D}_{\mathrm{C}}$). Thus, the packing density depends on the ratio between the two diameters (${D}_{\mathrm{F}}$ and ${D}_{\mathrm{C}}$), represented by the factor ${K}_{\mathrm{D}}$ [127]. This makes it more suitable for systems where fine particles cannot fully occupy the voids between coarse particles.

Additionally, the model considers the statistical probability of interstices between the coarse particles that remain unoccupied by fine particles, assuming each fine particle is placed between four coarse particles, leading to the formation of factor ${K}_{\mathrm{S}}$. The packing density as per the Toufar model is shown in Eqs. (5, 6, 7 and 8) [127, 129].

5 $${\varnothing}_{\mathrm{P}}=\frac{1}{\frac{{V}_{\mathrm{F}\mathrm{C}}}{{\varnothing}_{\mathrm{P}1}}+\frac{{V}_{\mathrm{F}\mathrm{F}}}{{\varnothing}_{\mathrm{P}2}}-{V}_{\mathrm{F}\mathrm{F}}\left(\frac{1}{{\varnothing}_{\mathrm{P}2}}-1\right){K}_{\mathrm{D}}{K}_{\mathrm{S}}}$$

6 $${K}_{\mathrm{D}}=\left(\frac{{D}_{\mathrm{C}}-{D}_{\mathrm{F}}}{{D}_{\mathrm{F}}+{D}_{\mathrm{C}}}\right)$$

7 $${K}_{\mathrm{S}}=1-\frac{1+4X}{{\left(1+X\right)}^{4}}$$

8 $$X=\frac{{V}_{\mathrm{F}\mathrm{C}}}{{V}_{\mathrm{F}\mathrm{F}}}\cdot \frac{{\varnothing}_{\mathrm{P}2}}{{\varnothing}_{\mathrm{P}1}\left(1-{\varnothing}_{\mathrm{P}2}\right)}$$

Further examination of the Toufar model revealed that the packing density of a sample of coarse particles does not increase when a small amount of fine particles is added. This issue arises from the assumption that each fine particle is placed between four coarse particles. To address this, modifications were made to the ${K}_{\mathrm{S}}$ factor, as shown in Eqs. (9) and (10) [127].

9 $${K}_{\mathrm{S}}=\frac{0.388 1X}{0.475 3}, \mathrm{when}\ X < 0.475 3$$

10 $${K}_{\mathrm{S}}=1-\frac{1+4X}{{\left(1+X\right)}^{4}}, \mathrm{when}\ X \ge 0.4753$$

The Toufar model can also be applied to estimate the packing density of multi-component systems, as demonstrated in Eqs. (11, 12, 13 and 14) [127].

11 $${V}_{\mathrm{F} i-j}=\frac{{V}_{\mathrm{F}i}\cdot {V}_{\mathrm{F}j}}{1-{V}_{\mathrm{F}i}}$$

12 $${V}_{\mathrm{F} ij}={V}_{\mathrm{F} i-j}+{V}_{\mathrm{F} j-i}$$

13 $${\varnothing}_{\mathrm{P}ij}=\frac{1}{\frac{\left(\frac{{V}_{\mathrm{F} i-j}}{{V}_{\mathrm{F} ij}}\right)}{{\varnothing}_{\mathrm{P}i}}+\frac{\left(\frac{{V}_{\mathrm{F} j-1}}{{V}_{\mathrm{F} ij}}\right)}{{\varnothing}_{\mathrm{P}j}}-\left(\frac{{V}_{\mathrm{F} j-1}}{{V}_{\mathrm{F} ij}}\right)\cdot \left(\frac{1}{{\varnothing}_{\mathrm{P}j}}-1\right)\cdot {K}_{\mathrm{D}ij}\cdot {K}_{\mathrm{S}ij}}$$

14 $${\varnothing}_{\mathrm{P}\mathrm{T}}=\frac{1}{{\sum}_{j=2}^{n}{\sum}_{i=1}^{j-1}\frac{{V}_{\mathrm{F} ij}}{{\varnothing}_{\mathrm{P}ij}}}$$

Despite this improvement from the Furnas model, it relies on simplifying assumptions regarding particle arrangement and statistical placement, which limit its accuracy for heterogeneous UHPC systems. As a result, its application remains theoretical and is rarely used in UHPC mixture design.

#### De Larrard model

De Larrard has developed three packing models: the Linear Packing Density Model (LPDM), the Solid Suspension Model (SSM), and the Compressive Packing Model (CPM). Initially, the LPDM was developed and subsequently transformed into the SSM. Later, the LPDM was further refined, resulting in the CPM. Among these, the CPM represents a significant advancement by introducing the concept of compaction energy, allowing the distinction between theoretical (virtual) and achievable (real) packing densities. This is particularly relevant for UHPC, where processing conditions influence final material properties.

##### LPDM

The LPDM [130, 131] improved upon the Furnas model by extending the two-component model to combine the use of multi-components with the geometrical interaction between particles [127]. The packing density for a binary mixture is always the smaller of the two calculated packing densities by the Furnas model, with the size class having the lowest (${\varnothing}_{\mathrm{P}}$) being termed the dominant size class. This occurs because, in Fig. 5 (a), the fine particles cannot fully fill the space between the coarse particles due to an insufficient number of fine particles. Similarly, in Fig. 5 (b), there isn’t enough space between the coarse particles to accommodate the small particles if they are fully packed. The packing density is calculated as shown in Eq. (15), where i=1 always denotes the largest particle [127].

15 $$\varnothing_{\mathrm p}=\mathrm{Minimum}_{i=1}^n\left\{\frac{\varnothing_{i}}{1-\left(1-\varnothing_{i}\right)\Sigma_{j=1}^{i=1}V_{Fj}-\Sigma_{j=i+1}^{n}V_Fj}\right\}$$

This model is valid only when ${d}_{i}\gg {d}_{i+1}$. If this condition is not fulfilled, the packing density of the mixture will also depend on the diameter ratios of the size classes. Geometrical interaction is implemented in the model as the influence of a single size class on the dominant size class i. When small particles disrupt the packing density of the larger dominant size class i, the local expansion of the packing of the larger particles due to the introduction of the smaller ones (loosening effect) is expressed via the function f(i,j). Conversely, when small particles are fully packed, the packing density of the dominant small particles will be lower near a large particle due to the wall effect, which is expressed using the function g(j,i). The packing density can then be calculated using Eq. (16).

16 $$\varnothing_{\mathrm p}=\mathrm{Minimum}_{i=1}^n\left\{\frac{\varnothing_{i}}{1-\left(1-\varnothing_{i}\right)\Sigma_{j=1}^{i=1}g\left(j,1\right)rj-\Sigma_{j=i+1}^{n}f\left(i,j\right)rj}\right\}$$

##### SSM

In the SSM [132], a random packing of particles is considered akin to a suspension of high and finite viscosity. Consequently, the reference-specific packing densities are shifted toward higher values [132]. The relationship between the solid content of a monodisperse suspension (ϕ) and its relative viscosity (${\eta}_{\mathrm{r}}$) is given in Eq. (17).

17 $${\eta}_{\mathrm{r}}=\mathrm{exp}\left(\frac{2.5}{\frac{1}{\phi }-\frac{1}{\beta }}\right)$$

where β is the maximum packing, and ϕ is the random packing. Using β=0.74 and ϕ=0.64, then ${\eta}_{\mathrm{r}}=1.36\times {10}^{5}={\eta}_{\mathrm{r}}^{\mathrm{r}\mathrm{e}\mathrm{f}}$. The packing density for any mixture is given by Eqs. (18) and (19) [132].

18 $${\eta}_{\mathrm{r}}^{\mathrm{r}\mathrm{e}\mathrm{f}}=\mathrm{exp}\left({\int}_{d}^{D}\frac{2.5 y(t)}{\frac{1}{c}-\frac{1}{c(t)}}\mathrm{d}t\right)$$

19 $$c(t)=\frac{\beta (t)}{1-{\int}_{d}^{t}y\left(x\right)f\left(\frac{x}{t}\right)\mathrm{d}x-[1-\beta \left(t\right)]{\int}_{t}^{D}y\left(x\right)g\left(\frac{t}{x}\right)\mathrm{d}x}$$

where c is the packing density; t is the particle size; y(t) is the voluminal size distribution of the particle mixture; *d* and *D* are respectively the minimum and maximum particle sizes; and β(t) is the virtual specific packing density of *t*-size grains as calculated from α(t), which is the specific packing density of particles t, and the experimental one is calculated from Eq. (20) [132].

20 $${\eta}_{\mathrm{r}}^{\mathrm{r}\mathrm{e}\mathrm{f}}=\mathrm{exp}\left(\frac{2.5}{\frac{1}{\alpha (t)}-\frac{1}{\beta (t)}}\right), \mathrm{f}\mathrm{o}\mathrm{r} d\le t\le D$$

When the *t*-size class consists of different particle types, each one characterized for $i=1 \mathrm{t}\mathrm{o} N$ by its own partial volume ${y}_{i}(t)$, the overall packing density β(t) is defined by Eq. (21) [132].

21 $$\frac{1}{\beta (t)}=\sum_{i=1}^{N}\frac{{y}_{i}(t)}{{\beta}_{i}(t)}$$

##### CPM

In the CPM [133], β is the maximum potential packing density of a mixture if the particles are placed one by one. However, since in real applications the particles are not placed one by one, and they depend on the compaction and energy applied, this model introduced a new parameter K which is the compaction energy. The general equation for this model for a mixture containing “n” size classes with category “i” is the dominant one, and including the loosening and wall effects by incorporating ${a}_{ij}$ and ${b}_{ij}$, is shown in Eqs. (22, 23 and 24) [127].

22 $${\beta}_{ti}=\frac{{\beta}_{i}}{1-\sum_{j=1}^{i-1}\left[1-{\beta}_{i}+{b}_{ij}{\beta}_{i}\left(1-\frac{1}{{\beta}_{j}}\right)\right]{r}_{j}-\sum_{j=i+1}^{n}\left[1-\frac{{a}_{ij}{\beta}_{i}}{{\beta}_{j}}\right]{r}_{j}}$$

23 $${a}_{ij}=\sqrt{1-{\left(1-\frac{{d}_{j}}{{d}_{i}}\right)}^{1.02}}$$

24 $${b}_{ij}=1-{\left(1-\frac{{d}_{i}}{{d}_{j}}\right)}^{1.5}$$

For a mono-sized particle, class ${\beta}_{j}$ can be determined from the experimentally determined packing density, as shown in Eq. (25) [127].

25 $${\varnothing}_{j}=\frac{{\beta}_{j}}{\left(1+\frac{1}{K}\right)}$$

The virtual packing density is higher than the real packing density, which depends on the compaction energy. To account for this, the compaction index K is introduced, which is dependent solely on the applied compaction to determine the real packing density. As K tends to infinity, the real packing density (${\varnothing}_{t}$) approaches to the virtual packing density ${\beta}_{t}$. The real packing density (${\varnothing}_{t}$) can be determined from Eq. (26) [127].

26 $$K=\sum_{i=1}^{n}{K}_{i}=\sum_{i=1}^{n}\frac{\frac{{V}_{\mathrm{F}i}}{{\beta}_{i}}}{\frac{1}{{\varnothing}_{t}}-\frac{1}{{\beta}_{ti}}}$$

Despite their theoretical rigor, De Larrard models remain fundamentally discrete and require extensive input parameters, including particle-specific packing densities and interaction coefficients, which limit their practical implementation in UHPC mixture design.

### Continuous models

Continuous packing models describe the particle size distribution using mathematical functions, enabling a smoother and more realistic representation of granular systems. These models are particularly well suited for UHPC, where materials span multiple size scales—from nanoscale SF to microscale powders and mineral fillers.

#### Fuller model

The Fuller model [134] represents one of the earliest continuous approaches, defining an ideal gradation curve based on a power-law relationship between particle size and cumulative distribution. A distribution modulus (*q*) of approximately 0.5 is typically associated with minimum void content. The equation for the Fuller model is presented in Eq. (27) [124].

27 $$\mathrm{C}\mathrm{P}\mathrm{F}\mathrm{T}={\left(\frac{d}{D}\right)}^{q}\times 100\mathrm{\%}$$

where CPFT is the cumulative percentage finer than the particle diameter d; D is the maximum particle diameter; and q is the distribution coefficient, typically 0.5 for minimum voids.

While simple and intuitive, the Fuller model does not account for ultrafine particles and is therefore insufficient for accurately describing UHPC systems.

#### Andreasen model

The Andreasen model [135] is an extension of the Fuller model, introducing an optimization to the adjustment factor q that can be experimentally determined to optimize the packing density for specific materials, and it falls in the range of 0.33–0.50 [124]. The Andreasen model is shown in Eq. (28).

28 $$\mathrm{C}\mathrm{P}\mathrm{F}\mathrm{T}={\left(\frac{d}{D}\right)}^{q}\times 100\mathrm{\%}$$

However, similar to the Fuller model, it does not explicitly incorporate the smallest particle size, limiting its accuracy for systems containing nanoscale materials such as SF.

#### Funk & Dinger model (Modified Andreasen model)

The Funk & Dinger model, also known as the Modified Andreasen model [136], further refines the Andreasen model by incorporating the smallest particle diameter ${D}_{\mathrm{s}}$ into the model. This model provides a more accurate representation of the packing density for systems with a wide range of particle sizes. Equation (29) illustrates the main equation of the model.

29 $$\mathrm{C}\mathrm{P}\mathrm{F}\mathrm{T}=\left(\frac{{d}^{q}-{D}_{\mathrm{s}}^{q}}{{D}^{q}-{D}_{\mathrm{s}}^{q}}\right)\times 100\mathrm{\%}$$

This formulation enables accurate modeling of multi-scale particle systems, making it particularly suitable for UHPC. The distribution modulus (*q*) controls the balance between fine and coarse particles. Lower *q* values increase the proportion of fines, improving flowability and packing density, which is an important consideration for UHPC mixtures with low water-to-binder ratios. Computational analysis determined that the optimal distribution coefficient is approximately 0.37. However, this value decreases the flowability of concrete. To mitigate this effect, a q value of 0.22 is recommended, as this adjustment increases the powder content and reduces the amount of coarse aggregate, thereby enhancing the mix’s flowability [124].

The Modified Andreasen model has been widely adopted in UHPC design due to its simplicity, flexibility, and ability to accommodate a wide range of particle sizes. It provides a practical framework for integrating various materials discussed in Sect. 2, including SCMs and mineral fillers, into a unified particle packing strategy.

### Implications for UHPC design

From a UHPC design perspective, the selection of an appropriate PPDM must reflect the material’s multi-scale and heterogeneous nature. Discrete models provide valuable theoretical insights into particle interactions but are constrained by assumptions of idealized particle geometries and discrete size classes, limiting their applicability to UHPC systems. Moreover, they typically assume that each particle size reaches its theoretical maximum packing density, an ideal rarely achieved in practice, resulting in non-uniform geometric distributions and gaps in particle sizes.

In contrast, continuous models, particularly the Modified Andreasen model, offer a more practical and robust framework for mixture design. By enabling the optimization of continuous PSDs, these models facilitate the development of densely packed systems with reduced porosity, improved mechanical performance, and enhanced durability. This approach eliminates packing discontinuities and provides a more accurate representation of the granular structure.

Although De Larrard’s compressible packing model introduces more advanced considerations, such as particle interaction effects and deformability, it remains fundamentally discrete in nature. Its complexity can deter practical application, leading many designers to favor the simplicity and versatility of continuous models like the Modified Andreasen model for UHPC mixture design.

Importantly, optimized particle packing directly contributes to the development of LC-UHPC. By minimizing voids and improving material efficiency, it becomes possible to reduce cement content without compromising performance. This establishes PPDM as a critical link between raw material selection (Sect. 2) and performance outcomes (Sect. 4), as well as a key enabler for carbon-friendly optimized UHPC formulations.

## UHPC properties

The properties of UHPC are governed by the combined effects of packing density, binder composition, and microstructural refinement. The incorporation of SCMs and mineral fillers (Sect. 2), together with optimized particle packing (Sect. 3), enables the development of a low-porosity matrix that controls both fresh and hardened performance. This section analyzes the influence of these materials on key UHPC properties, with emphasis on governing mechanisms and design implications.

### Flowability

Flowability is a critical parameter in UHPC due to its very low water-to-binder ratio and high powder content. Flowability of UHPC is controlled by particle size distribution, particle morphology, and specific surface area, all of which are directly linked to particle packing.

Materials with spherical morphology and moderate fineness, such as FA, improve flowability through a ball-bearing effect that reduces interparticle friction and viscosity [137]. Similarly, limestone powder enhances flowability by diluting the cement, reducing particle flocculation, and increasing electrostatic repulsion between particles, which improves the overall dispersion [88, 90]. GGBFS and GGP may also enhance UHPC flowability due to their slow early-age reactivity, which reduces the water consumption and leaves more free water in the fresh mixture [43, 56, 58].

In contrast, ultrafine and highly reactive materials, such as SF, significantly reduce flowability due to their high surface area and water demand [28, 138]. Their tendency to agglomerate further limits the effectiveness of superplasticizers, increasing yield stress and viscosity. Materials such as recycled concrete fines also reduce flowability by absorbing mixing water, due to their porous nature and the presence of dry mortar on particle surfaces [94].

Overall, a clear trade-off exists between packing density and workability: increasing fines improves packing and mechanical performance but reduces flowability. This trade-off must be balanced in UHPC design through optimizing the packing density and use of chemical admixtures. Table 3 summarizes the impact of SCMs and mineral fillers on the flowability of UHPC.

**Table 3:** Impact of SCMs and mineral fillers on the flowability of UHPC mixtures

Material	Impact	Refs.	Material	Impact	Refs.
**SF**	Reduces	[26, 28, 138]	**RCWP**	Improves	[84]
**FA**	Improves	[35, 37]	**LP**	Improves	[88, 90]
**GGBFS**	Improves	[43, 139]	**RCF**	Reduces	[94]
**MK**	Reduces	[46, 53]	**CWP**	Reduces	[60, 100, 104]
**GGP**	Improves	[56, 58]	**GRP**	Reduces	[60, 109]
**BC**	Improves	[67]	**RM**	Reduces	[90, 112, 140]
**RHA**	Reduces	[79]	**IOT**	Improves	[116, 118]

### Mechanical properties

#### Compressive strength

The compressive strength of UHPC (often exceeding 150 MPa) is primarily attributed to its high packing density, low porosity, and refined microstructure [141]. The elimination of coarse aggregates, combined with optimized particle size distributions, minimizes weak regions at the interfacial transition zone and enhances stress distribution within the matrix.

SCMs and mineral fillers play a critical role in strength development through both filler effects and pozzolanic reactions, which promote the formation of additional C–S–H gel and densify the interfacial transition zone [5, 142].

SF is the most effective strength-enhancing SCM, with an optimal replacement level at 20%. Beyond this threshold, excessive fines increase water demand and may induce microcracking, reducing strength [25, 26, 28, 138].

FA [37, 143] and GGBFS [42, 144] contribute primarily to long-term strength through slower pozzolanic and latent hydraulic reactions, respectively, often at the expense of early-age strength.

RHA particles fill pores and densify the microstructure, while higher contents may induce dilution effects, with optimal replacements generally around 20% [78].

Inert fillers such as limestone powder improve strength at moderate dosages through nucleation and packing effects but reduce strength at high replacement levels due to dilution [145]. GRP can also enhance compressive strength (by up to 19%) by refining the microstructure and reducing porosity [60].

IOTs improve strength at moderate dosages (up to 10%) but lead to diminishing returns in higher replacement ratios due to cement dilution effects [118].

A consistent trend across studies is the existence of optimal replacement thresholds, beyond which strength declines due to reduced clinker content or increased porosity. These thresholds are strongly influenced by particle packing and the composition of the used SCM.

#### Tensile and flexural strength

The tensile and flexural behavior of UHPC is governed by matrix densification and fibre–matrix interaction. SCMs enhance these properties primarily by refining the interfacial transition zone and improving stress transfer between the fibres and the matrix [146]. This densification improves load transfer and limits microcrack development, eventually contributing to superior tensile performance [147].

SF and MK significantly enhance tensile and flexural strength at moderate replacement levels by reducing porosity and strengthening the interfacial transition zone. However, excessive dosages increase brittleness and autogenous shrinkage, leading to reduced performance [45–47, 53, 138]. Other materials, such as GGBFS [42], GGP [54, 56, 60, 139], and BC [148], provide moderate improvements by contributing to microstructural refinement and internal curing. Notably, BC can enhance tensile performance at low dosages due to its porous structure, which promotes hydration, but becomes detrimental at higher replacement levels [148]. Overall, the tensile performance of UHPC reflects a balance between matrix densification and crack resistance, both of which are influenced by particle packing and binder composition.

#### Elastic modulus

UHPC exhibits a higher elastic modulus compared to conventional concrete (by 20%–70%) due to its dense microstructure and reduced porosity. Unlike conventional concrete, where stiffness is largely governed by coarse aggregates, UHPC relies on a highly compacted matrix [149].

SCMs such as SF [26, 28] and MK [45] enhance stiffness by improving packing density and refining the interfacial transition zone. Similarly, mineral fillers such as GRP and CWP contribute to improved modulus by improving the packing density and their semi-pozzolanic properties, which contribute to the secondary hydration [60].

However, the influence of SCMs on elastic modulus is generally less pronounced than on compressive strength, reflecting the dominant role of overall matrix density. Table 4 summarizes the effects of SCMs and mineral fillers on compressive, tensile, and flexural strengths, as well as elastic modulus, highlighting typical replacement ranges and performance trends.

**Table 4:** Impact of SCMs and mineral fillers on the compressive, tensile, and flexural strengths, in addition to the elastic modulus of UHPC mixtures

Material	Typical replacement	Impact on performance				Refs.
		*Compressive* *strength*	*Tensile* *strength*	*Flexural* *strength*	*Elastic modulus*	
**SF**	5%–25%	Increases 12%–21% at 6%–15%; peaks at 20% (+ 30%); decreases 8% at 25%	Increases up to 20%; decreases beyond 20%	Increases up to 10%; decreases beyond 20%	Increases 3%–11% at 5%–20%	[25, 26, 28, 29, 138]
**FA**	10%–70%	Increases 15%–54% at 10%–40%; decreases beyond 20%–30% in some studies	Decreases at 20%–70%	Decreases at 20%–70%	Decreases at 20%–70%	[35–37, 143]
**GGBFS**	30%–50%	Increases to 150 MPa at 30% (long-term); decreases 13% at 50% (90 days)	Increases 25% at 30%; decreases 1% at 50%	Mirrors tensile strength trend	-	[42, 144]
**MK**	5%–20%	Increases 32% at 15% (56 days); decreases slightly at 20%	-	Increases by 129%–148% at 5–15%; decreases 10% at 20%	Increases 6% at optimal dosage	[45–47, 50, 53]
**GGP**	10%–50%	Increases 8% at 20% (56 days), 13% (91 days); decreases with coarse particles	Increases slightly at 10%–30%	Increases slightly at 10%–30%	Increases slightly when finely ground	[54, 56, 58, 60, 63, 150]
**BC**	0.25%–2%	Increases 5%–8% at 0.25%–2% (up to 19% in some studies), decreases above 2%	Increases up to 25% (optimized mixtures)	Increases 15%–17% at 1%–2%; decreases beyond 2%	Increases 14% (optimized mixtures)	[67, 74, 148]
**RHA**	10%–50%	Increases 20% at 20% cement replacement; 15% at 67% SF replacement (at 28 days)	Increases 10% at 20%	Increases at an early age, stable long-term	Increases 6% at 20%	[78, 79]
**RCWP**	5%–60% cement/10%–30% sand	Increases 7% at 5% cement; decreases sharply at more than 10%	Mirrors compressive strength trend	Highest at 5% cement; similar to control at 10%	-	[84–87]
**LP**	Up to 80%	Increases 4% at 40%; decreases to 47% at 80%	-	-	-	[145]
**RCF**	11%–100%	Highest at 11%; decreases 11%–13% at 40%–60% sand replacement; decreases 8%–14% replacing basalt	Max difference < 0.7 MPa (sand replacement)	Max difference < 1 MPa (sand replacement)	Max difference < 4 GPa (sand replacement)	[91, 92, 94–96]
**CWP**	10%–40%	Decreases 8%–25% at 10%–30% (28 days); increases 6%–9% (90 days)	Increases 14% at 26%	Increases 14% at 26%; up to 31% (sand replacement)	Increases 7% at 26%	[60, 100, 102, 104, 105]
**GRP**	20%–100% sand/10%–30% cement	Increases 24% at 26% cement + 100% sand; 9% at 35%–70% quartz sand; 5% at 100% sand	Increases 32% at 26% cement + 100% sand; 24% at 35% quartz sand	Increases 30% at 26% cement + 100% sand; 6% at 35% sand	Increases 11% at 26% cement + 100% sand	[60, 108, 109]
**RM**	2.5%–60%	Peaks at 20%; declines beyond 20%	Peaks at 15%–20%; decreases at higher dosages	Peaks at 15%–20%	-	[112, 113, 115, 151–154]
**IOT**	10%–40% cement/20%–100% sand	122–141 MPa at 10% cement; similar to control at 20%; decreases 8% at 30%; maximum at 40% sand	-	-	-	[116–118, 121]

### Durability aspects

#### Water permeability

In UHPC, water permeability is a key factor influencing durability and structural integrity. Due to its optimized particle structure and exceptionally low water-to-binder ratio, typically below 0.25 [155], UHPC exhibits a highly dense microstructure, significantly reducing water permeability compared to conventional concrete [156, 157]. This low permeability is essential for preventing the ingress of aggressive agents, such as chlorides and sulfates, which can induce reinforcement corrosion and deteriorate the concrete matrix [157, 158].

Several factors influence the permeability of UHPC, including the type and dosage of SCMs and the overall mixture design. Incorporating SF and other SCMs enhances both mechanical properties and durability by refining the pore structure and promoting secondary hydration, thereby further reducing permeability [159, 160]. As a result, the permeability of UHPC is significantly lower than that of conventional concrete (UHPC can be as low as 0.000 5 cm/s compared to 0.001 5–0.005 0 cm/s for conventional concrete) [156, 157, 160]. This characteristic makes UHPC particularly suitable for structures exposed to aggressive environments, such as marine infrastructure and transportation systems subjected to de-icing salts, where excessive water permeability can accelerate deterioration [158, 161].

#### Chloride penetration

Chloride penetration is a critical durability concern in structures exposed to de-icing salts, seawater, or other chloride-rich environments, as it can initiate corrosion of steel reinforcement. Chloride ions migrate primarily by diffusion, which depends on concrete permeability, moisture content, and the presence of cracks [162]. In conventional concrete, the high porosity allows chloride ions to penetrate more rapidly, increasing the risk of steel corrosion [163]. In contrast, UHPC exhibits a highly dense microstructure due to its low water-to-binder ratio and optimized particle packing, which limits chloride diffusion compared to conventional concrete [164].

Incorporating SCMs such as SF, FA, or GGP further reduces chloride penetration in UHPC by refining the pore network, decreasing pore connectivity, increasing tortuosity, decreasing capillary porosity, and densifying the interfacial transition zone [165–167]. While cracks provide direct pathways that accelerate chloride ingress [168, 169], UHPC’s dense matrix and improved interfacial transition zone substantially delay the onset and progression of chloride-induced corrosion, making it far more resistant than conventional concrete in aggressive environments.

#### Autogenous shrinkage

Autogenous shrinkage arises from the chemical processes associated with hydration and self-desiccation, resulting in a reduction in volume without external drying influences [170]. This is pronounced in UHPC due to its low water-to-binder ratio and high binder reactivity, which is necessary to achieve its high strength and durability [171, 172]. While autogenous shrinkage also occurs in conventional concrete, its effect is relatively minor because higher water content limits self-desiccation. In UHPC, the onset of shrinkage begins immediately after setting, as hydration progressively consumes water within the pore structure, reducing internal relative humidity and generating internal stresses [171, 173]. Most autogenous shrinkage develops within the first few weeks, with significant implications for cracking and long-term performance [174, 175].

Autogenous shrinkage in UHPC is influenced by cement type, the proportion of SCMs and mineral fillers, water-to-binder ratio, and curing temperature. It can be mitigated by reducing the hydration heat, refining the pore structure, and promoting internal curing (e.g., using BC). For instance, the inclusion of mineral fillers like recycled concrete fines has been shown to mitigate autogenous shrinkage by enhancing hydration and refining the microstructure [91, 94].

#### Freeze–thaw resistance

The freeze–thaw process refers to the cyclical freezing and thawing of water within a material, such as concrete or soil, due to temperature fluctuations. This phenomenon is particularly critical in cold climates, where subfreezing temperatures lead to ice formation within the material’s pore structure. Repeated freeze–thaw cycles induce both physical and chemical changes, potentially compromising the material’s integrity [176, 177].

In conventional concrete, freeze–thaw exposure can cause cracking, spalling, surface scaling, and a reduction in compressive strength. When water freezes, it expands by 9%, generating internal pressure in the saturated pores. This expansion creates micro-cracks that may grow with continued cycling, especially in the presence of de-icing salts, which intensify the severity of freeze–thaw action [176–178]. The freeze–thaw resistance is influenced by concrete composition, density, and the use of air-entraining agents. Air-entraining agents create air voids that provide space for ice expansion, thereby reducing internal stresses and mitigating freeze–thaw damage [178, 179].

For UHPC, freeze–thaw behavior differs significantly from that of conventional concrete due to UHPC’s dense matrix, low water-to-binder ratio, and ultra-low permeability [180]. These characteristics greatly limit the amount of freezable water within the pore network, reducing the risk of internal ice formation. Numerous studies have reported minimal mass loss and negligible reductions in mechanical properties after hundreds of freeze–thaw cycles, demonstrating UHPC’s inherent resilience under such conditions [181]. However, when UHPC becomes prematurely saturated or when microcracking exists due to early-age shrinkage, its freeze–thaw resistance may be affected. In such cases, the presence of SCMs can refine the pore structure and enhance performance. For instance, SCMs such as FA contribute to improved freeze–thaw resistance by refining the pore structure and reducing water ingress [176, 182].

#### Sulfate attack

Sulfate attack refers to the chemical deterioration of cementitious materials caused by the ingress of sulfate ions from external sources such as sulfate-rich soils, groundwater, seawater, or industrial wastewater. Sulfate ions react with hydration products, particularly calcium hydroxide and calcium aluminate hydrates, to form expansive products such as gypsum and ettringite. The formation of these products generates internal stresses that can lead to expansion, cracking, spalling, and progressive loss of mechanical strength [183–185].

In conventional concrete, sulfate resistance is strongly influenced by permeability, cement composition, and the availability of reactive aluminate phases. Concrete with high permeability facilitates sulfate ingress, accelerating the formation of expansive reaction products and subsequent deterioration. Mixtures containing high contents of C_3_A are generally more susceptible to sulfate attack, whereas the incorporation of SCMs improves sulfate resistance by reducing permeability, consuming calcium hydroxide through pozzolanic reactions, and refining the pore structure [186, 187].

UHPC exhibits substantially higher resistance to sulfate attack compared to conventional concrete owing to UHPC’s extremely dense microstructure, ultra-low permeability, and reduced connectivity of capillary pores, which significantly restrict sulfate penetration [188]. Furthermore, the extensive use of SCMs lowers the calcium hydroxide content (which triggers sulfate attack reactions) while producing additional C–S–H gel [187], thereby reducing the availability of phases susceptible to sulfate reactions. Numerous UHPC studies have reported negligible expansion and minimal degradation of mechanical properties after prolonged sulfate exposure, demonstrating the excellent sulfate durability of UHPC [189, 190]. The selection of SCMs also plays an important role in further enhancing sulfate resistance. SCMs such as SF, GGBFS, GGP, and MK have been shown to improve sulfate resistance by refining the pore structure, lowering permeability, and reducing the content of reactive calcium aluminate phases available for expansive reactions.

#### ASR

ASR is a chemical reaction between alkali hydroxides present in the cement pore solution and reactive forms of silica contained in certain aggregates. This reaction produces an expansive alkali-silica gel that absorbs water and swells, generating internal stresses that lead to expansion, cracking, and progressive deterioration of the matrix [191, 192]. The severity depends on the availability of reactive silica, alkali content, and sufficient moisture to sustain gel expansion.

In conventional concrete, ASR is one of the most significant durability concerns for structures exposed to humid environments. Once initiated, the expansive gel develops within the aggregate and surrounding cement paste, causing microcracking that increases permeability and accelerates the ingress of further harmful agents. The susceptibility of concrete to ASR is influenced by aggregate mineralogy, cement alkali content, and pore solution chemistry. Incorporating SCMs has been recognized as one of the most effective mitigation techniques as they consume calcium hydroxide through pozzolanic reactions, reduce pore solution alkalinity, refine the pore structure, and limit moisture transport [187, 193].

UHPC exhibits substantially improved resistance to ASR than conventional concrete due to its dense microstructure, ultra-low permeability, and optimized particle packing, which restrict moisture ingress and the transport of alkalis within the matrix [188]. Furthermore, UHPC typically incorporates large amounts of SCMs (i.e., SF, GGBFS, and GGP), which reduce pore solution alkalinity and suppress the expansion associated with ASR. This is particularly relevant for GGP, which, despite being itself a source of reactive silica, has been shown to behave as an ASR-mitigating SCM rather than a reactivity risk when finely ground (typically < 300 μm); its high fineness promotes rapid pozzolanic consumption of calcium hydroxide, converting it into stable secondary C–S–H before sufficient alkali-silica gel can accumulate to cause damaging expansion [66]. Numerous studies have reported negligible expansion and limited microstructural damage in UHPC subjected to accelerated ASR testing, demonstrating its excellent resistance to alkali-induced deterioration [155, 194]. Nevertheless, when highly reactive aggregates are used in conjunction with high-alkali cement, ASR cannot be completely disregarded. Therefore, proper aggregate selection and the incorporation of adequate SCM content remain essential to ensure long-term durability.

Table 5 summarizes the influence of SCMs and mineral fillers on key durability indicators.

**Table 5:** Impact of SCMs and mineral fillers on the durability and properties of UHPC mixtures

Material	Impact on hardening, durability, and carbon footprint properties	Refs.
**SF**	Increases autogenous shrinkage and reduces drying shrinkage. Reduces total and basic creep. Enhances durability by decreasing chloride ion penetration and water permeability	[26, 28]
**FA**	Reduces water permeability as replacement increases (notably at 40%). Reduces CO_2_ footprint by 33% and 58% at 40% and 70% replacement, respectively. Maintains low carbonation depth (< 0.5 mm) up to 70% replacement	[35, 36]
**GGBFS**	Enhances packing density and reduces bleeding and segregation. Improves setting time and reduces shrinkage risk with high fineness	[43]
**MK**	Reduces chloride ion permeability by 23%. May increase autogenous shrinkage if not fibre-reinforced	[45–47, 50, 53]
**GGP**	Fine particle size improves resistance to chloride penetration and water permeability. Nano-sized particles increase autogenous shrinkage. Micro-particles initially increase, then reduce shrinkage	[54–56, 59, 60]
**BC**	Decreases early- and late-age shrinkage. Reduces water and capillary absorption by 50%–57% at 1% replacement	[67, 74]
**RHA**	Reduces autogenous shrinkage; at 20% replacement, eliminates shrinkage after 15 days. Reduces water permeability (6%) and chloride penetration (13%)	[78, 195]
**RCWP**	Reduces autogenous shrinkage by 43%–57% at 5%–60% cement replacement. Reduces chloride penetration by up to 32% when used as sand replacement	[84–87]
**LP**	Reduces both initial and final setting times. Enhances early hydration and densifies pore structure up to 60% volume. Excess (> 60%) leads to poor durability	[90, 145]
**RCF**	Reduces autogenous shrinkage by up to 59% at 30% quartz sand replacement. Can reduce CO_2_ emissions by 30% at 33% replacement	[91, 92, 94]
**CWP**	Reduces water permeability by 3%–8% and chloride penetration by 9%–10%. Improves long-term microstructure compactness	[60, 105]
**GRP**	Reduces water permeability by up to 19% and chloride penetration by up to 18% at 26% cement + 100% sand replacement	[60, 109]
**RM**	Enhances packing density. Improves chloride ion resistance and water impermeability at optimal dosages (around 15%–20%). Excessive content may harm durability	[112, 196]
**IOT**	Reduces autogenous shrinkage by delaying internal humidity loss. Improves pore structure when used ≤ 60%. At 30% replacement, reduces CO_2_ emissions by 32%	[116]

## Data analysis

To provide insights on the interplay between UHPC’s key compositional indicators, performance metrics, and embodied carbon emissions, a comprehensive database was developed from 200 data entries extracted from peer-reviewed literature [26, 60, 67, 78, 84, 90, 106, 109, 112, 116, 117, 139, 145, 197–201]. The full database, including mixture compositions, performance results, and calculated CO_2_ emissions for each entry, is provided as a supplementary document. Only studies reporting complete mixture compositions, standardized water curing at room temperature, and compressive strength based on cube specimens were included to ensure consistency and comparability; studies using steam or hot-water curing were excluded, as this would introduce a variable independent of mixture design.

These criteria were selected to ensure that the compiled dataset was suitable for the comparative analysis presented in this study. Complete mixture compositions were required because the mixture design ratios analyzed in Sect. 5.1.1 depend on full compositional data. Compressive strength based on cube specimens was required to avoid mixing up mixture-design effects with specimen geometry effects, since cube and cylinder strengths are not directly comparable without a conversion factor. Testing age was not used as an exclusion criterion; instead, compressive strength was captured at multiple ages (7, 28, and 91 days) as separate response variables, allowing age-dependent behavior to be examined directly rather than excluded. Fibre type and content could not be included due to inconsistent reporting across the compiled literature, which is acknowledged as a limitation and is reflected in the reduced predictive accuracy of the model for tensile-related properties, as discussed in Sect. 5.1.3.

The database was compiled through a combination of literature already known to the authors and a targeted search of Scopus and Google Scholar using keywords related to UHPC, supplementary cementitious materials, and low-carbon/sustainable UHPC. Where a source study did not report a specific performance property, only that property was excluded for the corresponding entry, while the remaining reported data were retained; this explains the variation in the number of data points available for different properties. The database was also checked for duplicate or overlapping mixtures across sources, and none were identified.

The dataset was further categorized into industrial byproducts, bio-sourced binders, and hybrid systems, enabling a comparative assessment of how binder type governs both mechanical efficiency and carbon footprint. Rather than serving solely as a descriptive dataset, this compilation provides a basis for identifying governing relationships in UHPC design, particularly the interplay between mixture proportions, performance metrics, and embodied carbon.

### Mechanical, tensile, and durability aspects

While Sect. 3 establishes the theoretical basis for particle packing optimization in UHPC, direct calculation of theoretical packing density (e.g., via the Modified Andreasen or compressible packing models) requires detailed particle size distribution data and material properties that were not reported across the compiled literature sources. The data-driven analysis in this section therefore uses mixture design ratios as practical, empirically available alternatives for particle packing behavior, allowing the packing principles discussed in Sect. 3 to be examined indirectly through their influence on measured performance.

The dataset combines mixture design variables “such as water-to-binder (w/b), cement-to-binder (c/b), water-to-cement (w/c), sand-to-binder (s/b), and sand-to-cement (s/c)” and performance responses, including compressive strength at multiple ages, tensile and flexural strength, elastic modulus, chloride penetration, and water permeability. Among durability indicators, chloride penetration and water permeability were prioritized due to their consistent reporting and their direct relevance to long-term degradation mechanisms. A summary of variables and data availability is presented in Table 6.

**Table 6:** Summary of variables and responses with corresponding number of data points

Variables		Responses	
**Parameter (abbreviation)**	**No. of data points**	**Parameter (abbreviation)**	**No. of data points**
w/b	201	Compressive strength at 7 days (CS7)	196
w/c		Compressive strength at 28 days (CS28)	201
s/c		Compressive strength at 91 days (CS91)	141
s/b		Splitting tensile strength at 28 days (STS28)	153
c/b		Flexural strength at 28 days (FS28)	162
		Elastic modulus at 28 days (EM28)	136
		Chloride penetration at 28 days (CP28)	120
		Water permeability at 28 days (WP28)	119

#### Relationships between mixture variables

**Fig. 7:**
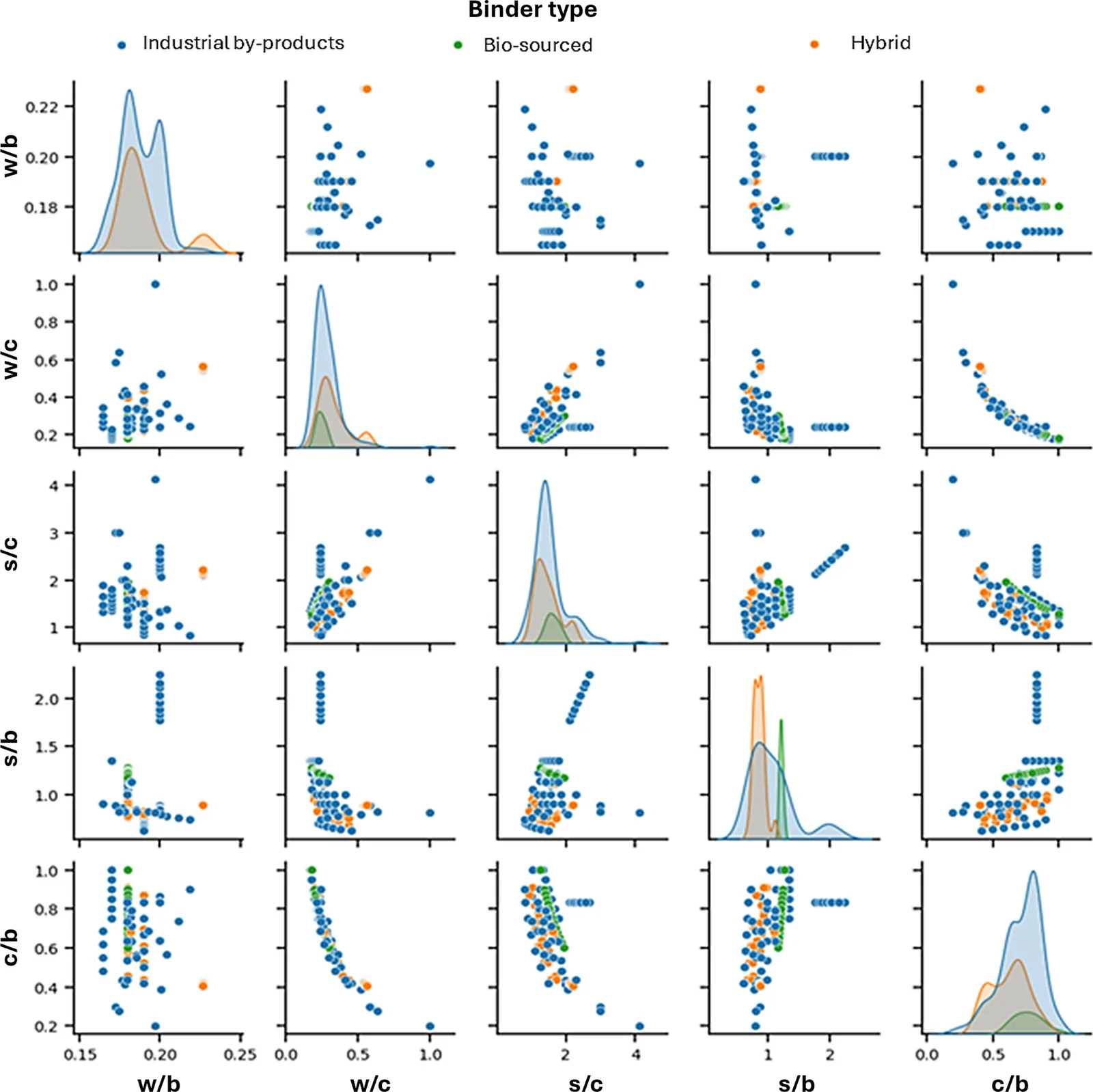
Pair plots of the variables

**Fig. 8:**
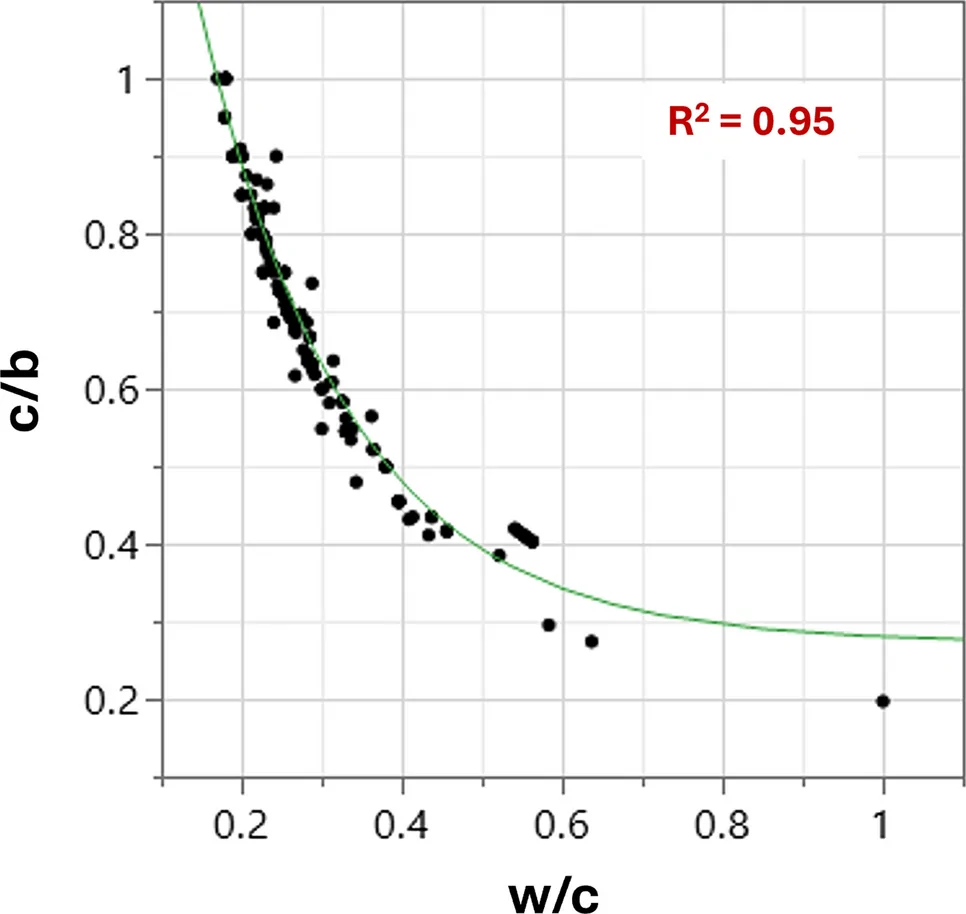
Relationship between c/b and w/c ratios

Exploratory analysis (Fig. 7) reveals that UHPC mixture design is not governed by independent parameters, but by strongly coupled relationships, the most prominent being the inverse dependency between c/b and w/c ratios. This relationship is quantified through an exponential decay model (Fig. 8 and Eq. (30), *R*^2^ = 0.95), indicating that water demand in UHPC is fundamentally controlled by cement fraction rather than total binder composition. Although w/b, w/c, and c/b are mathematically related (w/b = w/c • c/b), this relationship does not constrain the functional form linking c/b and w/c; for a given c/b, w/c could vary independently across mixtures. Therefore, the observed exponential trend reflects an empirical pattern in UHPC mixture design practice rather than a mathematical artifact of the ratio definitions.

This finding has important implications. While SCMs are often introduced to reduce cement content and improve sustainability, the results suggest that their influence on water demand is secondary to that of cement itself. In other words, mixture workability and hydration kinetics remain primarily governed by cement content, regardless of binder type. The derived relationship (Eq. (30)) therefore provides more than a predictive tool; it reflects a fundamental constraint in UHPC mixture design, where reductions in cement content must be carefully balanced against water demand to maintain performance.

A secondary trend between s/c and c/b ratios suggests that increasing cement content is accompanied by reduced sand proportions; however, this relationship is less consistent and appears sensitive to binder composition. This variability highlights the role of SCMs in modifying granular packing, which reinforces the need for binder-based optimization rather than general mix design.

30 $$\mathbf{c}/\mathbf{b}=0.273+1.830{\mathrm{e}}^{-5.437 \mathbf{w}/\mathbf{c}}$$

#### Interdependence of mechanical and durability responses

**Fig. 9:**
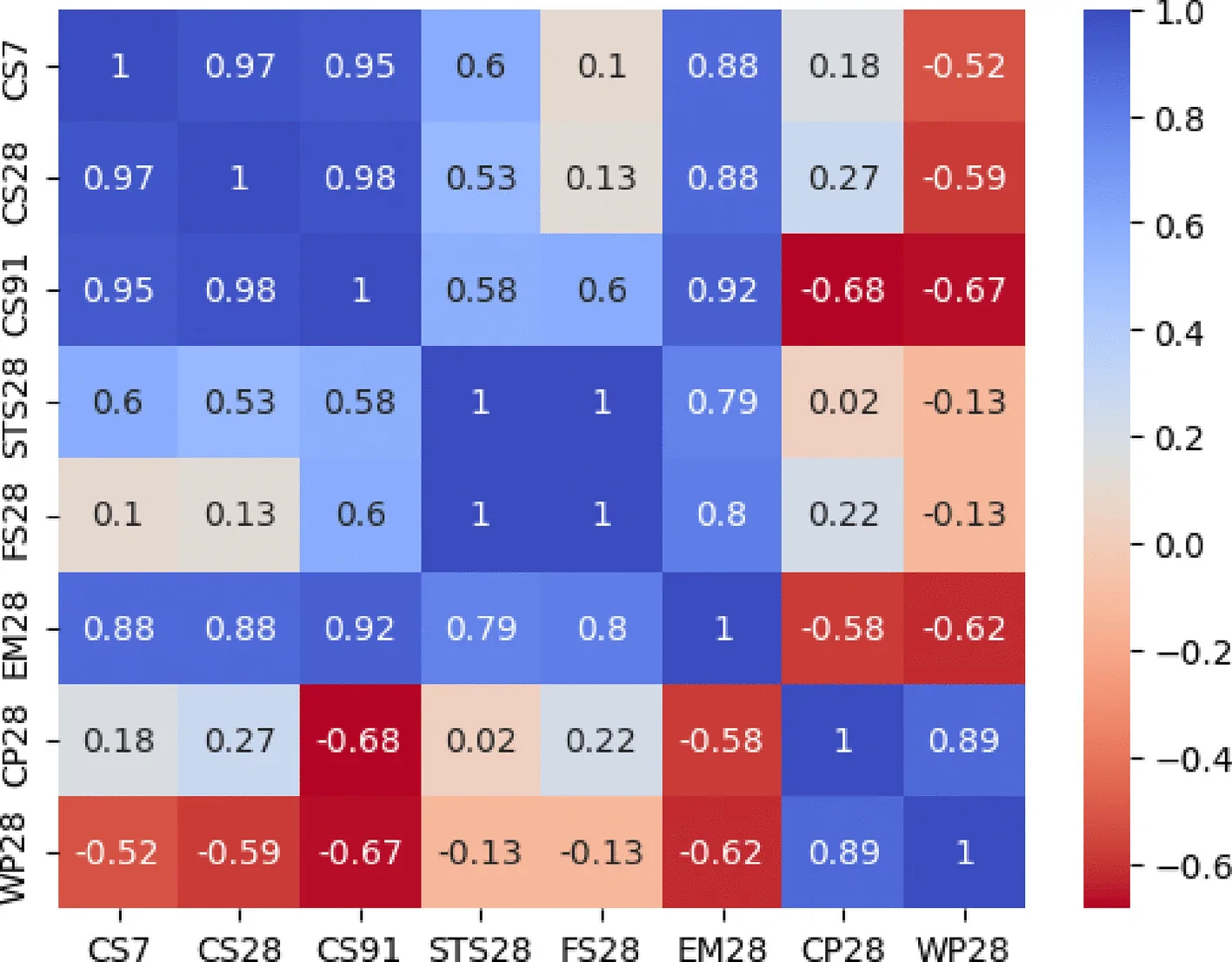
Heat correlation map for the variables

The correlation matrix (Fig. 9) demonstrates that UHPC performance is governed by interdependent mechanical and durability properties. The near-linear relationships between compressive strengths at different ages confirm that early-age strength is a reliable predictor of long-term strength, which reflects consistent hydration and microstructural development.

More importantly, the strong correlations between compressive strength and elastic modulus (up to 0.92) indicate that stiffness in UHPC is largely a function of matrix densification, rather than aggregate contribution, which is typically dominant in conventional concrete. This supports the hypothesis that UHPC behaves as a particle-optimized composite, where microstructural refinement influences both strength and stiffness.

A particularly notable outcome is the perfect correlation between flexural strength and splitting tensile strength, which confirms that both parameters capture the same underlying tensile behavior. This implies that, for modeling and design purposes, one parameter may be sufficient to represent tensile performance, provided fibre-related effects are consistent.

From a durability perspective, the observed negative correlations between permeability-related properties and mechanical performance confirm that strength and durability in UHPC are linked through microstructural density. The strong positive correlation (0.89) between chloride penetration and water permeability further suggests that these parameters are not independent but rather reflect a shared transport mechanism controlled by pore connectivity. This interdependence has practical implications, as durability may be indirectly controlled through strength optimization, provided that microstructural refinement is achieved through appropriate mixture design.

#### Predictive modeling and its limitations

**Fig. 10:**
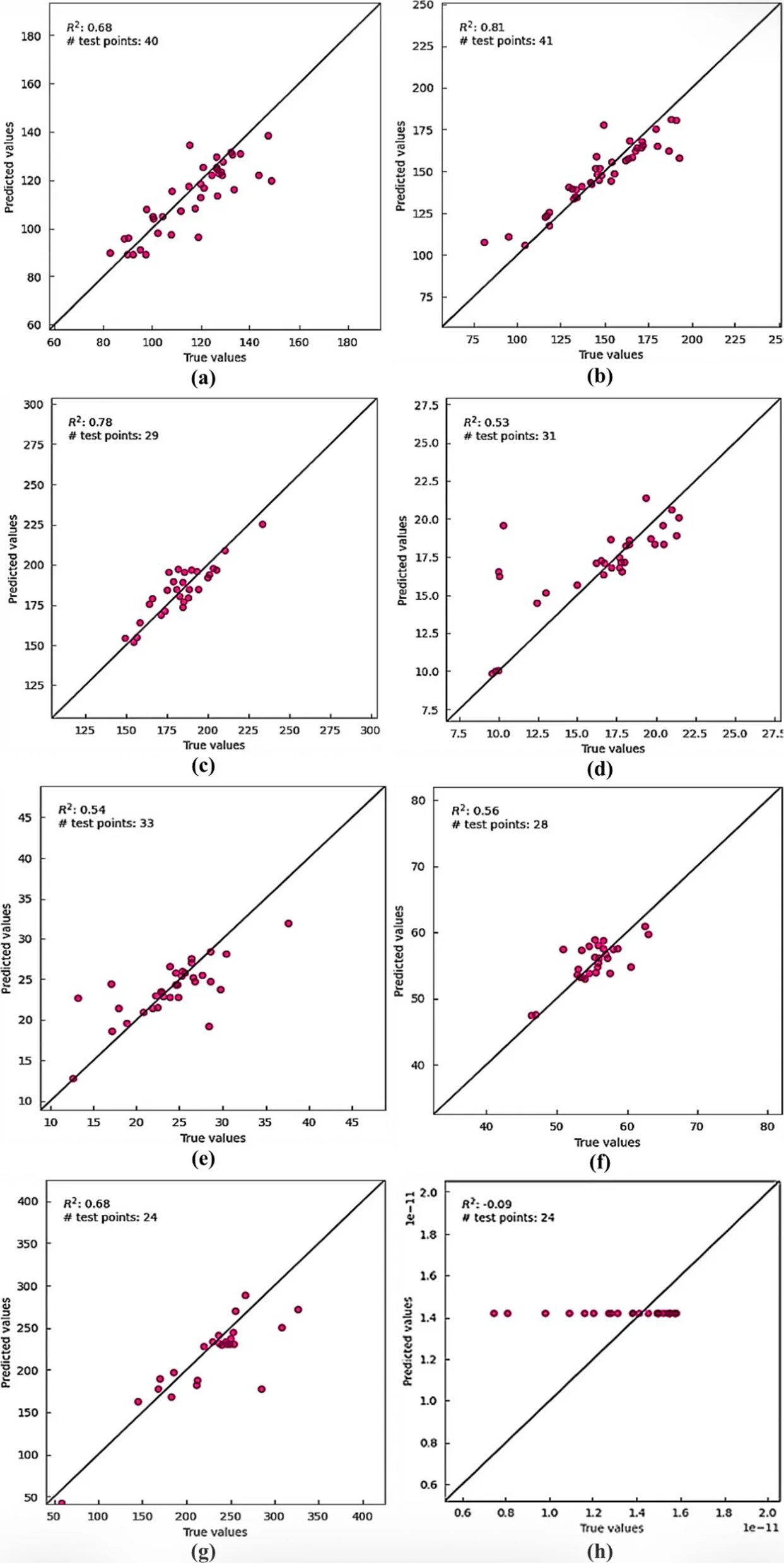
Correlation of variables and responses using Random Forest analysis. **a** 7-day compressive strength. **b** 28-day compressive strength. **c** 91-day compressive strength. **d** tensile strength. **e** flexural strength. **f **elastic modulus. **g** chloride penetration. **h** water permeability

The application of machine learning highlights the non-linear and multivariate nature of UHPC behavior. Several machine learning techniques were tested and compared using the coefficient of determination (*R*^2^) as the primary evaluation metric. Among the evaluated models, the Random Forest (RF) algorithm demonstrated the best performance, indicating that interactions between mixture variables are too complex to be captured by linear or single-parameter models. Figure 10 illustrates the results obtained from the RF analysis.

RF hyperparameters (number of estimators, maximum tree depth, minimum samples per split and leaf, maximum features considered per split, and bootstrap sampling) were optimized using grid search with fivefold cross-validation, applied independently for each predicted property. Model performance was then evaluated on a 20% test set not used during training or hyperparameter selection. Overfitting was mitigated both by selecting hyperparameters based on cross-validated performance and by RF’s ensemble structure, which averages predictions across many decorrelated trees trained on bootstrap samples, reducing sensitivity to noise in any single training subset.

The high predictive accuracy achieved for compressive strength (particularly at 28 and 91 days) confirms that mixture proportions are the governing parameters of the compressive strength. However, the reduced accuracy for tensile-related properties and elastic modulus underscores a key limitation, as these properties are strongly influenced by factors not included in the dataset, such as fibre content, orientation, and interfacial bonding.

Similarly, theoretical packing density metrics discussed in Sect. 3 (e.g., the Modified Andreasen, De Larrard) were not incorporated as input features, as particle size distribution data required to calculate them were not consistently reported across the compiled literature sources. Future work incorporating a database with standardized particle size reporting could enable packing density to be included as a predictive variable, which will strengthen the link between particle packing theory and data-driven performance prediction.

Water permeability represents the most challenging parameter to predict, reflecting its dependence on microstructural features such as pore size distribution and connectivity, which are affected by the degree of hydration of the used SCMs, which cannot be directly inferred from mixture proportions alone. However, its strong correlation with chloride penetration (Fig. 9) provides an important insight, as permeability may be indirectly estimated through more predictable durability indicators, suggesting a pathway for hybrid predictive approaches.

Despite these promising results, it is important to acknowledge that the model’s applicability is constrained by the dataset. Variability in experimental methods, incomplete reporting of key parameters, and uneven representation of binder types limit the generalizability of the findings. As such, the predictive models should be interpreted as data-informed tools rather than universally applicable design equations.

It should be noted that the predictive model does not account for all factors influencing UHPC performance. Parameters such as fibre characteristics, particle packing density, SCM reactivity, and curing regime were excluded due to inconsistent reporting across the compiled literature. Consequently, the model captures the dominant, consistently reported mixture design variables rather than all factors governing UHPC performance.

### Carbon footprint assessment and performance trade-offs

The equivalent CO_2_ emissions (in kg CO_2_) for each material were obtained from published literature reporting cradle-to-gate emissions, which is consistent with common environmental product declaration and life-cycle inventory reporting practice for cement and SCMs (i.e., raw material extraction through to production, excluding downstream stages such as transportation, construction, service life, and end-of-life). For materials lacking direct reported values (such as rice straw ash), emissions were estimated following the same cradle-to-gate logic, based on production processes and energy inputs (see Table 7). Because the dataset was compiled from diverse literature sources, minor inconsistencies in reported boundary conditions cannot be entirely ruled out; this is an inherent limitation of CO_2_ datasets assembled from the literature.

**Table 7:** Equivalent CO_2_ emissions of constituent materials used in UHPC mixtures

Material	CO_2_ emissions (kg CO_2_/kg)	Ref.	Material	CO_2_ emissions (kg CO_2_/kg)	Ref.
Cement	0.930 0	[145]	Nano titanium dioxide	14.750 0	[202]
Silica fume	0.014 0	[139]	Rice straw ash	0.000 2^a^	[203]
Fly ash	0.009 0	[204]	Nano egg-shell powder	0.020 8^a^	[205]
Ferrosilicon	3.440 0	[206]	Sugarcane leaf ash	0.034 0^a^	[109]
Metakaolin	0.400 0	[204]	Olive waste ash	0.058 0^a^	[207]
Limestone powder	0.017 0	[145]	Palm leaf ash	0.034 0^a^	[109]^b^
Biochar	− 1.300 0	[67]	Nano cotton stalk ash	0.034 0^a^	[208]
Rice husk ash	0.103 2	[209]	Quartz sand	0.010 0	[210]
Ground granulated blast-furnace slag	0.019 0	[204]	High-range water reducer	0.720 0	[211]
Granite powder	0.002 3	[212]	Water	0.000 3	[211]
Ceramic waste powder	0.000 0	[213]	Steel fibres	1.496 5	[211]
Ground glass pozzolans	0.083 8	[139]	Quartz powder	0.022 7	[214]
Recycled concrete fines	0.085 8	[215]	Hematite	0.026 68	[216]
Red mud	0.015 0	[113]	Basalt	0.035 7	[217]
Iron ore tailings	0.091 3	[116]	Barite	0.104 8	[216]
Recycled coral waste powder	0.011 1	[86]	Serpentine	0.180 0	[216]

^a ^Values were estimated from the specific energy consumption of the reported production process (e.g., calcination or burning temperature and duration, as described in the cited source), converted to CO_2_-equivalent emissions using process-specific emission factors from the Ecoinvent life-cycle inventory database, accounting for the relevant energy inputs associated with each process

^b ^For palm leaf ash, the same emission estimation process as sugarcane leaf ash was assumed due to similarities in feedstock characteristics and processing methods

It is revealed that cement is the main contributor to the huge carbon footprint of UHPC, followed by steel fibres. Although steel fibres exhibit higher CO_2_ emissions per kg than cement, the exceptionally high cement contents typically used in UHPC mixtures make cement the primary source of embodied carbon. In contrast, SCMs contribute minimally to total emissions, which reflects their importance in enhancing the performance of UHPC and reducing its carbon footprint.

**Fig. 11:**
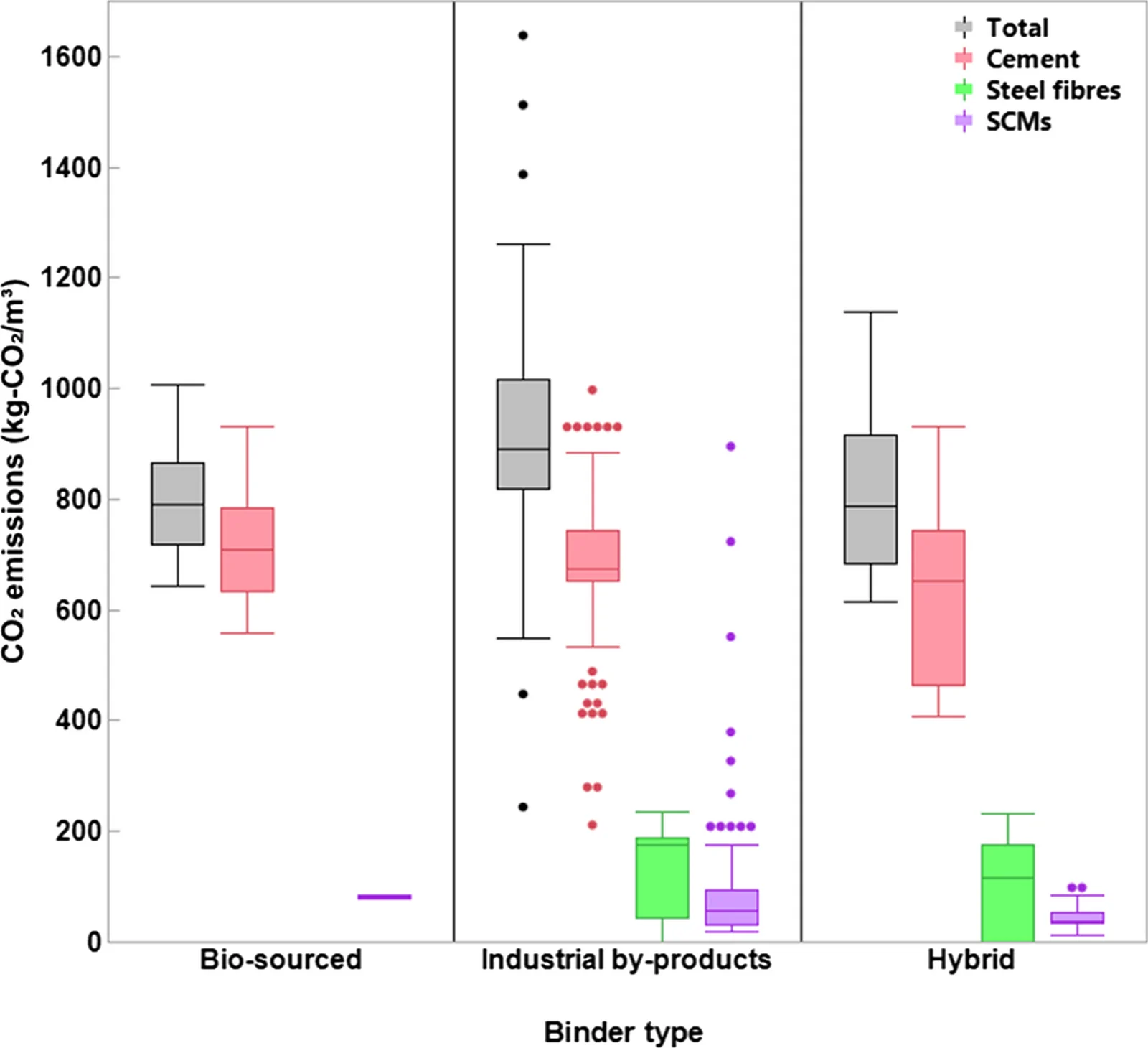
Box plots of CO_2_ emissions by component for UHPC mixtures grouped by binder type (total emissions and contributions of cement, steel fibres, and SCMs shown with outliers)

The box plot analysis (Fig. 11) shows that bio-sourced binders achieve the lowest emissions, but at the expense of reduced strength variability and performance range. Industrial byproducts and hybrid systems exhibit broader distributions, reflecting their ability to achieve higher strengths but with greater variability in emissions.

A key insight emerges from the identification of outliers associated with ferrosilicon, whose high emission factor (3.44 kg CO_2_/kg) disrupts the typical inverse relationship between cement and SCM content. This highlights that not all SCMs are environmentally beneficial, and their selection must consider both performance and embodied carbon.

#### Strength–emission trade-off and cement efficiency

**Fig. 12:**
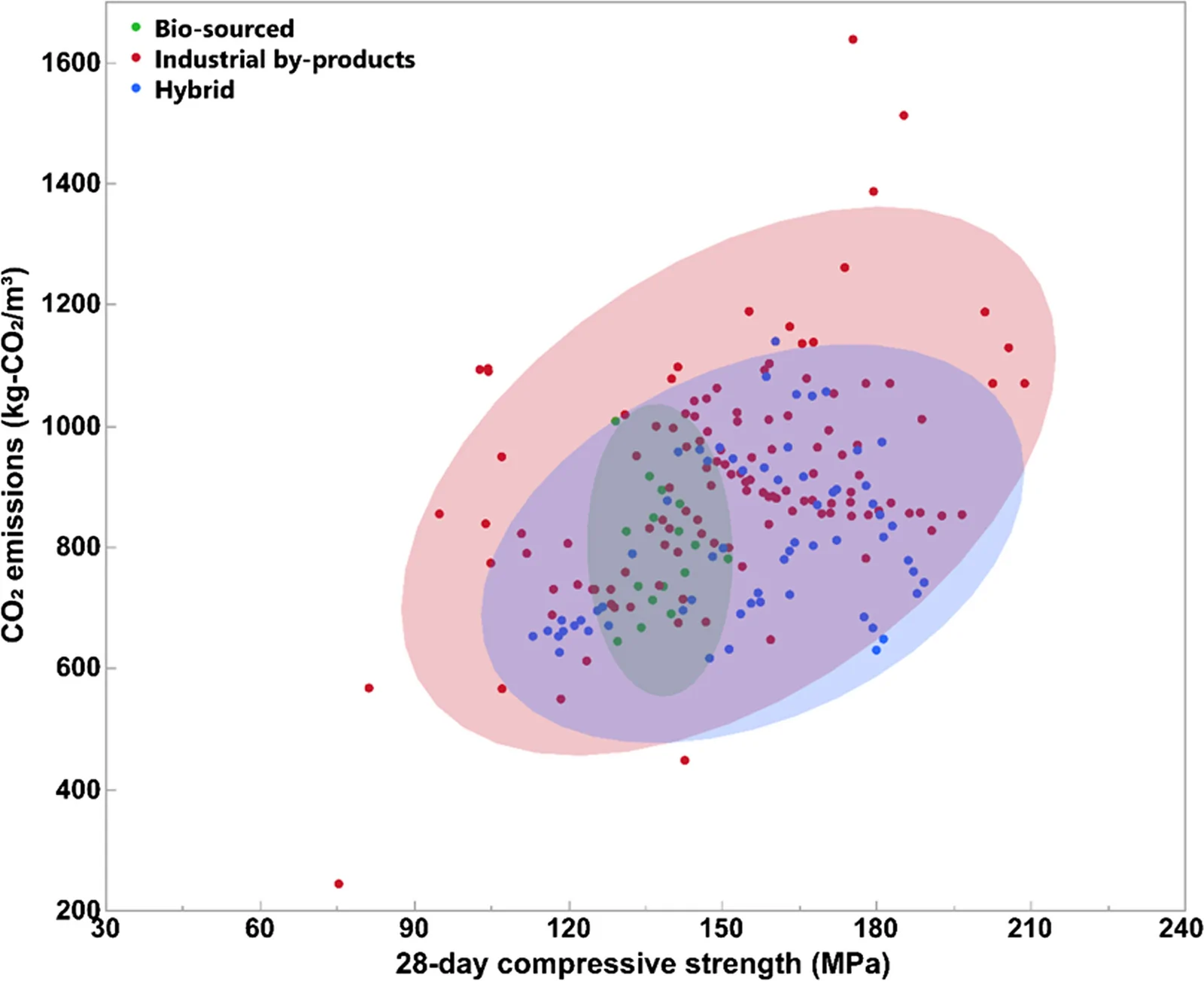
CO_2_ emissions vs. 28-day compressive strength for UHPC mixtures grouped by binder type (with 95% confidence ellipses)

The relationship between CO_2_ emissions and 28-day compressive strength (Fig. 12) illustrates a trade-off in UHPC design: generally, higher strength is associated with higher emissions, but this relationship is not linear. Hybrid systems demonstrate the most balanced performance, achieving high strength with moderate emissions, which suggests that synergistic combinations of SCMs can optimize both properties simultaneously.

**Fig. 13:**
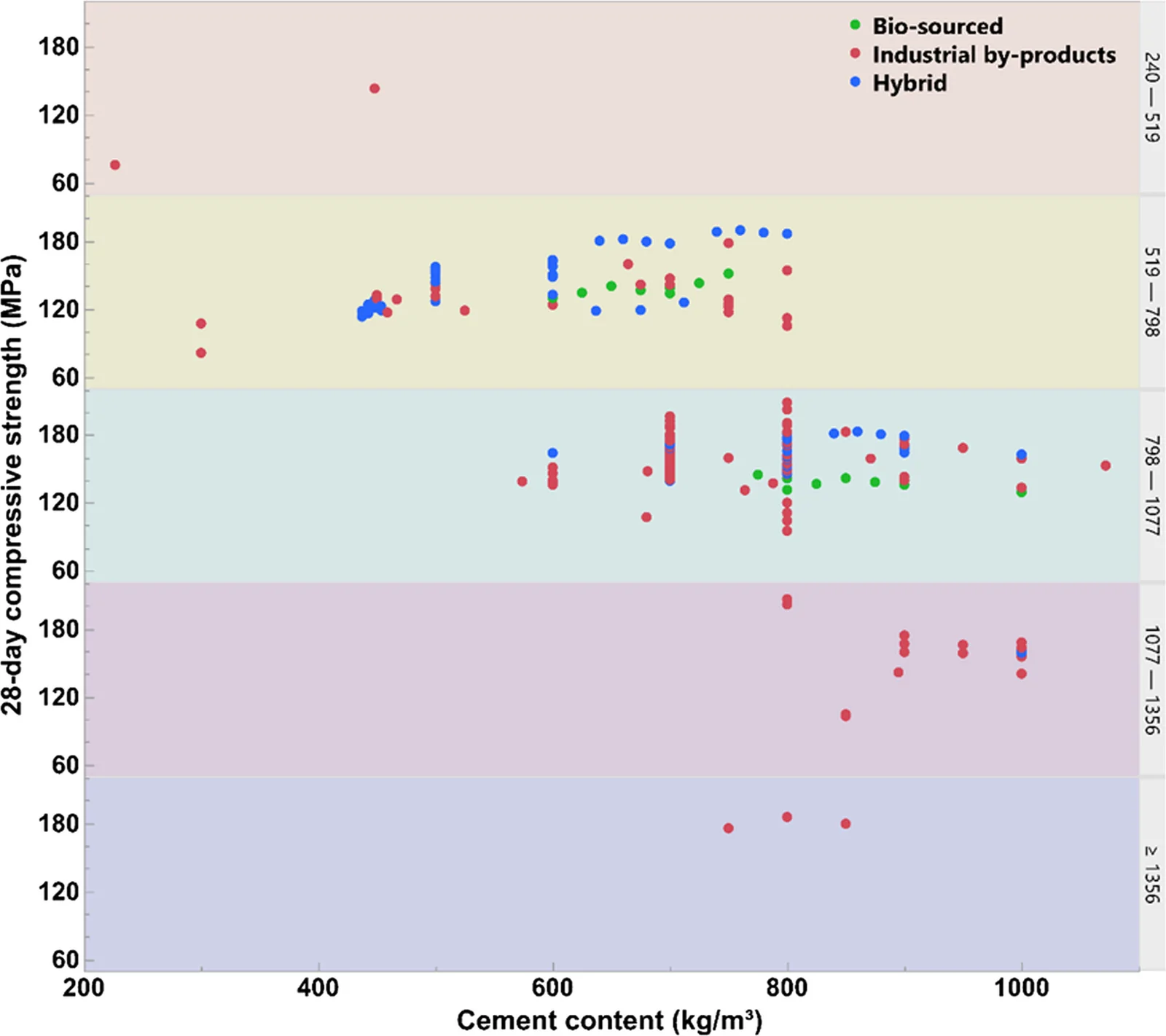
Relationship between cement content and 28-day compressive strength, categorized by CO_2_ emissions in the secondary *y*-axis (in kg CO_2_/m^3^) and color coded by the binder type

Further analysis (Fig. 13) reveals a critical threshold in cement content. While increasing cement dosage enhances strength up to a specific point, beyond approximately 800 kg/m^3^, additional cement content yields diminishing effects, significantly increasing emissions without proportional strength gains. This saturation threshold reflects both limited hydration (only 30%–40% of the cement participates in the hydration process [123]) and a saturation effect consistent with the packing models discussed in Sect. 3, where beyond a given proportion, additional fine material no longer contributes to filling residual voids and instead simply adds volume without densifying the matrix.

Several high-performing mixtures within a cement range of 500–700 kg/m^3^ achieve compressive strengths approaching 150 MPa while maintaining relatively low CO_2_ emissions (600–800 kg CO_2_/m^3^). This outcome is especially clear in hybrid binder systems that combine SF with bio-sourced SCMs such as rice husk ash, sugarcane leaf ash, and olive waste ash. These mixtures demonstrate how synergistic SCMs combinations can offset reductions in cement content without compromising strength, while also substantially lowering the embodied carbon emissions. A similar, though slightly less pronounced effect is observed in mixtures combining SF with industrial byproducts like granite powder and ground glass pozzolan.

These findings have significant implications for UHPC design, as it defines a practical upper limit for cement efficiency, highlight the importance of particle packing over cement quantity, and reinforce the role of SCMs as essential components rather than optional additives.

#### Carbon efficiency and binder optimization

**Fig. 14:**
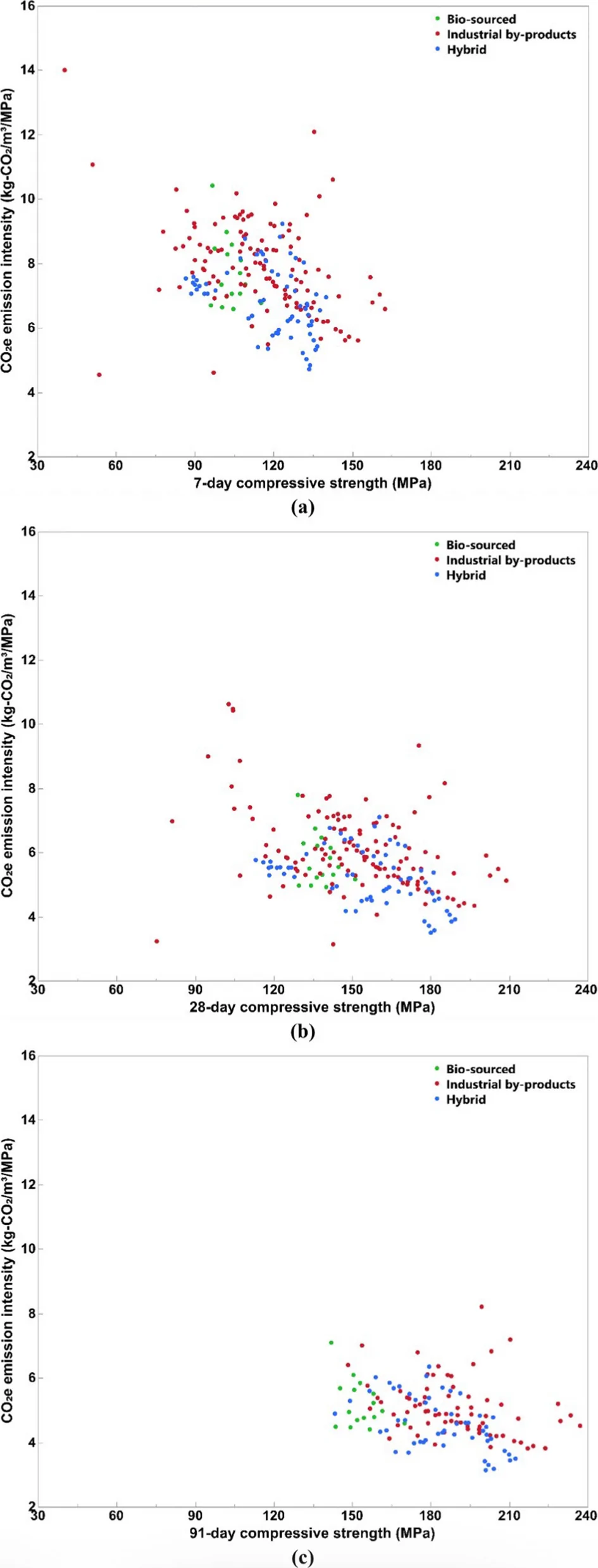
CO_2_ emissions intensity (kg CO_2_/(m^3^•MPa)) of UHPC mixtures across binder types at different ages. **a** 7 days. **b** 28 days. **c** 91 days

The CO_2_ intensity analysis (Fig. 14) provides a more nuanced perspective by normalizing emissions relative to strength (kg CO_2_/(m^3^•MPa)). At early ages, no clear relationship is observed, reflecting variability in hydration kinetics, as most of the SCMs provide late-age development in the compressive strength. However, at 91 days, hybrid binders consistently demonstrate superior carbon efficiency, achieving high strength with lower emissions intensity.

In contrast, while bio-sourced binders are more favorable in terms of their embodied carbon emissions, they achieve lower long-term strength than hybrid systems, indicating that their contribution to long-term hydration and strength development remains limited in current UHPC formulations. Industrial byproducts provide a stronger balance between long-term strength and carbon footprint; however, hybrid systems remain the best overall combination of high strength and reduced carbon footprint.

While several of the relationships identified through this data-driven analysis are consistent with established, qualitative understanding of UHPC behavior—such as the dominance of cement in embodied CO_2_ emissions or the existence of an optimal SCM replacement range—the contribution of this analysis lies in quantifying these relationships across a compiled cross-study database. This includes deriving an explicit predictive relationship between c/b and w/c ratios (Eq. (30)), identifying a specific cement content threshold (~ 800 kg/m^3^) beyond which strength gains plateau while emissions continue to rise, and demonstrating that predictive accuracy itself varies across properties, being strong for compressive strength but limited for tensile behavior and permeability. These quantitative dataset-scale findings were not previously available for UHPC and provide actionable benchmarks for low-carbon mixture design.

## Conclusions

Aimed at unveiling the interplay between UHPC compositional domain space, performance indicators, and embodied CO_2_, this study establishes quantitative relationships between mixture design, mechanical and durability performance, and embodied CO_2_ emissions by integrating a comprehensive literature review with a data-driven analysis of approximately 200 UHPC mixtures. Unlike previous review studies that discuss raw materials and UHPC performance separately, this study combines statistical analysis and machine learning models (i.e., RF) to provide practical insights into low-carbon UHPC mixture optimization. The findings provide quantitative insights on the role of SCMs, particle packing, mixture design parameters, and binder composition on the performance of UHPC and its carbon footprint. Based on the outcomes of this study, the following conclusions can be drawn:

- UHPC performance is governed by synergistic SCM–filler interactions and optimal replacement levels rather than total binder content. Highly reactive SCMs (e.g., SF) give the largest early gains (~ 30% compressive strength at ~ 20% replacement), while slower-reacting SCMs (FA, GGBFS) improve long-term strength (up to 25%) through secondary hydration. All SCMs show non-linear behavior, with performance dropping beyond an optimal range.
- From a long-term perspective, SCM selection must consider supply stability, with GGP emerging as a robust alternative due to its scalability and consistent performance.
- Mechanical and durability properties are strongly interdependent, as expected. Compressive strength correlates strongly with elastic modulus (88%–92%) and inversely with durability indicators such as water permeability and chloride penetration (up to ~ 68%), confirming that UHPC performance is governed by a single densification mechanism rather than independent properties and governed by matrix densification.
- Mixture design parameters (w/c, w/b, c/b, s/c, s/b ratios) possess interdependencies predictive capability. c/b and w/c ratios are strongly related (*R*^2^ = 0.95), supporting performance-based design charts. Machine learning model (RF) showed that mixture design parameters can serve as a great predictor of UHPC performance, as they predict compressive strength reliably, but they are less accurate for tensile properties and permeability, which depend more on fibre distribution and pore structure.
- Cement dominates the carbon footprint. Increasing cement content beyond ~ 800 kg/m^3^ raises emissions without further strength gain, reflecting limited hydration efficiency (30%–40%) and diminishing contribution to packing density. This shows that maximizing cement content is inefficient after a certain limit (800 kg/m^3^), from a compressive strength and carbon footprint standpoint, which supports the need for an optimized mixture design approach.
- Hybrid binder systems (combining industrial byproducts and bio-sourced SCMs) offer the best trade-off between performance and carbon emissions, achieving up to 200 MPa at 600–900 kg CO_2_/m^3^. Bio-sourced binders minimize emissions but are limited to moderate strengths (120–150 MPa), while industrial byproducts show the widest variability in strength and emissions.
- Low-carbon UHPC design requires optimizing particle packing, SCM selection, and cement content simultaneously rather than in isolation. Continuous packing models, particularly the Modified Andreasen model, best represent UHPC’s graded particle sizes and offer the most practical basis for this optimization compared with discrete or more computationally demanding advanced models.

### Limitations and future research

- Fibre characteristics and pore structure are inconsistently reported in the literature and were not captured in the predictive models, which makes tensile behavior and permeability harder to predict than compressive strength.
- The compiled database also carries heterogeneity typical of literature-based datasets. Curing was controlled through the inclusion criteria (standardized water curing only), but variability in raw material sources, particularly for SCMs and mineral fillers whose composition depends on feedstock and processing, could not be controlled or quantified. This is expected to add scatter to the reported relationships and should be considered when interpreting their strength.
- The dataset of ~ 200 mixtures, while sufficient to identify the main trends reported here, is on the smaller side for machine learning. Therefore, the data-driven and RF results are tied to this dataset and should not be generalized until validated against a larger, more comprehensive database.
- The Random Forest model was limited to mixture design ratios (w/b, c/b, w/c, s/b, s/c), as theoretical packing density (e.g., the Modified Andreasen, De Larrard) could not be calculated due to insufficient particle size data in the source literature. A database with standardized particle size reporting would allow packing density to be included as a predictive feature.
- The embodied carbon analysis relies partly on estimated CO_2_ values and is a cradle-to-gate assessment, excluding transportation, construction, and other life-cycle stages; minor inconsistencies in reported system boundaries across sources cannot be entirely ruled out.
- Future work should prioritize standardized datasets that consistently report microstructural variables (fibre, pore structure, particle size) and adopt broader carbon-accounting boundaries, improving both predictive model accuracy and the reliability of carbon estimates.

## Supplementary Information

Supplementary Material 1

## Data Availability

The data set used and/or analyzed during the current study are available as a supplementary document with this article.

## References

[1] Adesina, A., & Zhang, J. (2024). Impact of concrete structures durability on its sustainability and climate resiliency. *Next Sustainability,**3*, Article 100025. 10.1016/j.nxsust.2024.100025

[2] Lemon, MdMR., Hossain, F., & Imam, MdH. (2026). Experimental Investigation on the Durability Properties of Concrete under Aggressive Environmental Conditions. *Journal of Ceramics and Concrete Sciences*, *11*, 46–70. 10.46610/JoCCS.2026.v011i01.005

[3] Piao, R., Choi, H.-J., Kim, S.-J., Li, W., & Yoo, D.-Y. (2026). Development of ultra-high-performance concrete incorporating nanobubble water and steel slag powder: Mechanical and microstructural optimization. *Cement and Concrete Composites,**166*, Article 106419. 10.1016/j.cemconcomp.2025.106419

[4] Wang, S., Wang, B., Zhu, H., Chen, G., Li, Z., Yang, L., et al. (2023). Ultra-high performance concrete: Mix design, raw materials and curing regimes-A review. *Materials Today Communications,**35*, Article 105468. 10.1016/j.mtcomm.2023.105468

[5] Richard, P., & Cheyrezy, M. (1995). Composition of reactive powder concretes. *Cement and Concrete Research,**25*, 1501–1511. 10.1016/0008-8846(95)00144-2

[6] Moolchandani, K. (2025). Industrial byproducts in concrete: A state-of-the-art review. *Next Materials,**8*, Article 100593. 10.1016/j.nxmate.2025.100593

[7] De Matos, P. R., Sakata, R. D., Gleize, P. J. P., De Brito, J., & Repette, W. L. (2020). Eco-friendly ultra-high performance cement pastes produced with quarry wastes as alternative fillers. *Journal of Cleaner Production,**269*, Article 122308. 10.1016/j.jclepro.2020.122308

[8] Díaz, J., Gálvez, J. C., Alberti, M. G., & Enfedaque, A. (2021). Achieving ultra-high performance concrete by using packing models in combination with nanoadditives. *Nanomaterials,**11*, Article 1414. 10.3390/nano11061414 34071942 PMC8227337

[9] Kwan, A. K. H., & Fung, W. W. S. (2009). Packing density measurement and modelling of fine aggregate and mortar. *Cement and Concrete Composites,**31*, 349–357. 10.1016/j.cemconcomp.2009.03.006

[10] Song, J., Banthia, N., & Yoo, D.-Y. (2025). Effect of supplementary cementitious materials on durability of ultra-high-performance concrete: A review. *Cement and Concrete Composites,**163*, Article 106198. 10.1016/j.cemconcomp.2025.106198

[11] Mandor, A., & Taie, B. (2026). Toward resilient infrastructure: A critical review and roadmap for ultra–high–performance concrete (UHPC) design, performance and standards. *Discovery, Concrete and Cement,**2*, 11. 10.1007/s44416-026-00047-7

[12] Alotaibi, E., Nassif, N., & Alhalabi, M. (2026). Ultra-high-performance concrete for sustainable development goals: A systematic review and conceptual framework. *Journal of Building Engineering,**123*, Article 115802. 10.1016/j.jobe.2026.115802

[13] Ben Chaabene, W., Flah, M., & Nehdi, M. L. (2020). Machine learning prediction of mechanical properties of concrete: Critical review. *Construction and Building Materials,**260*, Article 119889. 10.1016/j.conbuildmat.2020.119889

[14] Young, B. A., Hall, A., Pilon, L., Gupta, P., & Sant, G. (2019). Can the compressive strength of concrete be estimated from knowledge of the mixture proportions?: New insights from statistical analysis and machine learning methods. *Cement and Concrete Research,**115*, 379–388. 10.1016/j.cemconres.2018.09.006

[15] Aylas-Paredes, B. K., Han, T., Neithalath, A., Huang, J., Goel, A., Kumar, A., et al. (2025). Data driven design of ultra high performance concrete prospects and application. *Science and Reports,**15*, 9248. 10.1038/s41598-025-94484-2 PMC11920103 40102603

[16] Dunuweera, S. P., & Rajapakse, R. M. G. (2018). Cement types, composition, uses and advantages of nanocement, environmental impact on cement production, and possible solutions. *Advances in Materials Science and Engineering,**2018*, 1–11. 10.1155/2018/4158682

[17] Dils, J., Boel, V., & De Schutter, G. (2013). Influence of cement type and mixing pressure on air content, rheology and mechanical properties of UHPC. *Construction and Building Materials,**41*, 455–463. 10.1016/j.conbuildmat.2012.12.050

[18] Dhir, R. K., McCarthy, M. J., Zhou, S., & Tittle, P. A. J. (2004). Role of cement content in specifications for concrete durability: Cement type influences. *Proceedings of the Institution of Civil Engineers - Structures and Buildings,**157*, 113–127. 10.1680/stbu.2004.157.2.113

[19] Akhnoukh, A. K., & Buckhalter, C. (2021). Ultra-high-performance concrete: Constituents, mechanical properties, applications and current challenges. *Case Studies in Construction Materials,**15*, Article e00559. 10.1016/j.cscm.2021.e00559

[20] Ciment Québec. (n.d.). Which type of cement to use? Retrieved July 18, 2024 from https://www.cimentquebec.com/en-can/cement/which-cement-to-choose/

[21] Ahmed, T., Elchalakani, M., Karrech, A., Mohamed Ali, M. S., & Guo, L. (2021). Development of ECO-UHPC with very-low-C3A cement and ground granulated blast-furnace slag. *Construction and Building Materials,**284*, Article 122787. 10.1016/j.conbuildmat.2021.122787

[22] Pan, Z., Tan, M., Zheng, G., Wei, L., Tao, Z., & Hao, Y. (2024). Effect of silica fume type on rheology and compressive strength of geopolymer mortar. *Construction and Building Materials,**430*, Article 136488. 10.1016/j.conbuildmat.2024.136488

[23] Lewis, R. C. (2018). Silica fume. In N. De Belie, M. Soutsos, & E. Gruyaert (Eds.), *Properties of fresh and hardened concrete containing supplementary cementitious materials* (Vol. 25, pp. 99–121). Springer International Publishing. 10.1007/978-3-319-70606-1_3

[24] Xi, J., Liu, J., Yang, K., Zhang, S., Han, F., Sha, J., et al. (2022). Role of silica fume on hydration and strength development of ultra-high performance concrete. *Construction and Building Materials,**338*, Article 127600. 10.1016/j.conbuildmat.2022.127600

[25] Zhang, J., Zhao, Y., & Li, H. (2017). Experimental investigation and prediction of compressive strength of ultra-high performance concrete containing supplementary cementitious materials. *Advances in Materials Science and Engineering,**2017*, 1–8. 10.1155/2017/4563164

[26] Amin, M., Zeyad, A. M., Tayeh, B. A., & Saad, A. I. (2022). Effect of ferrosilicon and silica fume on mechanical, durability, and microstructure characteristics of ultra high-performance concrete. *Construction and Building Materials,**320*, Article 126233. 10.1016/j.conbuildmat.2021.126233

[27] Lin, Y., Yan, J., Wang, Z., Fan, F., & Zou, C. (2019). Effect of silica fumes on fluidity of UHPC: Experiments, influence mechanism and evaluation methods. *Construction and Building Materials,**210*, 451–460. 10.1016/j.conbuildmat.2019.03.162

[28] Mazloom, M., Ramezanianpour, A. A., & Brooks, J. J. (2004). Effect of silica fume on mechanical properties of high-strength concrete. *Cement and Concrete Composites,**26*, 347–357. 10.1016/S0958-9465(03)00017-9

[29] Xu, D., Tang, J., Hu, X., Zhou, Y., Yu, C., Han, F., et al. (2023). Influence of silica fume and thermal curing on long-term hydration, microstructure and compressive strength of ultra-high performance concrete (UHPC). *Construction and Building Materials,**395*, Article 132370. 10.1016/j.conbuildmat.2023.132370

[30] Hamada, H. M., Abed, F., Binti Katman, H. Y., Humada, A. M., Al Jawahery, M. S., Majdi, A., et al. (2023). Effect of silica fume on the properties of sustainable cement concrete. *Journal of Materials Research and Technology,**24*, 8887–8908. 10.1016/j.jmrt.2023.05.147

[31] Siddique, R. (2011). Utilization of silica fume in concrete: Review of hardened properties. *Resources, Conservation and Recycling,**55*, 923–932. 10.1016/j.resconrec.2011.06.012

[32] Duan, P., Shui, Z., Chen, W., & Shen, C. (2013). Effects of metakaolin, silica fume and slag on pore structure, interfacial transition zone and compressive strength of concrete. *Construction and Building Materials,**44*, 1–6. 10.1016/j.conbuildmat.2013.02.075

[33] Wardhono, A. (2018). Comparison study of class F and class C fly ashes as cement replacement material on strength development of non-cement mortar. *IOP Conference Series: Materials Science and Engineering,**288*, Article 012019. 10.1088/1757-899X/288/1/012019

[34] Grace, M. A., Clifford, E., & Healy, M. G. (2016). The potential for the use of waste products from a variety of sectors in water treatment processes. *Journal of Cleaner Production,**137*, 788–802. 10.1016/j.jclepro.2016.07.113

[35] Bahedh, M. A., & Jaafar, M. S. (2018). Ultra high-performance concrete utilizing fly ash as cement replacement under autoclaving technique. *Case Studies in Construction Materials,**9*, Article e00202. 10.1016/j.cscm.2018.e00202

[36] Ahmed, T., Elchalakani, M., Karrech, A., Dong, M., Mohamed Ali, M. S., & Yang, H. (2021). ECO-UHPC with high-volume class-F fly ash: New insight into mechanical and durability properties. *Journal of Materials in Civil Engineering,**33*, Article 04021174. 10.1061/(ASCE)MT.1943-5533.0003726

[37] Ferdosian, I., Camões, A., & Ribeiro, M. (2017). High-volume fly ash paste for developing ultra-high performance concrete (UHPC). *Ciência & Tecnologia Dos Materiais,**29*, e157–e161. 10.1016/j.ctmat.2016.10.001

[38] Lu, X. K., Zhang, W., Ruggiero, B. N., Seitz, L. C., & Li, J. (2024). Scalable electrified cementitious materials production and recycling. *Energy & Environmental Science,**17*, 9566–9579. 10.1039/D4EE03529A

[39] Ghafari, E., Ghahari, S. A., Costa, H., Júlio, E., Portugal, A., & Durães, L. (2016). Effect of supplementary cementitious materials on autogenous shrinkage of ultra-high performance concrete. *Construction and Building Materials,**127*, 43–48. 10.1016/j.conbuildmat.2016.09.123

[40] Ahmad, J., Kontoleon, K. J., Majdi, A., Naqash, M. T., Deifalla, A. F., Ben Kahla, N., et al. (2022). A Comprehensive Review on the Ground Granulated Blast Furnace Slag (GGBS) in Concrete Production. *Sustainability,**14*, 8783. 10.3390/su14148783

[41] Yu, R., Spiesz, P., & Brouwers, H. J. H. (2015). Development of an eco-friendly Ultra-High Performance Concrete (UHPC) with efficient cement and mineral admixtures uses. *Cement and Concrete Composites,**55*, 383–394. 10.1016/j.cemconcomp.2014.09.024

[42] Moula, S., Ben Fraj, A., Wattez, T., Bouasker, M., & Hadj Ali, N. B. (2023). Mechanical properties, carbon footprint and cost of ultra-high performance concrete containing ground granulated blast furnace slag. *Journal of Building Engineering,**79*, Article 107796. 10.1016/j.jobe.2023.107796

[43] Moula, S., Ben Fraj, A., Wattez, T., Bouasker, M., & Bel Hadj Ali, N. (2023). The very early-age behaviour of ultra-high performance concrete containing ground granulated blast furnace slag. *Construction and Building Materials,**400*, Article 132630. 10.1016/j.conbuildmat.2023.132630

[44] He, J., Liu, T., Wu, W., Ling, X., & Yu, Q. (2025). Mechanism of SCMs on the hydration, pore structure, and durability of UHPFRC. *Journal of Sustainable Cement-Based Materials,**14*, 1899–1916. 10.1080/21650373.2025.2499063

[45] Zhan, P., Xu, J., Wang, J., & Jiang, C. (2021). Multi-scale study on synergistic effect of cement replacement by metakaolin and typical supplementary cementitious materials on properties of ultra-high performance concrete. *Construction and Building Materials,**307*, Article 125082. 10.1016/j.conbuildmat.2021.125082

[46] Zhang, B., Ji, T., Ma, Y., & Zhang, Q. (2022). Effect of metakaolin and magnesium oxide on flexural strength of ultra-high performance concrete. *Cement and Concrete Composites,**131*, Article 104582. 10.1016/j.cemconcomp.2022.104582

[47] Mo, Z., Gao, X., & Su, A. (2021). Mechanical performances and microstructures of metakaolin contained UHPC matrix under steam curing conditions. *Construction and Building Materials,**268*, Article 121112. 10.1016/j.conbuildmat.2020.121112

[48] Norhasri, M. M. S., Hamidah, M. S., MohdFadzil, A., & Megawati, O. (2016). Inclusion of nano metakaolin as additive in ultra high performance concrete (UHPC). *Construction and Building Materials,**127*, 167–175. 10.1016/j.conbuildmat.2016.09.127

[49] Fadzil, A. M., MuhdNorhasri, M. S., Hamidah, M. S., Zaidi, M. R., & MohdFaizal, J. (2014). Alteration of nano metakaolin for ultra high performance concrete. In R. Hassan, M. Yusoff, Z. Ismail, N. M. Amin, & M. A. Fadzil (Eds.), *InCIEC 2013* (pp. 887–894). Springer Singapore. 10.1007/978-981-4585-02-6_76

[50] Abdellatief, M., AL-Tam, S. M., Elemam, W. E., Alanazi, H., Elgendy, G. M., & Tahwia, A. M. (2023). Development of ultra-high-performance concrete with low environmental impact integrated with metakaolin and industrial wastes. *Case Studies in Construction Materials,**18*, Article e01724. 10.1016/j.cscm.2022.e01724

[51] Tafraoui, A., Escadeillas, G., & Vidal, T. (2016). Durability of the ultra high performances concrete containing metakaolin. *Construction and Building Materials,**112*, 980–987. 10.1016/j.conbuildmat.2016.02.169

[52] Mostafa, S. A., Faried, A. S., Farghali, A. A., EL-Deeb, M. M., Tawfik, T. A., Majer, S., et al. (2020). Influence of nanoparticles from waste materials on mechanical properties, durability and microstructure of UHPC. *Materials,**13*, Article 4530. 10.3390/ma13204530 33066052 PMC7600628

[53] Mo, Z., Wang, R., & Gao, X. (2020). Hydration and mechanical properties of UHPC matrix containing limestone and different levels of metakaolin. *Construction and Building Materials,**256*, Article 119454. 10.1016/j.conbuildmat.2020.119454

[54] Tahwia, A. M., Essam, A., Tayeh, B. A., & Elrahman, M. A. (2022). Enhancing sustainability of ultra-high performance concrete utilizing high-volume waste glass powder. *Case Studies in Construction Materials,**17*, Article e01648. 10.1016/j.cscm.2022.e01648

[55] Wang, Y., Wang, J., Wu, Y., Li, Y., He, X., Su, Y., et al. (2023). Preparation of sustainable ultra-high performance concrete (UHPC) with ultra-fine glass powder as multi-dimensional substitute material. *Construction and Building Materials,**401*, Article 132857. 10.1016/j.conbuildmat.2023.132857

[56] Ghareeb, K. S., Ahmed, H. E., El-Affandy, T. H., Deifalla, A. F., & El-Sayed, T. A. (2022). The novelty of using glass powder and lime powder for producing UHPSCC. *Buildings,**12*, 684. 10.3390/buildings12050684

[57] Li, Z., Zhan, J., Wang, Y., He, Z., & Xie, Y. (2024). Low-carbon UHPC with glass powder and shell powder: Deformation, compressive strength, microstructure and ecological evaluation. *Journal of Building Engineering*. 10.1016/j.jobe.2024.109833

[58] Soliman, N. A., & Tagnit-Hamou, A. (2016). Development of ultra-high-performance concrete using glass powder – Towards ecofriendly concrete. *Construction and Building Materials,**125*, 600–612. 10.1016/j.conbuildmat.2016.08.073

[59] Esmaeili, J., & AL-Mwanes, A. O. (2021). A review: Properties of eco-friendly ultra-high-performance concrete incorporated with waste glass as a partial replacement for cement. *Materials Today: Proceedings,**42*, 1958–1965. 10.1016/j.matpr.2020.12.242

[60] Amin, M., Zeyad, A. M., Agwa, I. S., & Rizk, M. S. (2024). Effect of industrial wastes on the properties of sustainable ultra-high-performance concrete: Ganite, ceramic, and glass. *Construction and Building Materials,**428*, Article 136292. 10.1016/j.conbuildmat.2024.136292

[61] Xu, J., Zhan, P., Zhou, W., Zuo, J., Shah, S. P., & He, Z. (2023). Design and assessment of eco-friendly ultra-high performance concrete with steel slag powder and recycled glass powder. *Powder Technology,**419*, Article 118356. 10.1016/j.powtec.2023.118356

[62] Ferreira, F. G. D. S., Vitoretti Dias, L., Mari Soares, S., & Lorenzetti De Castro, A. (2022). Influence of thermal curing in the physical and mechanical properties of ultra-high-performance concrete with glass powder. *Revista de la Asociación Latinoamericana de Control de Calidad, Patología y Recuperación de la Construcción,**12*, 184–199. 10.21041/ra.v12i2.546

[63] Vaitkevičius, V., Šerelis, E., & Hilbig, H. (2014). The effect of glass powder on the microstructure of ultra high performance concrete. *Construction and Building Materials,**68*, 102–109. 10.1016/j.conbuildmat.2014.05.101

[64] Esmaeili, J., & Oudah AL-Mwanes, A. (2021). A review: Properties of eco-friendly ultra-high-performance concrete incorporated with waste glass as a partial replacement for cement. *Materials Today: Proceedings,**42*, 1958–1965. 10.1016/j.matpr.2020.12.2242

[65] Soliman, N., Omran, A., & Tagnit-Hamou, A. (2019). Effect of Very Fine Ground Glass Pozzolan on Fresh and Mechanical Properties of Ultra-High-Performance Concrete. In: *International Interactive Symposium on Ultra-High Performance Concrete Papers*. Iowa State University Digital Press. 10.21838/uhpc.9631

[66] Ke, G., Li, W., Li, R., Li, Y., & Wang, G. (2018). Mitigation effect of waste glass powders on alkali-silica reaction (ASR) expansion in cementitious composite. *International Journal of Concrete Structures and Materials,**12*, 67. 10.1186/s40069-018-0299-7

[67] Du, J., Wang, Y., Bao, Y., Sarkar, D., & Meng, W. (2023). Valorization of wasted-derived biochar in ultra-high-performance concrete (UHPC): Pretreatment, characterization, and environmental benefits. *Construction and Building Materials,**409*, Article 133839. 10.1016/j.conbuildmat.2023.133839

[68] Dixit, A., Gupta, S., Pang, S. D., & Kua, H. W. (2020). Cement Replacement and Improved Hydration in Ultra-High Performance Concrete Using Biochar. In: Boshoff, W., Combrinck, R., Mechtcherine, V., Wyrzykowski, M. (Eds), *3rd International Conference on the Application of Superabsorbent Polymers (SAP) and Other New Admixtures Towards Smart Concrete *(pp. 222–229). Springer, Cham. 10.1007/978-3-030-33342-3_24

[69] Dixit, A., Verma, A., & Pang, S. D. (2021). Dual waste utilization in ultra-high performance concrete using biochar and marine clay. *Cement & Concrete Composites,**120*, Article 104049. 10.1016/j.cemconcomp.2021.104049

[70] Zhang, Y., He, M., Wang, L., Yan, J., Ma, B., Zhu, X., et al. (2022). Biochar as construction materials for achieving carbon neutrality. *Biochar,**4*, Article 59. 10.1007/s42773-022-00182-x

[71] Winters, D., Boakye, K., & Simske, S. (2022). Toward carbon-neutral concrete through biochar–cement–calcium carbonate composites: A critical review. *Sustainability,**14*, Article 4633. 10.3390/su14084633

[72] Vafaei, B., & Ghahremaninezhad, A. (2024). Effects of biochar as a green additive on the self-healing and properties of sustainable cementitious materials. *ACS Sustainable Chemistry & Engineering,**12*, 8261–8275. 10.1021/acssuschemeng.4c02045

[73] Dixit, A., Gupta, S., Pang, S. D., & Kua, H. W. (2019). Waste valorisation using biochar for cement replacement and internal curing in ultra-high performance concrete. *Journal of Cleaner Production*,* 238*, 117876*.* 10.1016/j.jclepro.2019.117876

[74] Gupta, S., & Kua, H. W. (2019). Carbonaceous micro-filler for cement: Effect of particle size and dosage of biochar on fresh and hardened properties of cement mortar. *Science of The Total Environment,**662*, 952–962. 10.1016/j.scitotenv.2019.01.269 30795482

[75] Mosaberpanah, M. A., & Umar, S. A. (2020). Utilizing Rice Husk Ash as Supplement to Cementitious Materials on Performance of Ultra High Performance Concrete: – A review. *Materials Today Sustainability,**7–8*, Article 100030. 10.1016/j.mtsust.2019.100030

[76] Van, V.-T.-A., Rößler, C., Bui, D.-D., & Ludwig, H.-M. (2014). Rice husk ash as both pozzolanic admixture and internal curing agent in ultra-high performance concrete. *Cement and Concrete Composites,**53*, 270–278. 10.1016/j.cemconcomp.2014.07.015

[77] Abellan-Garcia, J., Martinez, D. M., Khan, M. I., Abbas, Y. M., & Pellicer-Martínez, F. (2023). Environmentally friendly use of rice husk ash and recycled glass waste to produce ultra-high-performance concrete. *Journal of Materials Research and Technology,**25*, 1869–1881. 10.1016/j.jmrt.2023.06.041

[78] Alyami, M., Hakeem, I. Y., Amin, M., Zeyad, A. M., Tayeh, B. A., & Agwa, I. S. (2023). Effect of agricultural olive, rice husk and sugarcane leaf waste ashes on sustainable ultra-high-performance concrete. *Journal of Building Engineering,**72*, Article 106689. 10.1016/j.jobe.2023.106689

[79] Huang, H., Gao, X., Wang, H., & Ye, H. (2017). Influence of rice husk ash on strength and permeability of ultra-high performance concrete. *Construction and Building Materials,**149*, 621–628. 10.1016/j.conbuildmat.2017.05.155

[80] Ha, N. S., Marundrury, S. S., Pham, T. M., Pournasiri, E., Shi, F., & Hao, H. (2022). Effect of grounded blast furnace slag and rice husk ash on performance of ultra-high-performance concrete (UHPC) subjected to impact loading. *Construction and Building Materials,**329*, Article 127213. 10.1016/j.conbuildmat.2022.127213

[81] Faried, A. S., Mostafa, S. A., Tayeh, B. A., & Tawfik, T. A. (2021). The effect of using nano rice husk ash of different burning degrees on ultra-high-performance concrete properties. *Construction and Building Materials,**290*, Article 123279. 10.1016/j.conbuildmat.2021.123279

[82] Kang, S.-H., Hong, S.-G., & Moon, J. (2019). The use of rice husk ash as reactive filler in ultra-high performance concrete. *Cement and Concrete Research,**115*, 389–400. 10.1016/j.cemconres.2018.09.004

[83] Ngo, T. V., Tran, V. B., Le, B. H., Dang, H. T., Matos, J., Tran, M. Q., et al. (2024). An assessment of the impact of locally recycled cementitious replacement materials on the strength of the ultra-high-performance concrete. *Applied Sciences,**14*, Article 7484. 10.3390/app14177484

[84] Wang, X., Yu, R., Shui, Z., Song, Q., & Zhang, Z. (2017). Mix design and characteristics evaluation of an eco-friendly ultra-high performance concrete incorporating recycled coral based materials. *Journal of Cleaner Production,**165*, 70–80. 10.1016/j.jclepro.2017.07.096

[85] He, Z., Han, X., Shi, J., Aslani, F., Gencel, O., & Du, S. (2022). Multi-scale characteristics of eco-friendly marine binder using coral waste. *Powder Technology,**403*, Article 117395. 10.1016/j.powtec.2022.117395

[86] He, Z., Shen, M., Shi, J., Yalçınkaya, Ç., Du, S., & Yuan, Q. (2022). Recycling coral waste into eco-friendly UHPC: Mechanical strength, microstructure, and environmental benefits. *Science of the Total Environment,**836*, Article 155424. 10.1016/j.scitotenv.2022.155424 35504383

[87] He, Z., Ni, Y., Zhang, Y., Shi, J., Revilla-Cuesta, V., Hu, Y., et al. (2022). Mechanical properties, nanoscale characteristics, and environmental analysis of high-volume waste coral powder mortar (HVCM). *Powder Technology,**407*, Article 117613. 10.1016/j.powtec.2022.117613

[88] Wang, D., Shi, C., Farzadnia, N., Shi, Z., Jia, H., & Ou, Z. (2018). A review on use of limestone powder in cement-based materials: Mechanism, hydration and microstructures. *Construction and Building Materials,**181*, 659–672. 10.1016/j.conbuildmat.2018.06.075

[89] Ma, R., Zhang, L., Song, Y., Lin, G., Qian, X., & Qian, K. (2024). Recycling stone powder as a partial substitute for cementitious materials into eco-friendly manufactured sand ultra-high performance concretes: Towards cleaner and more cost-effective construction production. *Journal of Building Engineering,**92*, Article 109772. 10.1016/j.jobe.2024.109772

[90] Huang, Y., Chen, Q., Shang, H., Wang, J., & Song, N. (2024). Development of sustainable ultra-high-performance concrete (UHPC) by synergistic utilization of red mud and limestone powder. *Journal of Building Engineering,**90*, Article 109372. 10.1016/j.jobe.2024.109372

[91] Chen, K., Cheng, S., Wu, Q., Chen, X., Zhao, C., Li, S., et al. (2024). Utilization of recycled fine aggregate in ultra-high performance concrete: Mechanical strength, microstructure and environment impacts. *Construction and Building Materials,**439*, Article 137364. 10.1016/j.conbuildmat.2024.137364

[92] Zhang, X. Y., Fan, M. X., Zhou, Y. X., Ji, D. D., Li, J. H., & Yu, R. (2023). Development of a sustainable alkali activated ultra-high performance concrete (A-UHPC) incorporating recycled concrete fines. *Journal of Building Engineering,**67*, Article 105986. 10.1016/j.jobe.2023.105986

[93] Shorthouse, P. (2021). The circular economy & Canada’s built environment. Circular Economy Leadership Canada 2021. Retrieved July 19, 2024, from https://circulareconomyleaders.ca/resources/the-circular-economy-canadas-built-environment/

[94] Yu, L., Huang, L., & Ding, H. (2019). Rheological and mechanical properties of ultra-high-performance concrete containing fine recycled concrete aggregates. *Materials,**12*, Article 3717. 10.3390/ma12223717 31717969 PMC6888130

[95] Pavlů, T., Fořtová, K., Mariaková, D., Řepka, J., Vlach, T., & Hájek, P. (2023). High-performance concrete with fine recycled concrete aggregate: Experimental assessment. *Structural Concrete,**24*, 1868–1878. 10.1002/suco.202200734

[96] Sierens. Z., Joseph. M., Vandevyvere. B., & Li. J. (2018). *High Quality Recycled Concrete Aggregates in UHPC: A Preliminary Study* [Conference paper presentation]. International Conference Progress of Recycling in the Built Environment, Lisbon, Portugal.

[97] Zhang, H., Ji, T., Zeng, X., Yang, Z., Lin, X., & Liang, Y. (2018). Mechanical behavior of ultra-high performance concrete (UHPC) using recycled fine aggregate cured under different conditions and the mechanism based on integrated microstructural parameters. *Construction and Building Materials,**192*, 489–507. 10.1016/j.conbuildmat.2018.10.117

[98] Nassar, R.-U.-D., Zaid, O., & Elhadi, K. M. (2025). Utilizing carbonated recycled concrete fines to develop sustainable ultra-high-performance fiber-reinforced concrete. *Journal of Building Engineering,**99*, Article 111634. 10.1016/j.jobe.2024.111634

[99] Suchithra, S., Sowmiya, M., & Pavithran, T. (2022). Effect of ceramic tile waste on strength parameters of concrete-a review. *Materials Today: Proceedings,**65*, 975–982. 10.1016/j.matpr.2022.03.610

[100] Al-Fakih, A., Odeh, A., Mahamood, M., Al-Shugaa, M., Al-Osta, M., & Ahmad, S. (2023). Review of the Properties of Sustainable Cementitious Systems Incorporating Ceramic Waste. *Buildings,**13*, 2105. 10.3390/buildings13082105

[101] Jain, P., Gupta, R., & Chaudhary, S. (2022). A literature review on the effect of using ceramic waste as supplementary cementitious material in cement composites on workability and compressive strength. *Materials Today: Proceedings,**65*, 871–876. 10.1016/j.matpr.2022.03.453

[102] Li, L., Liu, W., You, Q., Chen, M., & Zeng, Q. (2020). Waste ceramic powder as a pozzolanic supplementary filler of cement for developing sustainable building materials. *Journal of Cleaner Production,**259*, Article 120853. 10.1016/j.jclepro.2020.120853

[103] Zhang, L., Bian, M., Xiao, Z., Wang, Y., Xu, K., Han, B., et al. (2024). Using nano-CaCO _3_ and ceramic tile waste to design low-carbon ultra high performance concrete. *Nanotechnology Reviews,**13*, 20230198. 10.1515/ntrev-2023-0198

[104] Zhang, L., Shen, H., Xu, K., Huang, W., Wang, Y., Chen, M., et al. (2023). Effect of ceramic waste tile as a fine aggregate on the mechanical properties of low-carbon ultrahigh performance concrete. *Construction and Building Materials,**370*, Article 130595. 10.1016/j.conbuildmat.2023.130595

[105] Xu, K., Huang, W., Zhang, L., Fu, S., Chen, M., Ding, S., et al. (2021). Mechanical properties of low-carbon ultrahigh-performance concrete with ceramic tile waste powder. *Construction and Building Materials,**287*, Article 123036. 10.1016/j.conbuildmat.2021.123036

[106] Amin, M., Tayeh, B. A., & Agwa, I. S. (2020). Effect of using mineral admixtures and ceramic wastes as coarse aggregates on properties of ultrahigh-performance concrete. *Journal of Cleaner Production,**273*, Article 123073. 10.1016/j.jclepro.2020.123073

[107] Kannan, D. M., Aboubakr, S. H., EL-Dieb, A. S., & Reda Taha, M. M. (2017). High performance concrete incorporating ceramic waste powder as large partial replacement of Portland cement. *Construction and Building Materials,**144*, 35–41. 10.1016/j.conbuildmat.2017.03.115

[108] LópezBoadella, Í., LópezGayarre, F., Suárez González, J., Gómez-Soberón, J. M., López-Colina Pérez, C., Serrano López, M., et al. (2019). The Influence of Granite Cutting Waste on The Properties of Ultra-High Performance Concrete. *Materials,**12*, 634. 10.3390/ma12040634 30791583 PMC6416720

[109] Hakeem, I. Y., Amin, M., Agwa, I. S., Rizk, M. S., & Abdelmagied, M. F. (2023). Effect of using sugarcane leaf ash and granite dust as partial replacements for cement on characteristics of ultra-high performance concrete. *Case Studies in Construction Materials,**19*, Article e02266. 10.1016/j.cscm.2023.e02266

[110] Xiong, X., Wu, M., Shen, W., Li, J., Zhao, D., Li, P., et al. (2022). Performance and microstructure of ultra-high-performance concrete (UHPC) with silica fume replaced by inert mineral powders. *Construction and Building Materials,**327*, Article 126996. 10.1016/j.conbuildmat.2022.126996

[111] Zhang, H., Ji, T., He, B., & He, L. (2019). Performance of ultra-high performance concrete (UHPC) with cement partially replaced by ground granite powder (GGP) under different curing conditions. *Construction and Building Materials,**213*, 469–482. 10.1016/j.conbuildmat.2019.04.058

[112] Hou, D., Wu, D., Wang, X., Gao, S., Yu, R., Li, M., et al. (2021). Sustainable use of red mud in ultra-high performance concrete (UHPC): Design and performance evaluation. *Cement and Concrete Composites,**115*, Article 103862. 10.1016/j.cemconcomp.2020.103862

[113] Yan, P., Chen, B., Aminul Haque, M., & Liu, T. (2023). Influence of red mud on the engineering and microstructural properties of sustainable ultra-high performance concrete. *Construction and Building Materials,**396*, Article 132404. 10.1016/j.conbuildmat.2023.132404

[114] Wang, Y., Zhang, T., Lyu, G., Guo, F., Zhang, W., & Zhang, Y. (2018). Recovery of alkali and alumina from bauxite residue (red mud) and complete reuse of the treated residue. *Journal of Cleaner Production,**188*, 456–465. 10.1016/j.jclepro.2018.04.009

[115] Liu, R.-X., & Poon, C.-S. (2016). Utilization of red mud derived from bauxite in self-compacting concrete. *Journal of Cleaner Production,**112*, 384–391. 10.1016/j.jclepro.2015.09.049

[116] Ling, G., Shui, Z., Gao, X., Sun, T., Yu, R., & Li, X. (2021). Utilizing Iron Ore Tailing as Cementitious Material for Eco-Friendly Design of Ultra-High Performance Concrete (UHPC). *Materials,**14*, 1829. 10.3390/ma14081829 33917148 PMC8067869

[117] Zhang, W., Gu, X., Qiu, J., Liu, J., Zhao, Y., & Li, X. (2020). Effects of iron ore tailings on the compressive strength and permeability of ultra-high performance concrete. *Construction and Building Materials,**260*, Article 119917. 10.1016/j.conbuildmat.2020.119917

[118] Gu, X., Zhang, W., Zhang, X., Li, X., & Qiu, J. (2022). Hydration characteristics investigation of iron tailings blended ultra high performance concrete: The effects of mechanical activation and iron tailings content. *Journal of Building Engineering,**45*, Article 103459. 10.1016/j.jobe.2021.103459

[119] Huang, X., Ranade, R., & Li, V. C. (2013). Feasibility Study of Developing Green ECC Using Iron Ore Tailings Powder as Cement Replacement. *Journal of Materials in Civil Engineering,**25*, 923–931. 10.1061/(ASCE)MT.1943-5533.0000674

[120] Zhao, Y., Gu, X., Qiu, J., Zhang, W., & Li, X. (2021). Study on the utilization of iron tailings in ultra-high-performance concrete: Fresh properties and compressive behaviors. *Materials,**14*, Article 4807. 10.3390/ma14174807 34500898 PMC8432546

[121] Zhu, Z., Li, B., & Zhou, M. (2015). The influences of iron ore tailings as fine aggregate on the strength of ultra-high performance concrete. *Advances in Materials Science and Engineering,**2015*, 1–6. 10.1155/2015/412878

[122] Zhao, S., Fan, J., & Sun, W. (2014). Utilization of iron ore tailings as fine aggregate in ultra-high performance concrete. *Construction and Building Materials,**50*, 540–548. 10.1016/j.conbuildmat.2013.10.019

[123] Zhou, M., Wu, Z., Ouyang, X., Hu, X., & Shi, C. (2021). Mixture design methods for ultra-high-performance concrete - A review. *Cement and Concrete Composites,**124*, Article 104242. 10.1016/j.cemconcomp.2021.104242

[124] De Grazia, M. T., Sanchez, L. F. M., & Yahia, A. (2023). Towards the design of eco-efficient concrete mixtures: An overview. *Journal of Cleaner Production,**389*, Article 135752. 10.1016/j.jclepro.2022.135752

[125] Furnas, C. C. (1931). Grading aggregates - I. - Mathematical relations for beds of broken solids of maximum density. *Industrial & Engineering Chemistry,**23*, 1052–1058. 10.1021/ie50261a017

[126] Zheng, J., Carlson, W. B., & Reed, J. S. (1995). The packing density of binary powder mixtures. *Journal of the European Ceramic Society,**15*, 479–483. 10.1016/0955-2219(95)00001-B

[127] Fennis‐Huijben, SAAM. (2011). *Design of Ecological Concrete by Particle Packing Optimization* [Doctoral dissertation, Delft University of Technology].

[128] Toufar, W., Born, M., & Klose, E. (1976). *Beitrag zur Optimierung der Packungsdichte Polydisperser körniger Systeme* (pp. 29–44). VEB Deutscher Verlag Für Grundstoffindustrie.

[129] Goltermann P, Johansen V, Palbøl L. Packing of Aggregates: An Alternative Tool to Determine the Optimal Aggregate Mix. Materials Journal 1997;94:435–43. 10.14359/328.

[130] Stovall, T., De Larrard, F., & Buil, M. (1986). Linear packing density model of grain mixtures. *Powder Technology,**48*, 1–12. 10.1016/0032-5910(86)80058-4

[131] De Larrard, F. (1988). *Formulation et propriétés des bétons à très hautes performances*. Central Laboratory of Bridges and Highways, France.

[132] De Larrard, F., & Sedran, T. (1994). Optimization of ultra‑high‑performance concrete by the use of a packing model. *Cement and Concrete Research*, *24*(6), 997–1009. 10.1016/0008-8846(94)90022-1

[133] De Larrard, F. (1999). *Concrete mixture proportioning: A scientific approach* (p. 448). CRC Press. 10.1201/9781482272055

[134] Fuller, W. B., & Thompson, S. E. (1907). The laws of proportioning concrete. *Transactions of the American Society of Civil Engineers*. 10.1061/TACEAT.0001979

[135] Andreasen, A. H. M. (1930). Ueber die Beziehung zwischen Kornabstufung und Zwischenraum in Produkten aus losen Körnern (mit einigen Experimenten). *Kolloid-Zeitschrift,**50*, 217–228. 10.1007/BF01422986

[136] Funk, J. E., & Dinger, D. R. (1994). *Predictive process control of crowded particulate suspensions*. Springer US. 10.1007/978-1-4615-3118-0

[137] Wan, Y., Li, L., Zou, J., Xiao, H., Zhu, M., Su, Y., et al. (2024). Effect of shrinkage reducing agent and steel fiber on the fluidity and cracking performance of ultra-high performance concrete. *FDMP-Fluid Dynamics Materials Processing,**20*, 1941–1956. 10.32604/fdmp.2024.053910

[138] Wu, Z., Khayat, K. H., & Shi, C. (2019). Changes in rheology and mechanical properties of ultra-high performance concrete with silica fume content. *Cement and Concrete Research,**123*, Article 105786. 10.1016/j.cemconres.2019.105786

[139] Tran, T. M., Trinh, H. T. M. K., Nguyen, D., Tao, Q., Mali, S., & Pham, T. M. (2023). Development of sustainable ultra-high-performance concrete containing ground granulated blast furnace slag and glass powder: Mix design investigation. *Construction and Building Materials,**397*, Article 132358. 10.1016/j.conbuildmat.2023.132358

[140] Venkatesh, C., Nerella, R., & Chand, M. S. R. (2019). Experimental investigation of strength, durability, and microstructure of red-mud concrete. *Journal of the Korean Ceramic Society,**57*, 167–174. 10.1007/s43207-019-00014-y

[141] Bahmani, H., & Mostofinejad, D. (2022). Microstructure of ultra-high-performance concrete (UHPC) – A review study. *Journal of Building Engineering,**50*, Article 104118. 10.1016/j.jobe.2022.104118

[142] Jhatial, A. A., Nováková, I., & Gjerløw, E. (2023). A review on emerging cementitious materials, reactivity evaluation and treatment methods. *Buildings,**13*, 526. 10.3390/buildings13020526

[143] Cheruvu, R., & Rao, B. K. (2024). Enhanced concrete performance and sustainability with fly ash and ground granulated blast furnace slag – A comprehensive experimental study. *Advances in Science and Technology Research Journal,**18*, 161–174. 10.12913/22998624/186192

[144] Hamad, M. A., Nasr, M., Shubbar, A., Al-Khafaji, Z., Al Masoodi, Z., Al-Hashimi, O., et al. (2021). Production of ultra-high-performance concrete with low energy consumption and carbon footprint using supplementary cementitious materials instead of silica fume: A review. *Energies,**14*, Article 8291. 10.3390/en14248291

[145] Li, P. P., Brouwers, H. J. H., Chen, W., & Yu, Q. (2020). Optimization and characterization of high-volume limestone powder in sustainable ultra-high performance concrete. *Construction and Building Materials,**242*, Article 118112.

[146] Park, S., Wu, S., Liu, Z., & Pyo, S. (2021). The role of supplementary cementitious materials (SCMs) in ultra high performance concrete (UHPC): A review. *Materials,**14*, Article 1472. 10.3390/ma14061472 33802943 PMC8002722

[147] Du, J., Wang, Y., Guo, P., & Meng, W. (2024). Tailoring of steel fiber surface by coating cellulose nanocrystal for enhanced flexural properties of UHPC. *Cement & Concrete Composites,**154*, Article 105773. 10.1016/j.cemconcomp.2024.105773

[148] Beskopylny, A. N., Stel’makh, S. A., Shcherban’, E. M., Mailyan, L. R., Meskhi, B., Smolyanichenko, A. S., et al. (2022). High-performance concrete nanomodified with recycled rice straw biochar. *Applied Sciences,**12*, Article 5480. 10.3390/app12115480

[149] Ouyang, X., Shi, C., Wu, Z., Li, K., Shan, B., & Shi, J. (2020). Experimental investigation and prediction of elastic modulus of ultra-high performance concrete (UHPC) based on its composition. *Cement and Concrete Research,**138*, Article 106241. 10.1016/j.cemconres.2020.106241

[150] Hasgül, U., & Biçakçioğlu, N. (2022). Ultra yüksek performanslı betonda çimento yerine cam tozu ve/veya yüksek fırın cürufunun kullanılabilirliği. *Gazi Üniversitesi Mühendislik Mimarlık Fakültesi Dergisi,**37*, 2241–2258. 10.17341/gazimmfd.868527

[151] Nikbin, I. M., Aliaghazadeh, M., Charkhtab, S., & Fathollahpour, A. (2018). Environmental impacts and mechanical properties of lightweight concrete containing bauxite residue (red mud). *Journal of Cleaner Production*, *172*, 2683–2694. 10.1016/j.jclepro.2017.11.143

[152] Venkatesh, C., Ruben, N., & Chand, M. S. R. (2020). Red mud as an additive in concrete: Comprehensive characterization. *Journal of the Korean Ceramic Society,**57*, 281–289. 10.1007/s43207-020-00030-3

[153] Tang, W. C., Wang, Z., Liu, Y., & Cui, H. Z. (2018). Influence of red mud on fresh and hardened properties of self-compacting concrete. *Construction and Building Materials,**178*, 288–300. 10.1016/j.conbuildmat.2018.05.171

[154] Kanyal, K. S., Agrawal, Y., & Gupta, T. (2021). Properties of sustainable concrete containing red mud: A review.* Journal of Scientific Research and Reports*, *27*(3), 15–26. 10.9734/jsrr/2021/v27i330366

[155] Abbas, S., Nehdi, M. L., & Saleem, M. A. (2016). Ultra-High Performance Concrete: Mechanical Performance, Durability, Sustainability and Implementation Challenges. *Int J Concr Struct Mater,**10*, 271–295. 10.1007/s40069-016-0157-4

[156] Wang, M., & Yao, H. (2020). A Novel Organic-Inorganic Hybrid Admixture for Increasing Flowability and Reducing Viscosity of Ultra-High Performance Paste. *Materials,**13*, 3385. 10.3390/ma13153385 32751683 PMC7435950

[157] El-Helou, R. G., Haber, Z. B., & Graybeal, B. A. Mechanical behavior and design properties of ultra‑high‑performance concrete (Open Source). *Materials Journal*, *119*(1), 181–194. 10.14359/51734194

[158] García, J. A., Castellanos, N. T., Gomez, J. A. F., & Lopez, A. M. N. (2021). Ultra-high-performance concrete with local high unburned carbon fly ash. *DYNA,**88*, 38–47. 10.15446/dyna.v88n216.89234

[159] Hasan, T. M., Gilbert, L., Allena, S., Owusu-Danquah, J., & Torres, A. (2022). Development of Non-Proprietary Ultra-High Performance Concrete Mixtures. *Buildings,**12*, 1865. 10.3390/buildings12111865

[160] Chang, W., & Zheng, W. (2020). Effects of key parameters on fluidity and compressive strength of ultra‐high performance concrete. *Structural Concrete,**21*, 747–760. 10.1002/suco.201900167

[161] Voo, Y. L., & Foster, S. J. (2010). Characteristics of ultra-high performance ‘ductile’ concrete and its impact on sustainable construction. *The IES Journal Part A: Civil & Structural Engineering,**3*, 168–187. 10.1080/19373260.2010.492588

[162] Liu, Q., Hu, Z., Lu, X., Yang, J., Azim, I., & Sun, W. (2020). Prediction of Chloride Distribution for Offshore Concrete Based on Statistical Analysis. *Materials,**13*, 174. 10.3390/ma13010174 31906365 PMC6982216

[163] Ibrahim, M., Bahraq, A. A., Salami, B. A., Alhems, L. M., Hussaini, S. R., Nasir, M., et al. (2025). Pore Structure Characterization and Environmental Assessment of Ground Volcanic Pumice-Based Alkali-Activated Concrete. *International Journal of Concrete Structures and Materials,**19*, 60. 10.1186/s40069-025-00791-3

[164] Li, L., Zou, Y., Jiang, Y., Zhou, J., Yang, J., & Zhang, Z. (2025). Durability of micro-cracked UHPC under the effects of loads and environments: A state of the art review. *Case Studies in Construction Materials,**23*, Article e05433. 10.1016/j.cscm.2025.e05433

[165] Liu, X., & Zhang, M.-H. (2010). Permeability of high-performance concrete incorporating presoaked lightweight aggregates for internal curing. *Magazine of Concrete Research,**62*, 79–89. 10.1680/macr.2008.62.2.79

[166] Hu, Y. J., Du, Y. L., & Zhang, W. (2011). Evaluation of relationship between innitial current, conductance, chloride diffusion coefficient and charge passed of concrete. *Advanced Materials Research,**399–401*, 1200–1203. 10.4028/www.scientific.net/AMR.399-401.1200

[167] Xin-e, Y. (2021). Study on chloride ion diffusion characteristics of reactive powder concrete under different loads. In: Safuan, B, A. Rashid, A., Liu, A. (Eds), *E3S Web of Conferences*, *283*, 01015. 10.1051/e3sconf/202128301015

[168] Ye, H., Jin, X., Fu, C., Jin, N., Xu, Y., & Huang, T. (2016). Chloride penetration in concrete exposed to cyclic drying-wetting and carbonation. *Construction and Building Materials,**112*, 457–463. 10.1016/j.conbuildmat.2016.02.194

[169] Yang, Y. B., Lai, Z. Q., Ai, L. T., Guo, W. Y., Huang, H. J., & Wang, H. C. (2014). Experimental Research on the Evaluation Method of the Resistance to Chloride Ion Penetration of Autoclaved PHC Pile. *KEM,**629–630*, 587–592. 10.4028/www.scientific.net/KEM.629-630.587

[170] Yoon, Y.-S., An, G.-H., Koh, K.-T., & Ryu, G.-S. (2025). Mechanical performance and shrinkage behavior of ultrahigh-performance concrete with ferronickel slag under various curing conditions. *Buildings,**15*, Article 3670. 10.3390/buildings15203670

[171] Kothari, A., Rajczakowska, M., & Cwirzen, A. (2022). UHPC overlay as sustainable solution to preserve old concrete structures. *MATEC Web Conf,**364*, 04014. 10.1051/matecconf/202236404014

[172] Wang, B., Wang, L., & Li, M. (2007). Experimental research on the autogenous shrinkage of MK high performance concrete. *J Wuhan Univ Technol,**22*, 551–554. 10.1007/s11595-006-3551-y

[173] Huang, H., & Ye, G. (2017). Examining the “time-zero” of autogenous shrinkage in high/ultra-high performance cement pastes. *Cement and Concrete Research,**97*, 107–114. 10.1016/j.cemconres.2017.03.010

[174] Huang, W., Zhang, T., & Xu, Y. Y. (2013). Research progress on early autogenous shrinkage of cement-based cementitious materials. *Applied Mechanics Reviews,**634*, 2738–2741. 10.4028/www.scientific.net/AMR.634-638.2738

[175] Ma, J., & Dehn, F. (2017). Shrinkage and creep behavior of an alkali‐activated slag concrete. *Structural Concrete,**18*, 801–810. 10.1002/suco.201600147

[176] Cui, S. A., Ye, Y. Z., Dou, S. T., & Fu, F. (2012). Study on frost resistance of high performence concrete resistance to chloride ion. *Acta Mechanica Sinica,**174–177*, 1312–1316. 10.4028/www.scientific.net/AMM.174-177.1312

[177] Farnam, Y., Bentz, D., Hampton, A., & Weiss, W. J. (2014). Acoustic emission and low-temperature calorimetry study of freeze and thaw behavior in cementitious materials exposed to sodium chloride salt. *Transportation Research Record: Journal of the Transportation Research Board,**2441*, 81–90. 10.3141/2441-11

[178] Li, Z. Y. (2012). Evaluation of the Bearing Capacity of the Concrete Pier Exposed to the Freeze-Thaw Cycles. *AMM,**166–169*, 1216–1221. 10.4028/www.scientific.net/AMM.166-169.1216

[179] Fan, Y. F., Zhang, S. Y., & Shah, S. P. (2016). Influence of Nanoclay on Concrete Subjected to Freeze-Thaw Cycles and Bond Behavior between Rebar and Concrete. *KEM,**711*, 256–262. 10.4028/www.scientific.net/KEM.711.256

[180] Gao, S., & Liu, L. (2024). Effects of Freeze-Thaw Cycles on Axial Compression Behaviors of UHPC-RC Composite Columns. *Materials,**17*, 1843. 10.3390/ma17081843 38673200 PMC11051503

[181] Alrashidi, R. S., Voss, M. S., Alsubeai, A., Alshammari, E., & Riding, K. A. (2025). Evaluation of Non-Proprietary Ultra-High-Performance Concrete (UHPC) to Resistance of Freeze-Thaw. *CivilEng,**6*, 57. 10.3390/civileng6040057

[182] Zhu, H., Yang, S., Li, W., Li, Z., Fan, J., & Shen, Z. (2020). Study of Mechanical Properties and Durability of Alkali-Activated Coal Gangue-Slag Concrete. *Materials,**13*, 5576. 10.3390/ma13235576 33297535 PMC7729631

[183] Mehta, P. K. (1983). Mechanism of sulfate attack on portland cement concrete — Another look. *Cement and Concrete Research,**13*, 401–406. 10.1016/0008-8846(83)90040-6

[184] Santhanam, M., Cohen, M. D., & Olek, J. (2002). Mechanism of sulfate attack: A fresh look: Part 1: Summary of experimental results. *Cement and Concrete Research,**32*, 915–921. 10.1016/S0008-8846(02)00724-X

[185] Santhanam, M., Cohen, M. D., & Olek, J. (2003). Mechanism of sulfate attack: A fresh look: Part 2. *Proposed mechanisms. Cement and Concrete Research,**33*, 341–346. 10.1016/S0008-8846(02)00958-4

[186] Neville, A. (2004). The confused world of sulfate attack on concrete. *Cement and Concrete Research,**34*, 1275–1296. 10.1016/j.cemconres.2004.04.004

[187] Juenger, M. C. G., & Siddique, R. (2015). Recent advances in understanding the role of supplementary cementitious materials in concrete. *Cement and Concrete Research,**78*, 71–80. 10.1016/j.cemconres.2015.03.018

[188] Graybeal, B. (2011).* Ultra-High Performance Concrete*. Federal Highway Administration Research and Technology.

[189] Wang, Y., Yang, Z., Quan, Z., Zhang, S., Niu, D., & Wu, J. (2025). Sulfate resistance and life prediction of UHPC in western saline soil environment. *Construction and Building Materials,**458*, Article 139756. 10.1016/j.conbuildmat.2024.139756

[190] Matos, A. M. (2022). Susceptibility to expansive reactions of a greener UHPC: Micro to macro-scale study. *Applied Sciences,**12*, Article 6252. 10.33990/app12126252

[191] Adams, M. P., & Ideker, J. H. (2020). Using Supplementary Cementitious Materials to Mitigate Alkali-Silica Reaction in Concrete with Recycled-Concrete Aggregate. *Journal of Materials in Civil Engineering,**32*, 04020209. 10.1061/(ASCE)MT.1943-5533.0003277

[192] Stanton, T. E. (1942). Expansion of concrete through reaction between cement and aggregate. *Transactions of the American Society of Civil Engineers,**107*, 54–84. 10.1061/TACEAT.0005540

[193] Thomas, M. (2011). The effect of supplementary cementing materials on alkali-silica reaction: A review. *Cement and Concrete Research,**41*, 1224–1231. 10.1016/j.cemconres.2010.11.003

[194] Möser, B., & Stark, J. (2008). Durability and microstructural development during hydration in ultra‑high performance concrete. In: M. G. Alexander, H.‑D. Beushausen, F. Dehn, & P. Moyo (Eds.), *Concrete Repair, Rehabilitation and Retrofitting II*. CRC Press.

[195] Guang, Y, & Nguyen, V. T. (2012). Mitigation of autogenous shrinkage of ultra‑high performance concrete by rice husk ash.* Journal of the Chinese Ceramic Society*, *40*(2), 212–216.

[196] Viyasun, K., Anuradha, R., Thangapandi, K., Santhosh Kumar, D., Sivakrishna, A., & Gobinath, R. (2021). Investigation on performance of red mud based concrete. *Materials Today: Proceedings,**39*, 796–799. 10.1016/j.matpr.2020.09.637

[197] Amin, M., Agwa, I. S., Mashaan, N., Mahmood, S., & Abd-Elrahman, M. H. (2023). Investigation of the Physical Mechanical Properties and Durability of Sustainable Ultra-High Performance Concrete with Recycled Waste Glass. *Sustainability,**15*, 3085. 10.3390/su15043085

[198] Amin, M., Zeyad, A. M., Tayeh, B. A., & Saad, A. I. (2021). Effects of nano cotton stalk and palm leaf ashes on ultrahigh-performance concrete properties incorporating recycled concrete aggregates. *Construction and Building Materials,**302*, Article 124196. 10.1016/j.conbuildmat.2021.124196

[199] Awadeen, M., Amin, M., Bakr, R. H., & Tahwia, A. M. (2024). Mechanical properties, attenuation coefficient, and microstructure of ultra high-performance heavyweight concrete for radiation shielding applications. *Journal of Building Engineering,**82*, Article 108395. 10.1016/j.jobe.2023.108395

[200] Ghanim, A. A. J., Amin, M., Zeyad, A. M., Tayeh, B. A., & Agwa, I. S. (2023). Effect of modified nano-titanium and fly ash on ultra-high-performance concrete properties. *Structural Concrete,**24*, 6815–6832. 10.1002/suco.202300053

[201] Hakeem, I. Y., Amin, M., Agwa, I. S., Abd-Elrahman, M. H., Ibrahim, O. M. O., & Samy, M. (2023). Ultra-high-performance concrete properties containing rice straw ash and nano eggshell powder. *Case Studies in Construction Materials,**19*, Article e02291. 10.1016/j.cscm.2023.e02291

[202] European Commission, Joint Research Centre. (2015). European reference life‑cycle database.

[203] Singh, R., & Patel, R. (2023). Strength and durability performance of rice straw ash-based concrete: An approach for the valorization of agriculture waste. *International Journal of Environmental Science and Technology*. 10.1007/s13762-022-04554-5

[204] Long, G., Gao, Y., & Xie, Y. (2015). Designing more sustainable and greener self-compacting concrete. *Construction and Building Materials,**84*, 301–306. 10.1016/j.conbuildmat.2015.02.072

[205] Tiong, H. Y., Lim, S. K., Lee, Y. L., Ong, C. F., & Yew, M. K. (2020). Environmental impact and quality assessment of using eggshell powder incorporated in lightweight foamed concrete. *Construction and Building Materials*. 10.1016/j.conbuildmat.2020.118341

[206] Haque, N., & Norgate, T. (2013). Estimation of greenhouse gas emissions from ferroalloy production using life cycle assessment with particular reference to Australia. *Journal of Cleaner Production,**39*, 220–230. 10.1016/j.jclepro.2012.08.010

[207] Al-Akhras, N. M., Al-Akhras, K. M., & Attom, M. F. (2009). Performance of olive waste ash concrete exposed to elevated temperatures. *Fire Safety Journal*. 10.1016/j.firesaf.2008.08.006

[208] Rathanasalam, V. S., Hithani, B., Rajendra, M. B., Raikhola, A. S., & Bisalahalli, A. (2024). Brief review on cotton plant stalk ash-based concrete. *Recent advances in building materials and technologies* (Vol. 456, pp. 271–281). Springer Nature. 10.1007/978-981-99-9458-8_25

[209] Alnahhal, M. F., Alengaram, U. J., Jumaat, M. Z., Abutaha, F., Alqedra, M. A., & Nayaka, R. R. (2018). Assessment on engineering properties and CO2 emissions of recycled aggregate concrete incorporating waste products as supplements to Portland cement. *Journal of Cleaner Production,**203*, 822–835. 10.1016/j.jclepro.2018.08.292

[210] Shi, Y., Long, G., Ma, C., Xie, Y., & He, J. (2019). Design and preparation of ultra-high performance concrete with low environmental impact. *Journal of Cleaner Production,**214*, 633–643. 10.1016/j.jclepro.2018.12.318

[211] Chiaia, B., Fantilli, A. P., Guerini, A., Volpatti, G., & Zampini, D. (2014). Eco-mechanical index for structural concrete. *Construction and Building Materials,**67*, 386–392. 10.1016/j.conbuildmat.2013.12.090

[212] Hou, W., Zhang, Q., Zhuang, Z., & Zhang, Y. (2024). Sustainable reusing marble powder and granite powder in cement-based materials: A review. *ACS Sustainable Chemistry & Engineering,**12*, 2484–2510. 10.1021/acssuschemeng.3c06670

[213] Siddique, S., Chaudhary, S., Shrivastava, S., & Gupta, T. (2019). Sustainable utilisation of ceramic waste in concrete: Exposure to adverse conditions. *Journal of Cleaner Production,**210*, 246–255. 10.1016/j.jclepro.2018.10.231

[214] Heidari, S. M., & Anctil, A. (2022). Country-specific carbon footprint and cumulative energy demand of metallurgical grade silicon production for silicon photovoltaics. *Resources, Conservation and Recycling,**180*, Article 106171. 10.1016/j.resconrec.2022.106171

[215] Farchich, M. (2024). *Functionality of recycled concrete fines: An experimental study on the mechanical performance of recycled concrete fines at mortar level *[Master’s thesis, Delft University of Technology].

[216] Ecoinvent Centre. (2014). The ecoinvent LCA database. Swiss Centre for Life Cycle Inventories.

[217] Flower, D. J. M., & Sanjayan, J. G. (2007). Green house gas emissions due to concrete manufacture. *International Journal of Life Cycle Assessment,**12*, 282–288. 10.1065/lca2007.05.327

